# Reconstructing the degree of mammal defaunation throughout the Caatinga - the largest dry tropical forest region of South America

**DOI:** 10.1371/journal.pone.0336562

**Published:** 2025-11-24

**Authors:** Nathália Fernandes Canassa, Carlos A. Peres, Célia Cristina Clemente Machado, Helder Farias P. Araujo

**Affiliations:** 1 Center of Exact Sciences and Nature, Graduate Program of Biological Sciences (Zoology), Federal University of Paraíba, João Pessoa, Paraíba, Brazil; 2 Postgraduate Program of Biodiversity and Conservation, Federal University of Piauí, Floriano, Piauí, Brazil; 3 School of Environmental Sciences, University of East Anglia, Norwich, United Kingdom; 4 Instituto Juruá, Manaus, Brazil; 5 Center of Applied Biological and Social Sciences, State University of Paraiba, João Pessoa, Paraíba, Brazil; University of Florida, UNITED STATES OF AMERICA

## Abstract

Mammal diversity around the world has been increasingly threatened by a myriad of anthropogenic drivers, but particularly overhunting and natural habitat loss. These threats alter the structure of local mammal assemblages and, consequently, their associated ecological interactions. Here, we assess the degree to which the mammal fauna has been defaunated across the 862,818-km^2^ Caatinga tropical dry forest region of northeastern Brazil. Specifically, we examine potential changes in the structure of medium- to large-bodied mammal assemblages, large-scale spatial patterns of local extinctions, the loss of ecosystem functionality, and the role of human disturbance and protected areas in mammal defaunation. We compiled empirical data for 51 species representing a total of 73 local mammal assemblages that could be defined as exhaustively sampled throughout the region and compared species distribution estimates between contemporary and historical times. Our results show that 90% of the Caatinga lost between 20% and 80% of its mammal species, and the structure of coexisting local assemblages was further downsized by ~77%. Among all 51 species, 37 lost over 50% of their geographic range across the region. Caatinga defaunation is currently associated with a severe loss of ecosystem functionality. Overhunting, agropastoral habitat conversion and ruminant livestock were the main drivers of the extent and severity of local defaunation rates, which were conversely buffered by protected areas. This study informs conservation efforts in arid tropical forest regions dominated by the rural poor, including protection of remnant dry forests and restoration of mammal- and habitat- mediated ecosystem services.

## Introduction

The Anthropocene is characterized by profound human impacts on Earth’s natural landscapes, mainly leading to biodiversity loss, as manifested by extinctions at local to regional and even global scales [1]. Over the last 300 years, some 50% of Earth’s natural tree cover has been lost [2]. Moreover, 515 highly threatened terrestrial vertebrate species hold global populations of fewer than 1,000 individuals, 30% of which in South America, and 48 of these species lost 95% of their global scale geographic distribution [3]. This vertebrate diversity loss affects ecological key functions, such as seed dispersal, pollination, and prey population control via top-down regulation [4]. Defaunation is defined as the decline or extirpation of animal populations induced mainly by anthropogenic activities [1]. Some of these activities result in natural habitat loss due to land use change driven by food and fiber commodity demand, afforestation, shifting agriculture, and urbanization [5]. Land use change not only subdivides and reduces the area and quality of remnant habitats but also alters the structure and composition of ecological communities, ultimately leading to biodiversity declines [6,7]. In addition to habitat loss, defaunation also results from direct human overexploitation for subsistence, trade, or sport [8]. The results about direct human overexploitation are increasingly recognized as a key driver of biodiversity loss in tropical forests and is closely linked to the “empty forest” syndrome, where habitats may appear structurally intact but are severely depleted of large vertebrates [9,10].

One of several phenomena related to population declines and extinctions is the “downsizing effect” [11], in which species or individuals within an assemblage on average become smaller over time. Assemblage downsizing results from widespread human propensity to target large-bodied species [12], but depletion of larger animals from an assemblage may result in partial density compensation by smaller- bodied specie [11]. Likewise, predictable selection by hunters of the largest individuals within a population can result in a shift in the gene pool towards faster life-histories and smaller-bodied individuals [13]. Over time, both processes can lead to predictable changes in the average size of individuals within an assemblage. This overall downsizing can have pervasive ecological consequences, since large-bodied vertebrates often play essential and irreplaceable roles in ecosystems as apex predators or ecosystem engineers, influencing the dynamics of their habitats, disrupting ecological relationships and leading to cascading effects on overall ecosystem structure and function [4,14]. For example, heavily hunted “half-empty” areas in Amazonian forests on average host vertebrate biomass densities of 200 kg/km^2^, compared to >700 kg/km^2^ in non-hunted areas [15]. Prey body mass is a key indicator of hunter preference, as larger animals provide more meat per unit of energy and time expenditure while pursuing prey, and are often associated with high economic returns [12]. Therefore, studies on the effects of human harvesting have shown a global reduction in the average body size of contemporary mammal assemblages [8], particularly considering the Pleistocene fossil record in continents settled by humans late in the human dispersal prehistory, like the Americas [16].

In semi-arid regions, declines in mammal abundance and species diversity could be attributed to anthropogenic drivers such as dry forest conversion into livestock pastures and croplands, which are further aggravated by ever drier conditions as predicted by global climate trends [17]. In these xeric regions, the combination of land use intensification and climate change often reduces overall primary productivity as a response to markedly low precipitation [18], further suppressing habitat carrying capacity to sustain large animals. In addition to the degradation of natural landscapes, these arid regions are typically occupied by chronically deprived rural poor who exert significant hunting pressure for both commerce and subsistence. In the Brazilian semi-arid region known as the Caatinga, for example, subsistence hunting typically subsidizes the economy of poor rural households and/or is associated with commercial and recreational practices [19]. Overhunting has already induced regional scale extinctions of large mammals such as the white-lipped peccary (*Tayassu pecari*), and widespread local extinctions of large game rodents such as paca (*Cuniculus paca*) [20,21].

Although ~89% of the original vegetation cover in the Caatinga has been converted into other land uses [22], hunting remains a pervasive extractive activity [21,23], resulting in high rates of defaunation [13]. However, significant knowledge gaps persist concerning the magnitude, species composition and geographic patterns of large-bodied vertebrate losses, the magnitude of assemblage downsizing, and the underlying factors contributing to regional defaunation. Here, we assess (1) the magnitude and spatial extent of defaunation of all known species of medium- to large-bodied mammals (MLBM) across the entire Brazilian Caatinga, measured by the difference between contemporary and historical assemblages reconstructed using species distribution models; (2) the resulting changes in terms of the size structure of contemporary mammal assemblages; (3) the extent to which the geographic range of these species has been reduced; (4) whether there is a loss of ecological function in contemporary assemblages due to defaunation, and (5) the relative importance of both positive and negative environmental drivers of defaunation. Finally, we discuss the effects of regional scale defaunation on the conservation status of different species and likely functional extinctions that may affect the natural resilience of Caatinga ecosystems.

## Materials and methods

### Study area

The Caatinga is a semiarid region in northeastern Brazil covering 862,818 km^2^, which represents approximately 10% of the country’s territory [24]. It is known as the main seasonally dry tropical forest (SDTF) in South America, with a high richness of endemic plant species [25]. The vegetation of the Caatinga is highly heterogeneous and comprises four main physiognomic types: Tropical Seasonal Dry Forest, which dominates most of the region; enclaves of Tropical Rain Forests, located in montane ranges; Savannas, occurring on low-nutrient, acidic soils; and Rupestrian Grassland, limited to a section of the Chapada Diamantina in Bahia state [26]. The region experiences marked variation in annual precipitation ranging from 400 to 1200 mm, resulting in distinct local climates, with more arid environments in the central region and wetter portions along coastal and montane areas, due to the east-west orographic effect of oceanic moisture [27]. Approximately 94% of the Caatinga is now at risk of desertification due to severe human disturbance [28], including livestock farming, croplands, as well as fuelwood and mineral extraction, all of which are the main drivers of environmental degradation across the region [29].

### Study species and defaunation estimates

We selected MLBM species that occur across the Caatinga based on their body mass, home range size, and trophic level. To do so, we conducted a bibliographic survey using the Google Scholar, Scielo, Web of Science, and ScienceDirect databases, employing word combinations (in English and Portuguese) such as “medium- to large-bodied mammals”, “checklist”, “Caatinga”, “hunting”, and “ethnofauna” to access any available information on MLBM assemblages across the Caatinga. We also used the Global Biodiversity Information Facility (GBIF – https://www.gbif.org) and the Specieslink platform (https://specieslink.net) to supplement spatially explicit species-occurrence data. From the compiled records, we grouped georeferenced species that co-occurred at the same localities and selected assemblages with an overall richness of at least five species. Selection was informed by contemporary literature demonstrating that even mammal-poor sites retain a minimum level of richness [30], and by field records collected by the authors. This process resulted in the compilation of 73 mammal assemblages distributed throughout the region, amounting to a total of 51 species (S1 Table).

We estimated a measure of defaunation by comparing two spatially explicit distributions for each of the selected mammal species. First, we generated a potential distribution surface using species distribution models (SDMs) calibrated with current environmental variables over South America. Because these models reflect the environmental suitability for species occurrence irrespective of human intervention, we interpret this distribution as a proxy for pre-colonial conditions (ca. 1500 AD, the onset of Brazil’s colonial period). Second, we estimated contemporary distributions using inverse distance weighting (IDW) interpolation based on species records across the Caatinga, with occurrence data collected as detailed in the previous paragraph. Defaunation was then calculated by subtracting the contemporary raster from the potential (pre-colonial) raster. Details on how these species-specific rasters were generated are provided below.

#### Historical distribution.

The potential historical distribution of the 51 mammal species examined here was obtained from SDM based on contemporary climatic and topographic conditions [31]. SDMs have been recommended to reconstruct past, present and future species distributions, using only a subset of the observations; in our case, geographic coordinates representing occurrence records. Among the 51 species examined here, some occur across a wide range of continental-scale climatic conditions. Therefore, occurrence data were selected from across South America (DOI:10.17632/bdmzzytkdm.1) and later cropped to match the regional boundaries of the Caatinga domain [24].

SDMs are powerful tools for ecological and biogeographical studies, as they allow researchers to estimate potential species distributions based on environmental predictors [32]. These models assume that species distributions are largely determined by abiotic factors, such as climate and topography, which shape habitat suitability. This is particularly relevant for mammal species, as climate and elevation are major drivers of their distribution patterns, influencing their patterns of habitat selection, resource availability, and overall ecological niche [33].

One of the key advantages of using SDMs is their ability to predict species distributions beyond areas containing direct occurrence records, thereby providing insights into regions that may have been historically suitable but are currently unoccupied due to anthropogenic change [34]. SDMs thus enable the reconstruction of historical distributions by excluding contemporary land-use effects, allowing us to infer species ranges under pre-anthropogenic conditions. Following this approach, we estimated the unadulterated historical distribution of these species, unaffected by land use and land cover change; in other words, free from the constraints imposed by post-colonial habitat modifications.

It is important to note that SDMs essentially estimate the species’ fundamental niche, since dispersal limitations and biotic interactions are not explicitly represented. This may lead to commission errors, especially for habitat specialists. To minimize this risk, we used occurrence data from the full South American range of each species and cropped them to the Caatinga domain, while also selecting ecologically meaningful climatic and topographic predictors. Our aim was therefore not to reproduce realized distributions under historical constraints, but to provide a robust approximation of the fundamental niche under suitable abiotic conditions. The supporting information provides additional information on the protocol of how to build a model (S1 File and S2 Table).

To achieve this, we used 19 climate variables along with elevation data extracted from WorldClim version 2.1 (www.worldclim.org), at a spatial resolution of 2.5 arcminutes (≈ 4,630 m). WorldClim provides high-resolution interpolated climate data, derived from 9,000–60,000 weather stations worldwide, aggregated across a target temporal range of 1970–2000. The dataset was generated using thin-plate spline interpolation, incorporating elevation, distance to the coast, and satellite-derived variables. WorldClim applies regionalized modelling, selecting the best-performing model for each region and variable, ensuring high accuracy [35]. This methodology offers robust and globally consistent climate estimates, making it well-suited for species distribution modelling.

To avoid redundancy, we included in the models all variables that were correlated with each other by less than 0.8, using a Pearson correlation matrix calculated using the R package “vegan” (version 3.5.3). At the end of this process, we considered the following 10 variables: diurnal amplitude of mean temperature (BIO2), temperature seasonality (BIO4), mean temperature of the wettest (BIO8) and driest (BIO09) quarter, annual precipitation (BIO12), precipitation seasonality (BIO15), precipitation of the driest (BIO17), warmest (BIO18) and coldest (BIO19) quarter, and terrain elevation. We used the Maximum Entropy v.3.4.1 (MaxEnt) algorithm [36] to generate the modeled geographic distributions of each selected species. MaxEnt models comprise a probability distribution in which each grid cell predicts the suitability of conditions for each species [36]. We used Receiver Operator Characteristic (ROC) statistics to assess model accuracy, with 10 replicates of 10,000 maximum iterations; 10% of the average replicates were randomized as test data, while the remainder were randomized to train the model during each replicate. Each species predictive map was transformed into a binary map (0: absent, 1: presence) that was thresholded using MaxEnt thresholds (S3 Table). We selected the thresholds that defined the smallest potential habitat following a conservative approach to avoid overestimating species geographic distributions (S1–S5 Figs). To obtain the final raster layer representing potential species richness and composition under historical conditions, we summed the presence values across the collective overlay of all binary species maps (S1–S5 Figs).

#### Contemporary distribution.

Based on the georeferenced points of the 73 reliable present-day mammal assemblages found in this study for the Caatinga region, we performed an interpolation using the inverse distance weighting (IDW) method to generate a continuous probability surface (raster) of contemporary species richness across the entire region. IDW is a mathematical method that estimates a value for an unsampled location using the average of data values within a neighborhood weighted by the inverse of the distance between any interpolated points.

### Size structure and species composition

We compiled information on the adult body mass of all 51 selected species examined here from the literature [37,38]. To estimate changes in body mass distributions over time, we created two raster layers, one representing contemporary aggregate body mass. obtained by summing the body mass of all species currently co-occurring in each of the 73 local assemblages, and another for historical aggregate body mass, reconstructed by first determining the historical species composition in each assemblage using the binary maps of their likely historical distributions and then summing the body mass of all species predicted to have coexisted in the past. Changes in size structure were calculated by comparing the historical and contemporary aggregate body mass distributions for each assemblage, with downsizing defined as a reduction in the mean body mass of species from the historical to the contemporary period within the same locality. Following the same logic, changes in the species composition of each assemblage were also compared between historical and contemporary analogues for each species.

### Functional diversity

To examine whether there was a loss in contemporary ecosystem function measured through functional richness [39], we characterized the 73 mammal assemblages in terms of the following functional traits of each co-occurring species: body mass (g), feeding guild (hypercarnivore, insectivore, myrmecophague, omnivore, piscivore, frugivore, folivore, gummivore, and granivore), and locomotion mode (terrestrial, semi-fossorial, scansorial, semi-aquatic and arboreal) [37], attributing the two latter traits to either absence or presence (0 or 1). From these data we assessed whether defaunation had a positive effect on the loss of functional diversity (FD) in the ecosystem of each contemporary assemblage. To assess functional diversity, we first categorized the types of traits selected. Our data include both continuous (body mass) and binary variables (presence or absence), so we used the Gower measure as the functional distance method [40]. The result generated is a species distance matrix, was then used in the joint analysis with species community data using the “dbFD” R package [39]. This package uses principal coordinate analysis (PCoA), based on a matrix of previously generated functional attributes, which are used as ‘features’ to calculate FD. For this analysis, we used a value of m = 4, which indicates the number of PCoA axes with an R^2^ > 0.75, indicating a good sample of functional characteristics to calculate FD. Thus, historical functional diversity was based on the presence of each modelled co-occurring species at each of the 73 localities, whereas the contemporary functional diversity was based on the presence of contemporary species at the same sites. Functional diversity loss is therefore defined as the subtraction between the historical and the contemporary FD values.

### Predictors of defaunation and functional diversity

To assess the drivers of defaunation and potential refuges for mammal assemblages in the Caatinga, we considered two sets of complementary variables. Factors likely to promote defaunation included human-induced habitat loss and disturbance, such as agropastoral land use, livestock density, crop and pasture areas, and the Human Footprint index. Conversely, factors potentially mitigating defaunation included legally protected areas, remnants of native vegetation in private lands, and aboveground biomass, which support local species richness. Details on each variable are provided below.

Agropastoral land use (croplands and grazelands) has been the main driver of natural vegetation conversion throughout the Caatinga, especially since the rapid 16^th^ century conquest of the bovine cattle economic cycle [41], so we used the extent of land allocated to livestock farming as a regional scale indicator of habitat loss. We therefore assumed that areas containing larger numbers of livestock and farm establishments are likely associated with higher levels of natural habitat loss across the Caatinga [22]. To this end, we used the proportion of livestock enterprises (ls); proportion of natural and cultivated pastures (past); and proportion of annual and perennial crop landholdings (crop) per municipal county as an indicator of habitat loss. The 862,818-km^2^ Caatinga region is comprised of 1,210 municipal counties (mean ± SD size = 713 ± 783 km^2^) embedded within 120 administrative microregions, thereby providing a reasonable spatial resolution for those variables. Those variables represent the proportion of rural enterprises in each county allocated to livestock husbandry, exotic pastures, and croplands relative to the overall number of agricultural establishments, obtained from Brazil’s 2017 agricultural census. Considering the above predictors as one of the most consequential historical human disturbances, we also seek to understand how other forms of human disturbance could impact the mammal fauna. To do this, we used human footprint data, which provides a global map of human pressure measured on the basis of eight variables, including built-up environments, population density, energy infrastructure, croplands, pastures, roads, railways, and paved and unpaved roads. We used the 2009 human footprint map, which has a spatial resolution of approximately 1 km^2^ [42].

In addition, it is critical to understand the spatial distribution of suitable habitat availability, as we expected a higher local mammal species packing in the most intact sites containing the highest aboveground vegetation biomass. To assess habitats that may protect local faunas and potentially preclude defaunation, we also examined the availability of suitable habitats using as proxied by the sum of legally protected areas (PAs) in the state and private domains and aboveground biomass. We grouped the management hierarchy of different denominations of protected areas (known in Brazil as Conservation Units) as follows: 1) Fully Protected Conservation Units (FPCUs), 2) Sustainable Use Conservation Units (SUCUs), and 3) natural vegetation set asides within private landholdings, known as Permanent Preservation Areas (PPA). Data on protected areas were extracted from the Ministry of the Environment (MMA), which provides vectors of protected areas managed at the federal, state, and municipal levels for both fully protected and sustainable use reserves. To identify and map PPAs, we considered terrain slopes greater than 45°, as sanctioned by the Brazilian Forest Law (Law 12.651, 25^th^ May 2012; Brazil, 2012), because the remnants of native vegetation on private land in the Caatinga are predominantly restricted to these areas due to limited accessibility for mechanized agriculture. Native vegetation remnants within private areas throughout the Caatinga are predominantly restricted to these areas due to limited accessibility for mechanized agriculture. We used a digital elevation model (DEM) from the Shuttle Radar Topography Mission (SRTM) with a spatial resolution of 1 arc-sec (30 m) to generate a slope map using the spatial analysis tools in QGIS, Madeira version 4.4.14. Data on aboveground vegetation biomass density (Mg/ha) were extracted from Global Forest Watch (https://data.globalforestwatch.org/datasets/gfw::aboveground-live-woody-biomass-density/about). This global benchmark map of aboveground biomass density for the year 2000 at 30-m spatial resolution is the result of integrating ground measurements, airborne and spaceborne LiDAR data with satellite images. The basic approach follows the one developed to map tropical biomass at both 500-m and 30-m resolution [43].

### Data analysis

We employed a hexagonal grid system with cells measuring 5 km in edge length (hexcell area = 64.95 km^2^), amounting to ~12,662 hexcells covering the entire extent of the Caatinga. We extracted several metrics for each hexcell describing potential drivers of defaunation, including the cumulative proportion and the mean of the human footprint, aboveground biomass (Mg/ha), and the total area of all protected areas (m^2^). As a response variable, we used the defaunation rate, here defined as the historical-to-contemporary difference in species richness in each hexcell across the Caatinga. We also examined the degree to which the size structure of mammal assemblages changed from historical to modern times, for which we consider our assemblage-level downsizing metric. All vector and matrix spatial analyses were performed using the open-source software QGIS, Madeira version 4.4.14.

To assess the relative importance of each of the predictors on defaunation of MLBM across the Caatinga, we employed a Gradient Boosted Machine (GBM) algorithm, specifically the Stochastic Gradient Boosting (‘gbm’ in the *caret* R package [44], which generates predictive models based on regression decision trees. These models are effective to model complex ecological systems because they accommodate both continuous and categorical predictors, missing data and outliers; do not require prior data transformation; and capture complex non-linear relationships. To evaluate model performance, we used accuracy (R^2^) and RMSE (Root Mean Square Error) values. We split all data into a training and test dataset (75% and 25%, respectively). We parameterized the GBM model using a resampling method, and the random search links with a 10-fold cross-validation. The training dataset was shuffled randomly and split into 10 groups. Random search exhaustively generates candidates from a range of given parameter values, and models are fit to the training data for all possible combinations of parameter values. The model performance for all parameter combinations was evaluated based on the test dataset and the best parameter combination was maintained. To interpret the gbm output, we used SHAP (SHapley Additive explanations) values and plotted the contribution trends of each variable (positive or negative) in the model obtained, using the *shapviz* R package [45].

Finally, a generalized linear model (GLM) was generated to explore which predictors, such as vegetation biomass, human footprint, and protected areas, also influenced the rate of functional diversity loss, using our metric of lost functional diversity across the 73 localities. We also used linear models (LMs) to examine how defaunation affects changes in functional diversity. As our response variables are continuous data, we used Gaussian distributions for both models. Models and graphics were generated using the “mass” and “ggplot2” packages in the R platform.

## Results

### Geographic patterns of defaunation

**Fig 1:**
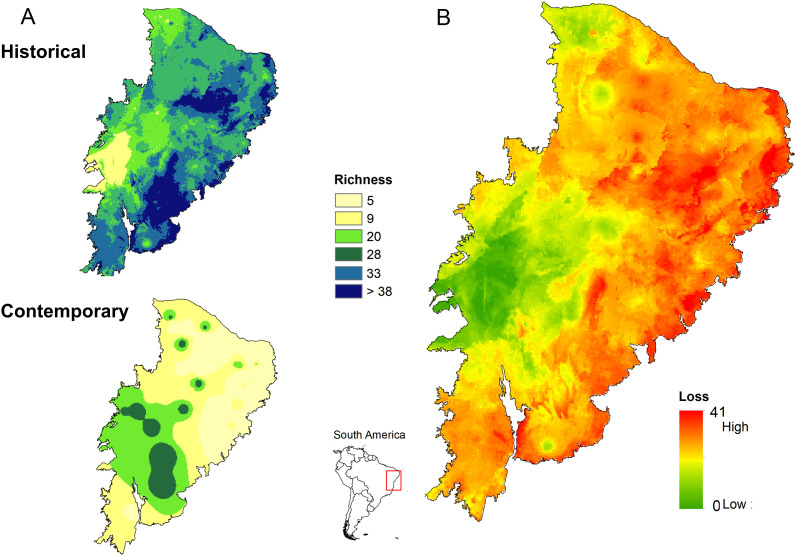
Distribution of (A) historical and contemporary estimated species richness of 51 medium- to large-bodied mammal species across the entire Caatinga dry forest of northeastern Brazil. (B) Defaunation rates of these mammal species, ranging from 0 to 41 species, across the Caatinga as defined by the difference between reconstructed historical assemblages and observed contemporary assemblages. This map was prepared by the first author using the QGIS Madeira version 4.4.14 program and the geographic limits such as the limit of Caatinga and South America were taken from public database data from the Brazilian Institute of Geography and Statistics (IBGE https://www.ibge.gov.br/geociencias/organizacao-do-territorio/malhas-territoriais/15774-malhas.html).

The 51 focal mammal species show an average contemporary local richness of only 13 co-occurring species across the entire Caatinga region (SD = 3.9; range 5 ‒ 33) Projections from historical assemblages showed a maximum local richness of 48 species (mean 33.0 ± 6.0; range 9 ‒ 48). Our defaunation estimates indicate that, on average, 20 species (39%) were lost at each site, but this estimate was as high as 41 species (80%). This pattern of species loss was pervasive, covering a wide swath of the Caatinga region. For instance, 18% of the region lost over 50% of all species (range = 26–41 species) and 72% of the region lost 20–49% of all species (range = 10–25 species). The highest rates of species extirpations were observed in the eastern, central-eastern, and central-northern subregions of Caatinga (Fig 1B). Subregions experiencing low to moderate rates of defaunation contained a larger number of strictly protected reserves, including the Serra das Confusões National Park, Boqueirão das Onças National Park, Ubajara National Park, Serra das Almas Natural Reserve and Serra da Capivara National Park (Fig 1A). However, even these five ‘best-case’ protected areas still lost 34 mammal populations (mean ± SD = 6.8 ± 1.5 mammal species, *N* = 5).

### Downsizing and compositional changes

**Fig 2:**
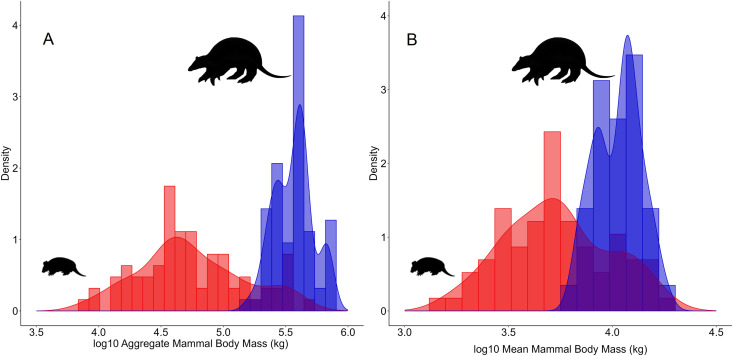
Aggregate mammal body mass of medium to large-bodied mammals per assemblage generated from potential species distribution models (blue histogram) and observed contemporary mammal assemblages (red histogram) across the Caatinga region. (A) Sum of body mass of all co-occurring mammal species; and (B) Mean body mass of all co-occurring mammal species.

Considering historical assemblages, total mammal biomass for any given site was on average 389.6 ± 139.7 kg (range = 160.1–707.6 kg) but declined to only 90.1 ± 99.7 kg (range = 7.7–468.9 kg) in modern-day assemblages (Fig 2), which amounted to a total loss in aggregate mammal biomass of 21,866 kg across the 73 sites (299.5 ± 133.3 kg per site). Localities sustaining the highest contemporary aggregate mammal biomass clearly matched existing protected areas, which also hosted the highest species richness. These results indicate that the local mammal biomass on average declined by over three-quarters (–77.2%) across the Caatinga region, which was induced by both a reduction in the number of sympatric species and a systematically biased pattern of extirpation that largely penalized large-bodied species.

**Fig 3:**
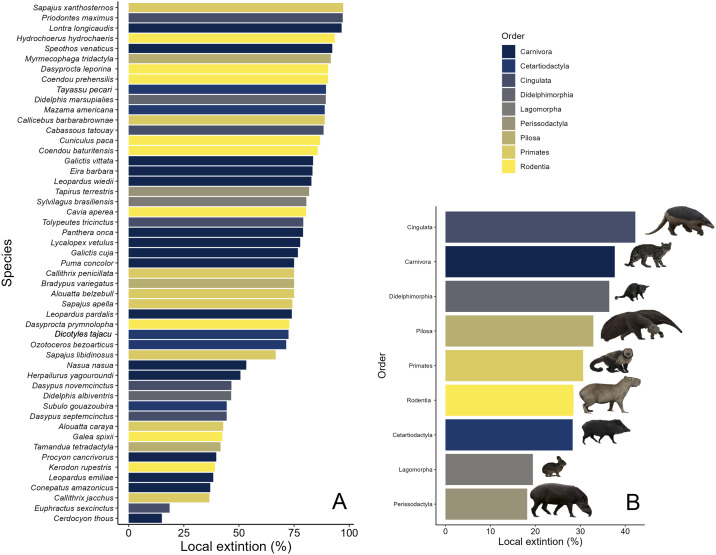
Local extinction rates of medium- to large-bodied mammal species (A) and (B) orders across 73 mammal assemblages distributed throughout the Caatinga dry forest. Bars are colour-coded according to species taxonomic order. Both species and orders are listed top to bottom from the highest to the lowest local extinction rates.

Among all 51 mammal species surveyed, a staggering 37 lost more than 50% in their overall occurrence area throughout the Caatinga. To estimate these losses, we compared the potential historical distribution of each species, based on the number of assemblages it was expected to occupy, to its contemporary distribution across the same assemblages. These proportions were then extrapolated to the total area historically occupied by each species, allowing us to calculate the corresponding reduction in occurrence area. On average, these 37 species experienced a reduction 295,201 km^2^ (± 401,331 km^2^, range = 1,212–582,557 km^2^–, *N *= 37). The critically endangered buff-headed capuchin monkey (*Sapajus xanthosternos*) and giant armadillo (*Priodontes maximus,* vulnerable) showed the highest rates of geographic range shrinkage (95% each; Fig 3). Important game species that were historically overhunted such as lowland tapir (*Tapirus terrestris*), white-lipped peccary (*Tayassu pecari*), and paca (*Cuniculus paca*) were extirpated in over 80% of their predicted distribution areas. Even seemingly ubiquitous non-forest species, such as the crab-eating fox (*Cerdocyon thous*) and yellow armadillo (*Euphractus sexcinctus*), incurred extensive areas of extirpation, albeit at relatively lower rates (Fig 3). As perissodactyls and lagomorphs are represented by a single species each, lowland tapir (*Tapirus terrestris*) and the tapeti (*Sylvilagus brasiliensis*), respectively, these mammalian orders lost the largest proportional areas of occurrence (81% and 80%, respectively). In addition, these species succumbed to a long history of hunting (Fig 3).

**Fig 4:**
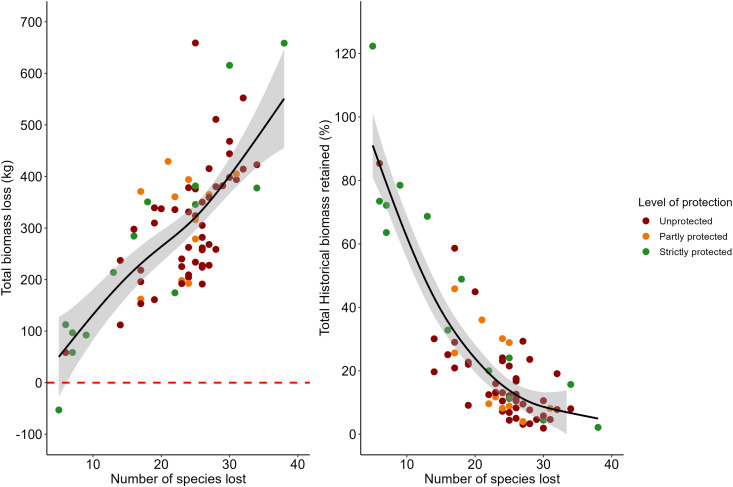
Number of mammal species lost in the set of 73 assemblages distributed across dry forest areas of the Caatinga. Each point refers to the set of assemblages with degrees of contemporary enforced protection based on (A) total biomass loss (kg) and (B) the total proportion of historical biomass retained at the time of each mammal inventory. The red dashed line separates losses from biomass gains. The gray area represents the confidence band around the black line generated by the generalized additive model (GAM).

Considering the contemporary status of protection of each mammal assemblage, both unprotected sites (*N *= 48) and partly protected multiple-use reserves (*N *= 11) on average retained less than 20% of their historical aggregate biomass (mean ± SE: 16.9 ± 2.2% and 19.7 ± 4.2%, respectively). However, strictly protected areas (*N *= 14) retained a much larger fraction (45.6 ± 9.5%) of the original biomass, which was significantly higher than less protected sites (Tukey-Kramer HSD, p-values < 0.05 in both cases). Despite this nominal protection effect (see Fig 4), all sites had lost meaningful numbers of large mammal species, not least because historical local extirpations in the Caatinga region date back to the 16^th^ century, whereas all Caatinga protected areas are relatively recent, having been created since 1946.

### Changes in functional diversity

**Fig 5:**
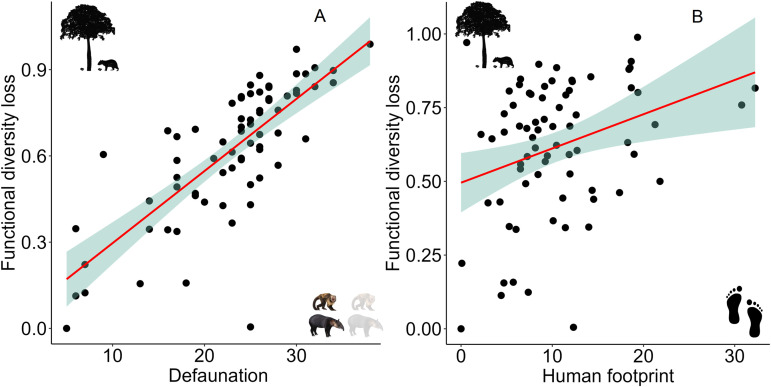
Loss in functional diversity across 73 mammal assemblages of the Caatinga semi-arid region of South America. A) Relationship between functional diversity loss and defaunation rate according to the model generated. B) Relationship between functional diversity loss and the footprint of human disturbance. Both predictors are significant (p < 0.05) according to each model.

Of the 73 sites assessed here, 72 showed a decline in functional diversity; the only exception was one of the ‘best’ strictly protected areas of the Caatinga (Serra das Confusões National Park). Historical functional diversity loss across assemblages ranged between 0.5 and 1.0, whereas contemporary functional diversity loss ranged between 0.0 and 0.6, of which two reserves (Morro do Chapéu and Chapada Diamantina) retained the highest present-day functional diversity (0.8 for both). Among the predictors we investigated, only human footprint showed a significantly positive relationship with functional diversity loss, and the steepest losses in functional diversity were observed at sites exposed to the highest human footprint values (Table 1; Fig 5). Not surprisingly, there was also a positive relationship between loss of functional diversity and level of defaunation; in other words, functional diversity was lowest at the most impoverished sites (Table 1; Fig 5).

**Table 1:** Values generated by a general mixed-effects model, assessing whether aboveground biomass (AGB), protected areas, level of human footprint and defaunation influences functional diversity loss. P-values below the critical significance value (α = 0.05) are shown in bold.

Loss in functional diversity			
Predictors	estimate	std	p
AGB	> 0.01	> 0.01	0.92
Protected areas	> −0.01	> −0.01	0.21
Human footprint	> 0.01	> 0.04	**0.02**
Defaunation	0.02	0.02	**0.00**

### Predictors of defaunation

**Fig 6:**
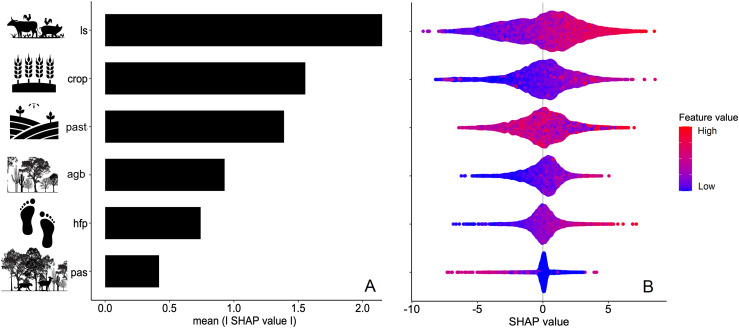
Drivers of medium- to large-bodied mammal assemblages defaunation in the Caatinga. A) Ranked feature importance values in terms of mean predictors of defaunation rates across the Caatinga region. B) Ranked feature importance values of predictors of defaunation for both negative (left) and (right) positive trends. Predictors are coded as follows: Livestock (ls), Crop (crop), Pasture (past), Aboveground Biomass (agb), Human footprint (hfp), and Protected Areas (pas).

GBM results, including the training and testing data, yielded an R² value of 0.82 (RMSE_training_ = 6.02; RMSE_testing_ = 3.82). Livestock density, cropland area, and pasture area were the main predictors of the magnitude of defaunation across the Caatinga, representing the highest relative importance values, as measured by SHAP (SHapley Additive exPlanations), compared to the human footprint, aboveground vegetation biomass, and protected area coverage (Fig 6). Therefore, acute disturbances associated with historical to modern agricultural land use were the main drivers of defaunation across the Caatinga. On the other hand, protected area cover was the only covariate inhibiting defaunation, indicating the key importance of protection status in retaining large mammals across the region (Fig 6). Contrary to expectations, aboveground biomass was in fact *positively* associated with defaunation. This indicates that, should a site lack strict protection, this key component of the mammal fauna can be lost even under a favorable scenario where suitable dry forest habitats are available, not least because those dry forest areas may be preferred by hunters and continue to be overhunted.

## Discussion

Our results clearly show a drastic level of defaunation across the entire Caatinga semi-arid region, 90% of which has lost 20–84% of its mid-sized to large-bodied mammals. Extirpation levels exceeded 50% (at least 37 species) in 71% of the entire region. This pervasive state of defaunation clearly shows that modern mammal assemblages in Earth’s most species-rich dry forest realm are both very depauperate and now composed primarily of small-bodied species. This pattern of large vertebrate species loss further resulted in loss of mammal functional diversity. The relentless conversion over historical and contemporary times of natural habitats into low-yield pasture areas for livestock and croplands has been a significant driver of defaunation, highlighting the all-important effect of habitat loss on Neotropical mammal faunas [46]. On the other hand, the still limited allocation of remaining Caatinga wildlands to fully protected areas underscore the importance of retaining non-hunted suitable habitats for large mammals. Only 9.1% of the entire Caatinga region is currently protected, and this includes multiple use indigenous territories, making it the second least protected in Brazil. However, aboveground vegetation biomass may indicate high quality of habitat but was surprisingly positively associated with defaunation, perhaps because most high-stature dry forest canopy areas are in the vicinities of densely settled areas, where hunting pressure is likely higher. This scenario exacerbates the dire need for rewilding efforts in one of the most threatened ecosystems of the Americas [47], with implications to essential ecosystem functions.

Bogoni et al. (2020) estimated that the average local species richness of MLBMs across the entire Neotropics declined from 32 to 12 species from historical to modern times, yielding an average loss of approximately 63% of the local species pool per site. Our results are similar in showing that, on average, species richness decreased from 33 to 13 species within the Caatinga region alone, resulting in a mean species loss of 57%. However, rates of local species loss were as high as ~80% and 90%, and the entire Caatinga region exhibited evidence of severe defaunation, which supports the notion that this is the most defaunated ecoregion across the New World tropics [13]. Therefore, the mammals in most of the Caatinga are currently only a pale shadow of its former glory, even if we ignore much older pre-Columbian extinctions began well before the arrival of Clovis cultures [48].

Downsizing of species assemblages has been widely observed in partially defaunated tropical regions elsewhere [10,15]. This has occurred over multiple waves of extinction through time since the colonization and spread of Clovis hunters throughout the Americas some 12,000 BP [49] with profound implications to ecosystem functioning [50]. Likewise, our results also show that the aggregate body mass of MLBM assemblages in historical times was on average four times greater than those at present. Very large-bodied species (>30 kg), such as giant armadillo, capybara, white-lipped peccary and lowland tapir, were locally extinct in over 80% of their geographic distribution areas across the entire region. Pervasive overhunting since colonial times is likely a decisive driver of local extinctions of large-bodied game species, and a strong contributor to overall assemblage downsizing [10,51]. However, hunting continues to be widespread across the Caatinga, where even small-bodied game smaller than 300g are typically harvested [21]. The Caatinga semi-arid region is characterized by high human population density, widespread rural poverty, scarcity of alternative animal protein, frequent malnutrition, and low human development index (HDI) [52]. all of which contribute to the heavy reliance on subsistence hunting that still subsidizes household economies. Additionally, many mammals are harvested for other purposes, such as religious rituals (collared anteater), house pets (titi monkeys, white-tufted-tar marmoset), medicinal reasons (*Herpailurus yagouaroundi, Cerdocyon thous*), and retaliation against perceived livestock depredation (*Puma concolor*) [53–55].

The loss of large herbivores and frugivores erodes mammal functional roles in nutrient cycling, carbon storage, seed dispersal and plant regeneration [56]. At a regional scale, changes in the size structure of vertebrate communities affect mammal functional diversity across Neotropical ecosystems [46]. In the world’s arid lands, loss of functional diversity has been mainly attributed to chronic human disturbance, which can result in undesirable degraded lands, and ultimately, desertification [57]. Defaunation of the Caatinga further aggravates the decay in functional diversity that is already affected by other anthropogenic stressors. Therefore, extirpation of the large-bodied mammals further intensifies a wide spectrum of human drivers in a region that retains only 11% of the landscape remains as undisturbed original habitat [22].

Most large-bodied mammals are wide ranging and have large spatial requirements [58,59], yet habitat loss reduces local population sizes [60–62], often to the point of extirpation [56]. In dry tropical regions, habitat loss is mainly associated with large-scale agriculture, not least because growing food demand can be met by soils that are sufficiently fertile. Approximately 60% of the Caatinga is allocated to bovine cattle and goat livestock [63]. Livestock has been prominent in the reginal economy since the 18th century, and beef production has sharply increased because of growing urban demand and the establishment of modern slaughterhouses [64]. Yet intensification and expansion of the rural livestock sector present several conservation challenges including habitat loss, pasture renovation, competition for grazelands, and transmission of infectious diseases to native mammals, all of which can further accelerate the defaunation process [1]. This scenario is a portrait of contemporary Caatinga environments, which are dominated by landscapes historically characterized by extensive, low-yield pastoral activities where only two species — exotic goats and native crab-eating fox — account for 60% of all mammals detected in camera-trapping surveys [65].

One way to mitigate landscape changes and interactions between domesticated and native fauna is the creation of additional (and expansion of existing) protected areas across the region. Only 1.92% of the Caatinga is set aside as state-managed protected areas, which clearly falls very short of the minimum Brazil-ratified target of 17%, according to the Convention on Biological Diversity [66]. Another approach would be to distribute the financial resources imposed by the Ministry of the Environment (MMA), as they are considered ~13 times lower than the amounts indicated by the MMA itself as necessary for the basic functioning of a conservation unit in Brazil [67]. Furthermore, our results show that strictly protected areas play a critical role in mitigating defaunation across the Caatinga, whereas this effect was not observed in sustainable use reserves which retained paltry species pools comparable to areas that remain entirely unprotected. Yet even strictly protected reserves had lost some of their original mammals. For example, assemblages within the five largest and best- protected reserves have lost 5–9 of the 51 species considered here, most likely before those reserves were created in the 1950s (PN Ubajara), 1970s (PN Serra da Capivara), 1990s (PN Confusões), and 2000s (Reserva Natural Serra das Almas and PN Boqueirão das Onças). Although most protected areas in Brazil (known as ‘Conservation Units’) are designated for sustainable use, strictly protected areas in the Caatinga effectively prevent deforestation by 27%, whereas sustainable use reserves fail to do so [66]. Therefore, our findings endorse the need to create more and/or larger fully protected areas to effectively mitigate defaunation throughout the region.

Of the 51 species considered in this study, 37 lost more than 50% of their geographic range across the Caatinga. Two of these are regional endemic species and one is threatened with extinction: the blond titi monkey (*Callicebus barbarabrownae*), the Brazilian porcupine (*Coendou baturitensis*) [68]. In addition to being critically endangered, our estimates indicate that only <20% of the potential range of blond titi monkeys is currently occupied. The Brazilian porcupine was only recently described to occur within a very restricted area [58], but our estimates indicate that over 75% of its potential occurrence has been converted to agricultural land uses, highlighting the need for urgent conservation measures. Habitat loss has also restricted the endangered rock cavy to within less than 50% of its former potential occurrence. Although this 800g rodent is commonly sighted in the most protected rocky sites, it is widely harvested and an easy target for hunters elsewhere [23,69].

What few species still apparently thrive against all odds in most of the Caatinga can be regarded as ‘winners’, as they are better adapted to highly human-modified landscapes [70]. Examples of these species include (1) crab-eating fox (*Cerdocyon thous*), a generalist canid that is adapted to highly degraded environments [71,72], and (2) six-banded-armadillo (*Euphractus sexcinctus*), which is highly tolerant to land use change, not least because of its fossorial habit [73]. Even though these disturbance-tolerant species may be regarded as ‘winners’, they still lost much of their areas of occurrence, which is aggravated by frequent roadkill along highways [73] and elevated hunting pressure across the region [22,23].

Our results therefore show a drastic and still ongoing process of extirpation of MLBMs throughout the Caatinga, driven mainly by livestock pastures and cropland, which have been expanding in the region since the 16th century. Defaunation further affects the size structure of mammal assemblages by replacing large and medium-sized species with smaller counterparts. This affects the loss of critical ecological functions, such as landscaping habitat, dispersal of large-seeded plants, carbon storage and plant regeneration. Our findings are entirely consistent with the historical context of overhunting, the effects of reduced forest cover, increasing levels of habitat degradation, and future climate projections, all of which can continue to fuel local extinctions of large vertebrates. Recent estimates on the effects of climate change on Caatinga mammal assemblages indicate that small-bodied mammals will be most impacted and lose most of their suitable habitats [74]. However, these estimates are based on ecological niche modelling, such as inferred historical distributions, and therefore fail to consider realistic scenarios of modern defaunation. Mammal distributions under the impending prospects of extreme climate events, including exacerbated droughts, are unlikely to stabilize because local to regional scale extinctions are still ongoing, and will likely be aggravated by increasing land use intensification. We therefore highlight the importance of urgent rewilding practices across the Caatinga if restoring ecological functions and resilience under climate change are societal goals both in terms of conservation and sustainable development. Furthermore, we urge the implementation of refaunation initiatives in areas that still retain high dry forest biomass but have become ‘empty forests’ due to a long history of overhunting.

## Supporting information

S1 File Suplementary methods about Species Distribution Model (SDM) (dx.doi.org/10.17504/protocols.io.8epv5kd2dv1b/v1- Methods explaining the protocol about species distribution model (SDM) and following the normative in the Zurell, et al 2020. A standard protocol for reporting species distribution models. – Ecography doi: 10.1111/ecog.04960.
(PDF)

S1 Table List of medium- to large-bodied mammal species of the semi-arid Caatinga dry forest domain (https://doi.org/10.6084/m9.figshare.30490709). List of medium- to large-bodied mammal species of the semi-arid Caatinga dry forest domain based on a comprehensive literature review (see Methods), resulting in a compilation of 73 mammals assemblages distributed throughout the region. This data was using for construction method about interpolation for species occurring in Caatinga limit.
(DOCX)

S2 Table Protocol about species distribution model (SDM) and following the Zurell, et al 2020. (DOCX)

S3 Table Description and value of thresholds and UAC used to generate the potential species distribution models of mammal species. (DOCX)

S1 Fig Historically predicted models for each of the 51 medium- to large-bodied mammal species occurring in the Caatinga considered in this study. (TIF)

S2 Fig Historically predicted models for each of the 51 medium- to large-bodied mammal species occurring in the Caatinga considered in this study. (TIF)

S3 Fig Historically predicted models for each of the 51 medium- to large-bodied mammal species occurring in the Caatinga considered in this study. (TIF)

S4 Fig Historically predicted models for each of the 51 medium- to large-bodied mammal species occurring in the Caatinga considered in this study. (TIF)

S5 Fig Spatial prediction of historical distribution models for each of the 51 medium- to large-bodied mammal species occurring in the Caatinga domain and the aggregate overlay of all binary species maps. (TIF)
