# Supplementary material for: Reconstructing the degree of mammal defaunation throughout the Caatinga - the largest dry tropical forest region of South America
Source: PLoS One. 2025 Nov 24;20(11):e0336562. doi: 10.1371/journal.pone.0336562 (PMC12643294; doi:10.1371/journal.pone.0336562)
Supplement: S1 File — A standard protocol for reporting species distribution models. – Ecography doi: 10.1111/ecog.04960. (PDF) [file pone.0336562.s001.pdf]

Suplementary methods about Species Distribution Model (SDM)

Methods explaining the protocol about species model distribution (SDM) and following the normative in the Zurell, et al 2020. A standard protocol for reporting species distribution models. – Ecography doi: 10.1111/ecog.04960.

Species Distribution Modeling Approach

We used species distribution models (SDMs) to predict the potential distribution of 51 medium- to large-bodied mammal (MLBM) species known to occur in the Caatinga ecosystem, located in the northeastern region of Brazil. However, the SDMs were constructed across the entire South American continent. This broader approach was necessary to improve the accuracy and ecological relevance of the models by capturing the full range of climatic conditions in which each species could potentially occur. By incorporating presence records from across South America, we ensured that the models accounted for the complete climatic niche of each species, rather than being constrained to the specific conditions of the Caatinga. This allows for more robust predictions of species distribution by including the full range of environmental conditions in which they can thrive.

Species Occurrence Data Collection

Species occurrence data were gathered from various sources, including a bibliographic survey using the Google Scholar, SciELO, Web of Science, and ScienceDirect databases. We searched using keyword combinations in English and Portuguese, such as “medium- to large-bodied mammals,” “checklist,” “Caatinga,” “hunting,” and “ethnofauna,” to retrieve information on MLBM assemblages across the Caatinga. Additionally, we supplemented our dataset with spatially explicit occurrence records from the Global Biodiversity Information Facility (GBIF – https://www.gbif.org) and SpeciesLink (https://specieslink.net). In total, we compiled 8,169 occurrence records for 51 species across South America (all species are listed in the Supplementary Information in Table S1 and DOI:10.17632/bdmzzytkdm.1).

We compiled all data into a single table and used QGIS (Madeira version 4.4.14) to verify the geographic coordinates of each species occurrence. To ensure data accuracy, we checked for inconsistencies, confirmed that all occurrence points fell within the South American boundaries, and excluded any records outside this region. Additionally, we removed duplicate localities and filtered out records located within 2 km of each other for the same species.

Environmental Predictors and Data Processing

SDMs are powerful tools for ecological and biogeographical studies, enabling researchers to estimate potential species distributions based on environmental predictors. These models assume that species distributions are primarily influenced by abiotic factors such as climate and topography, which determine habitat suitability. This is especially important for mammal species, as climate and elevation are key drivers of their distribution patterns, affecting habitat selection, resource availability, and their overall ecological niche.

To model species distributions, we used climatic and topographic predictors from WorldClim version 2.1 (www.worldclim.org), with a spatial resolution of 2.5 arcminutes (≈ 4,630 m). WorldClim provides high-resolution interpolated climate data, derived from 9,000 to 60,000 weather stations worldwide, spanning a temporal range of 1970–2000. The dataset was generated using thin-plate spline interpolation, incorporating variables such as elevation, distance to the coast, and satellite-derived data. WorldClim also applies regionalized modeling, selecting the best-performing model for each region and variable, ensuring high accuracy (Fick and Hijmand, 2017). This approach provides robust, globally consistent climate estimates, making it ideal for species distribution modeling. The predictor variables were cropped to the rectangular region defined by the following coordinates: Longitude: -81.2083° W to -56.7500° W, and Latitude: -32.5000° S to 16.2917° N, which encompasses the entire South American continent.

To eliminate redundancy, we selected variables for the models that were correlated with each other at less than 0.8, based on a Pearson correlation matrix calculated using the R package "vegan" (version 3.5.3). As a result, we included the following 10 variables in the analysis: diurnal amplitude of mean temperature (BIO2), temperature seasonality (BIO4), mean temperature during the wettest (BIO8) and driest (BIO9) quarters, annual precipitation (BIO12), precipitation seasonality (BIO15), and precipitation during the driest (BIO17), warmest (BIO18), and coldest (BIO19) quarters, as well as terrain elevation.

Species Distribution Modeling with MaxEnt

We used the Maximum Entropy v.3.4.1 (MaxEnt) algorithm (Elith et al., 2011) to generate the modeled geographic distributions of each selected species. MaxEnt models comprise a probability distribution in which each grid cell predicts the suitability of conditions for each species (Elith et al., 2011). MaxEnt is a widely tested and robust algorithm for modeling species distributions, particularly when occurrence data is limited to presence-only records. Its ability to estimate probability distributions based on environmental constraints makes it well-suited for our dataset, ensuring reliable predictions even with sparse or incomplete occurrence data. Additionally, MaxEnt's regularization techniques help prevent overfitting, making it an appropriate choice for capturing the ecological niches of the studied species while maintaining model generalizability.

The model was implemented using the **maxent() function**, with the following parameters:

```
model <- maxent(predictors_current, sp.occ, args=c("-J","-P","randomtestpoints=25", "replicates=10","replicatetype=bootstrap", "randomseed"), path='Results')

model.rep_current <- predict(model,predictors_current)

model.mean_current <- mean(model.rep_current)

writeRaster(model.mean_current,filename='Results/current_rep.img',overwrite=TRUE,format="HFA")
```

predictors_current: The current climatic and topographic predictor variables used to model the species distribution (temporal range of 1970–2000; cropped to the rectangular region: Longitude: -81.2083° W to -56.7500° W, and Latitude: -32.5000° S to 16.2917° N).

sp.occ: The species occurrence data collected.

args: A set of additional arguments passed to the MaxEnt model:

-J: This option activates the Jackknife technique, which is used to assess the contribution of each predictor variable to the model's performance. It runs the model multiple times, each time excluding one predictor variable, to evaluate the effect of each variable on the model's accuracy. This helps in understanding the relative importance of each variable in predicting species distribution.

-P: This option instructs MaxEnt to fill in missing values (i.e., raster cells without data) with zeros. This ensures that missing data does not affect the modeling process and helps maintain the integrity and completeness of the analysis.

randomtestpoints=25: Defines the number of random test points to be used for model evaluation.

replicates=10: Specifies the number of bootstrap replicates used for model validation.

replicatetype=bootstrap: Indicates that bootstrap sampling is used to create replicates.

randomseed: Ensures the model's reproducibility by setting a fixed random seed for consistent results.

Next, the **predict() function** was applied to the MaxEnt model (model) using the predictors_current variables to generate species distribution predictions. The resulting predictions were stored in the object model.rep_current.

The mean of the predictions across the 10 bootstrap replicates was calculated and stored in model.mean_current.

The averaged prediction (model.mean_current) was then saved as a raster file (current_rep.img) in the specified format (HFA), with the option to overwrite any existing file.

Model Evaluation

Model performance was evaluated using the area under the curve (AUC) of the receiver operating characteristic (ROC), which ensures that the model is making accurate and reliable predictions. The model was run with 10 replicates, each with a maximum of 10,000 iterations. For each replicate, 10% of the average replicates were randomized as test data, while the remaining 90% were used for training. This approach ensures a robust evaluation of model performance.

Thresholding and Post-processing

Each species' predictive map was converted into a binary presence-absence map (0 = absence, 1 = presence) based on the threshold values determined by MaxEnt (Table S2). To minimize the risk of overestimating species' geographic distributions, we adopted a conservative approach, selecting the thresholds that identified the smallest possible suitable habitat. This ensured that the predicted distributions remained ecologically realistic and aligned with known habitat constraints.

Finally, all binary maps were clipped to the boundaries of the Caatinga ecosystem.

Software and Packages Used

We conducted data processing and species distribution modeling using the **R programming environment and the Maximum Entropy (MaxEnt) v.3.4.1 software**. Specifically, we employed the following R packages:

"vegan" (version 3.5.3) for ecological and statistical analyses, including the Pearson correlation matrix used to assess collinearity among predictor variables, ensuring that only weakly correlated environmental variables were included in the models.

"dismo" to interface with MaxEnt 3.4.1 and facilitate species distribution modeling.

"maptools" and **"rgdal"** for spatial data manipulation and analysis.

"rJava" to enable the execution of MaxEnt within R, as the software requires Java.

"raster" for handling raster datasets, including environmental predictors and model output maps.

These tools ensured a comprehensive workflow, from data preparation and model execution to spatial analysis and visualization of species distribution patterns.

We also used **QGIS (Madeira version 4.4.14)** for verifying the geographic coordinates of species occurrence data, ensuring that all occurrence points were within the boundaries of South America and detecting potential inconsistencies. Additionally, QGIS was employed for raster clipping operations.

Critical Model Assumptions:

1. The model assumes that species distributions reflect environmental suitability in the absence of human impact. Anthropogenic factors such as habitat destruction, land-use change, or hunting are not considered, meaning the predicted distribution represents where species could occur under natural, pre-disturbance conditions (“historical distribution”).

2. Species fill their niche and do not occur elsewhere.

3. Species occurrence data is adequate and representative. Any sampling biases are accounted for/corrected.

4. Independence of species observations. Each species occurrence record is treated as independent information, meaning it does not represent the same individual being reported multiple times.

5. Relevant ecological drivers (or proxies) of species distributions are included. The selected climatic and altitude variables are the primary drivers of the species distributions and adequately represent the ecological factors influencing their presence.

6. Predictors are free of error: the model assumes that the selected climatic and altitude variables from WorldClim 2.1 are accurately measured or estimated, with minimal errors that could affect the predictions.
