# Supplementary material for: Reconstructing the degree of mammal defaunation throughout the Caatinga - the largest dry tropical forest region of South America
Source: PLoS One. 2025 Nov 24;20(11):e0336562. doi: 10.1371/journal.pone.0336562 (PMC12643294; doi:10.1371/journal.pone.0336562)
Supplement: S1 Table — List of medium- to large-bodied mammal species of the semi-arid Caatinga dry forest domain based on a comprehensive literature review (see Methods), resulting in a compilation of 73 mammals assemblages distributed throughout the region. This data was using for construction method about interpolation for species occurring in Caatinga limit. (DOCX) [file pone.0336562.s002.docx]

**Table S1**. List of medium- to large-bodied mammal species of the semi-arid Caatinga dry forest domain based on a comprehensive literature review (see Methods), resulting in a compilation of 73 mammals assemblages distributed throughout the region. This data was using for construction method about interpolation for species occurring in Caatinga limit (https://doi.org/10.6084/m9.figshare.30490709).

| **Species** | **Popular name** | **Locality** | **State** | **Longitude** | **Latitude** | **Reference** | | **Methods of reference** | |
| --- | --- | --- | --- | --- | --- | --- | --- | --- | --- |
| *Cavia aperea* | Brazilian guinea pig | Agreste | Paraiba | -35.8435450000 | -7.35540300000 | Barbosa et al 2018 | Interview - hunter | |  |
| *Cerdocyon thous* | Crab eating fox | Agreste | Paraiba | -35.8435450000 | -7.35540300000 | Barbosa et al 2018 | Interview - hunter | |  |
| *Conepatus semistriatus* | Striped hog nosed Skunk | Agreste | Paraiba | -35.8435450000 | -7.35540300000 | Barbosa et al 2018 | Interview - hunter | |  |
| *Dasypus novemcinctus* | Nine-banded Armadillo | Agreste | Paraiba | -35.8435450000 | -7.35540300000 | Barbosa et al 2018 | Interview - hunter | |  |
| *Didelphis albiventris* | Common Opossum | Agreste | Paraiba | -35.8435450000 | -7.35540300000 | Barbosa et al 2018 | Interview - hunter | |  |
| *Euphractus sexcinctus* | Yellow Armadillo | Agreste | Paraiba | -35.8435450000 | -7.35540300000 | Barbosa et al 2018 | Interview - hunter | |  |
| *Kerodon rupestris* | Rock Cavy | Agreste | Paraiba | -35.8435450000 | -7.35540300000 | Barbosa et al 2018 | Interview - hunter | |  |
| *Subulo gouazoubira* | Gray Brocket | Agreste | Paraiba | -35.8435450000 | -7.35540300000 | Barbosa et al 2018 | Interview - hunter | |  |
| *Cerdocyon thous* | Crab eating fox | Bodoco | Pernambuco | -39.8267710000 | -7.73795000000 | Feijo et al 2013 | Museum collection | |  |
| *Conepatus semistriatus* | Striped hog nosed Skunk | Bodoco | Pernambuco | -39.8267710000 | -7.73795000000 | Feijo et al 2013 | Museum collection | |  |
| *Dasyprocta prymnolopha* | red-orange rump agoutis | Bodoco | Pernambuco | -39.8267710000 | -7.73795000000 | Feijo et al 2013 | Museum collection | |  |
| *Dasypus novemcinctus* | Nine-banded Armadillo | Bodoco | Pernambuco | -39.8267710000 | -7.73795000000 | Oliveira et al 2003 | Literature - checklist | |  |
| *Dasypus septemcinctus* | Brazilian Lesser long-nosed Armadillo | Bodoco | Pernambuco | -39.8267710000 | -7.73795000000 | Oliveira et al 2003 | Literature - checklist | |  |
| *Didelphis albiventris* | White-eared Opossum | Bodoco | Pernambuco | -39.8267710000 | -7.73795000000 | Oliveira et al 2003 | Literature - checklist | |  |
| *Euphractus sexcinctus* | Yellow Armadillo | Bodoco | Pernambuco | -39.8267710000 | -7.73795000000 | Oliveira et al 2003 | Literature - checklist | |  |
| *Galea spixii* | Spix's Yellow-toothed Cavy | Bodoco | Pernambuco | -39.8267710000 | -7.73795000000 | Oliveira et al 2003 | Literature - checklist | |  |
| *Galictis cuja* | Lesser Grison | Bodoco | Pernambuco | -39.8267710000 | -7.73795000000 | Feijo et al 2013 | Museum collection | |  |
| *Herpailurus yagouaroundi* | Jaguarundi | Bodoco | Pernambuco | -39.8267710000 | -7.73795000000 | Feijo et al 2013 | Museum collection | |  |
| *Kerodon rupestris* | Rock Cavy | Bodoco | Pernambuco | -39.8267710000 | -7.73795000000 | Oliveira et al 2003 | Literature - checklist | |  |
| *Subulo gouazoubira* | Gray Brocket | Bodoco | Pernambuco | -39.8267710000 | -7.73795000000 | Feijo et al 2013 | Museum collection | |  |
| *Tolypeutes tricinctus* | Brazilian Three banded Armadillo | Bodoco | Pernambuco | -39.8267710000 | -7.73795000000 | Oliveira et al 2003 | Literature - checklist | |  |
| *Alouatta caraya* | Black-and-gold Howler monkey | Boqueirão das onças | Bahia | -41.869 | -9.777 | Campos et al 2019 | Camera trappingtrappingping, indirect evidence -checklist | |  |
| *Callithrix jacchus* | Common marmoset | Boqueirão das onças | Bahia | -41.869 | -9.777 | Campos et al 2019 | Camera trappingtrappingping, indirect evidence -checklist | |  |
| *Cerdocyon thous* | Crab eating fox | Boqueirão das onças | Bahia | -41.869 | -9.777 | Campos et al 2019 | Camera trappingtrappingping, indirect evidence -checklist | |  |
| *Conepatus semistriatus* | Striped hog nosed Skunk | Boqueirão das onças | Bahia | -41.869 | -9.777 | Campos et al 2019 | Camera trappingtrappingping, indirect evidence -checklist | |  |
| *Dasyprocta prymnolopha* | red-orange rump agoutis | Boqueirão das onças | Bahia | -41.869 | -9.777 | Campos et al 2019 | Camera trappingtrappingping, indirect evidence -checklist | |  |
| *Dasypus novemcinctus* | Nine-banded Armadillo | Boqueirão das onças | Bahia | -41.869 | -9.777 | Campos et al 2019 | Camera trappingtrappingping, indirect evidence -checklist | |  |
| *Eira barbara* | Tayra | Boqueirão das onças | Bahia | -41.869 | -9.777 | Campos et al 2019 | Camera trappingtrappingping, indirect evidence -checklist | |  |
| *Euphractus sexcinctus* | Yellow Armadillo | Boqueirão das onças | Bahia | -41.869 | -9.777 | Campos et al 2019 | Camera trappingtrappingping, indirect evidence -checklist | |  |
| *Galea spixii* | Spix's Yellow-toothed Cavy | Boqueirão das onças | Bahia | -41.869 | -9.777 | Campos et al 2019 | Camera trappingtrappingping, indirect evidence -checklist | |  |
| *Galictis cuja* | Lesser Grison | Boqueirão das onças | Bahia | -41.869 | -9.777 | Campos et al 2019 | Camera trapping, indirect evidence -checklist | |  |
| *Herpailurus yagouaroundi* | Jaguarundi | Boqueirão das onças | Bahia | -41.869 | -9.777 | Campos et al 2019 | Camera trapping, indirect evidence -checklist | |  |
| *Kerodon rupestris* | Rock Cavy | Boqueirão das onças | Bahia | -41.869 | -9.777 | Campos et al 2019 | Camera trapping, indirect evidence -checklist | |  |
| *Leopardus emiliae* | Tiger cat | Boqueirão das onças | Bahia | -41.869 | -9.777 | Campos et al 2019 | Camera trapping, indirect evidence -checklist | |  |
| *Leopardus pardalis* | Ocelot | Boqueirão das onças | Bahia | -41.869 | -9.777 | Campos et al 2019 | Camera trapping, indirect evidence -checklist | |  |
| *Subulo gouazoubira* | Gray Brocket | Boqueirão das onças | Bahia | -41.869 | -9.777 | Campos et al 2019 | Camera trapping, indirect evidence -checklist | |  |
| *Myrmecophaga tridactyla* | Giant Anteater | Boqueirão das onças | Bahia | -41.869 | -9.777 | Campos et al 2019 | Camera trapping, indirect evidence -checklist | |  |
| *Nasua nasua* | South American Coati | Boqueirão das onças | Bahia | -41.869 | -9.777 | Campos et al 2019 | Camera trapping, indirect evidence -checklist | |  |
| *Panthera onca* | Jaguar | Boqueirão das onças | Bahia | -41.869 | -9.777 | Campos et al 2019 | Camera trapping, indirect evidence -checklist | |  |
| *Dicotyles tajacu* | Collaredy Pecary | Boqueirão das onças | Bahia | -41.869 | -9.777 | Campos et al 2019 | Camera trapping, indirect evidence -checklist | |  |
| *Procyon cancrivorus* | Crab eating Raccoon | Boqueirão das onças | Bahia | -41.869 | -9.777 | Campos et al 2019 | Camera trapping, indirect evidence -checklist | |  |
| *Puma concolor* | Puma | Boqueirão das onças | Bahia | -41.869 | -9.777 | Campos et al 2019 | Camera trapping, indirect evidence -checklist | |  |
| *Tamandua tetradactyla* | Southern Tamandua | Boqueirão das onças | Bahia | -41.869 | -9.777 | Campos et al 2019 | Camera trapping, indirect evidence -checklist | |  |
| *Tayassu pecari* | White-lipped Peccary | Boqueirão das onças | Bahia | -41.869 | -9.777 | Campos et al 2019 | Camera trapping, indirect evidence -checklist | |  |
| *Tolypeutes tricinctus* | Brazilian Three banded Armadillo | Boqueirão das onças | Bahia | -41.869 | -9.777 | Campos et al 2019 | Camera trapping, indirect evidence -checklist | |  |
| *Callithrix jacchus* | Common marmoset | Cabaceiras | Paraiba | -36.3523550000 | -7.41399500000 | Gbif | Observation | |  |
| *Didelphis albiventris* | White-eared Opossum | Cabaceiras | Paraiba | -36.3523550000 | -7.41399500000 | Gbif | Observation | |  |
| *Galea spixii* | Spix's Yellow-toothed Cavy | Cabaceiras | Paraiba | -36.3523550000 | -7.41399500000 | Gbif | Observation | |  |
| *Kerodon rupestris* | Rock Cavy | Cabaceiras | Paraiba | -36.3523550000 | -7.41399500000 | Gbif | Observation | |  |
| *Procyon cancrivorus* | Crab eating Raccoon | Cabaceiras | Paraiba | -36.3523550000 | -7.41399500000 | Gbif | Observation | |  |
| *Cerdocyon thous* | Crab eating fox | Caiçara do Norte | Rio Grande do Norte | -36.0389610000 | -5.19948900000 | Marinho et al 2018 | Camera trapping - checklist | |  |
| *Conepatus semistriatus* | Striped hog nosed Skunk | Caiçara do Norte | Rio Grande do Norte | -36.0389610000 | -5.19948900000 | Marinho et al 2018 | Camera trapping - checklist | |  |
| *Didelphis albiventris* | White-eared Opossum | Caiçara do Norte | Rio Grande do Norte | -36.0389610000 | -5.19948900000 | Marinho et al 2018 | Camera trapping - checklist | |  |
| *Euphractus sexcinctus* | Yellow Armadillo | Caiçara do Norte | Rio Grande do Norte | -36.0389610000 | -5.19948900000 | Marinho et al 2018 | Camera trapping - checklist | |  |
| *Leopardus emiliae* | Tiger cat | Caiçara do Norte | Rio Grande do Norte | -36.0389610000 | -5.19948900000 | Marinho et al 2018 | Camera trapping - checklist | |  |
| *Subulo gouazoubira* | Gray Brocket | Caiçara do Norte | Rio Grande do Norte | -36.0389610000 | -5.19948900000 | Marinho et al 2018 | Camera trapping - checklist | |  |
| *Cavia aperea* | Brazilian guinea pig | Caico | Rio Grande do Norte | -37.1053430000 | -6.45528100000 | Barbosa et al 2016 | Interview - hunter | |  |
| *Cerdocyon thous* | Crab eating fox | Caico | Rio Grande do Norte | -37.1053430000 | -6.45528100000 | Barbosa et al 2016 | Interview - hunter | |  |
| *Conepatus semistriatus* | Striped hog nosed Skunk | Caico | Rio Grande do Norte | -37.1053430000 | -6.45528100000 | Barbosa et al 2016 | Interview - hunter | |  |
| *Dasyprocta prymnolopha* | red-orange rump agoutis | Caico | Rio Grande do Norte | -37.1053430000 | -6.45528100000 | Barbosa et al 2016 | Interview - hunter | |  |
| *Dasypus novemcinctus* | Nine-banded Armadillo | Caico | Rio Grande do Norte | -37.1053430000 | -6.45528100000 | Barbosa et al 2016 | Interview - hunter | |  |
| *Didelphis marsupialis* | Common Opossum | Caico | Rio Grande do Norte | -37.1053430000 | -6.45528100000 | Barbosa et al 2016 | Interview - hunter | |  |
| *Euphractus sexcinctus* | Yellow Armadillo | Caico | Rio Grande do Norte | -37.1053430000 | -6.45528100000 | Barbosa et al 2016 | Interview - hunter | |  |
| *Galea spixii* | Spix's Yellow-toothed Cavy | Caico | Rio Grande do Norte | -37.1053430000 | -6.45528100000 | Barbosa et al 2016 | Interview - hunter | |  |
| *Galictis vittata* | Greater Grison | Caico | Rio Grande do Norte | -37.1053430000 | -6.45528100000 | Barbosa et al 2016 | Interview - hunter | |  |
| *Herpailurus yagouaroundi* | Jaguarundi | Caico | Rio Grande do Norte | -37.1053430000 | -6.45528100000 | Barbosa et al 2016 | Interview - hunter | |  |
| *Kerodon rupestris* | Rock Cavy | Caico | Rio Grande do Norte | -37.1053430000 | -6.45528100000 | Barbosa et al 2016 | Interview - hunter | |  |
| *Leopardus emiliae* | Tiger cat | Caico | Rio Grande do Norte | -37.1053430000 | -6.45528100000 | Barbosa et al 2016 | Interview - hunter | |  |
| *Leopardus pardalis* | Ocelot | Caico | Rio Grande do Norte | -37.1053430000 | -6.45528100000 | Barbosa et al 2016 | Interview - hunter | |  |
| *Leopardus wiedii* | Margay | Caico | Rio Grande do Norte | -37.1053430000 | -6.45528100000 | Barbosa et al 2016 | Interview - hunter | |  |
| *Mazama americana* | Red Brocket | Caico | Rio Grande do Norte | -37.1053430000 | -6.45528100000 | Barbosa et al 2016 | Interview - hunter | |  |
| *Subulo gouazoubira* | Gray Brocket | Caico | Rio Grande do Norte | -37.1053430000 | -6.45528100000 | Barbosa et al 2016 | Interview - hunter | |  |
| *Procyon cancrivorus* | Crab eating Raccoon | Caico | Rio Grande do Norte | -37.1053430000 | -6.45528100000 | Barbosa et al 2016 | Interview - hunter | |  |
| *Tamandua tetradactyla* | Southern Tamandua | Caico | Rio Grande do Norte | -37.1053430000 | -6.45528100000 | Barbosa et al 2016 | Interview - hunter | |  |
| *Callithrix jacchus* | Common marmoset | Caninde do Sao Francisco | Sergipe | -37.8750560000 | -9.73983300000 | Gbif | Observation | |  |
| *Cerdocyon thous* | Crab eating fox | Caninde do Sao Francisco | Sergipe | -37.8750560000 | -9.73983300000 | Bezerra et al 2014 | Direct evidence, trails - checklist | |  |
| *Galea spixii* | Spix's Yellow-toothed Cavy | Caninde do Sao Francisco | Sergipe | -37.8750560000 | -9.73983300000 | Bezerra et al 2014 | Direct evidence, trails - checklist | |  |
| *Kerodon rupestris* | Rock Cavy | Caninde do Sao Francisco | Sergipe | -37.8750560000 | -9.73983300000 | Gbif | Observation | |  |
| *Procyon cancrivorus* | Crab eating Raccoon | Caninde do Sao Francisco | Sergipe | -37.8750560000 | -9.73983300000 | Bezerra et al 2014 | Direct evidence, trails - checklist | |  |
| *Bradypus variegatus* | Brown-throated Sloth | Caruaru | Pernambuco | -35.9213890000 | -8.23861100000 | Alves et al 2009B | Interview - medicinal use | |  |
| *Coendou prehensilis* | Brazilian Porcupine | Caruaru | Pernambuco | -35.9213890000 | -8.23861100000 | Alves et al 2009B | Interview - medicinal use | |  |
| *Conepatus semistriatus* | Striped hog nosed Skunk | Caruaru | Pernambuco | -35.9213890000 | -8.23861100000 | Alves et al 2009B | Interview - medicinal use | |  |
| *Dasypus novemcinctus* | Nine-banded Armadillo | Caruaru | Pernambuco | -35.9213890000 | -8.23861100000 | Alves et al 2009B, Feijo et al 2009 | Interview - medicinal use | |  |
| *Didelphis albiventris* | White-eared Opossum | Caruaru | Pernambuco | -35.9213890000 | -8.23861100000 | Gbif | Observation | |  |
| *Euphractus sexcinctus* | Yellow Armadillo | Caruaru | Pernambuco | -35.9213890000 | -8.23861100000 | Alves et al 2009B | Interview - medicinal use | |  |
| *Cerdocyon thous* | Crab eating fox | Cerro Corá | Rio Grande do Norte | -36.3332560000 | -6.08777800000 | Marinho et al 2018 | Camera trapping - checklist | |  |
| *Didelphis albiventris* | White-eared Opossum | Cerro Corá | Rio Grande do Norte | -36.3332560000 | -6.08777800000 | Marinho et al 2018 | Camera trapping - checklist | |  |
| *Euphractus sexcinctus* | Yellow Armadillo | Cerro Corá | Rio Grande do Norte | -36.3332560000 | -6.08777800000 | Marinho et al 2018 | Camera trapping - checklist | |  |
| *Leopardus emiliae* | Tiger cat | Cerro Corá | Rio Grande do Norte | -36.3332560000 | -6.08777800000 | Marinho et al 2018 | Camera trapping - checklist | |  |
| *Leopardus pardalis* | Ocelot | Cerro Corá | Rio Grande do Norte | -36.3332560000 | -6.08777800000 | Marinho et al 2018 | Camera trapping - checklist | |  |
| *Subulo gouazoubira* | Gray Brocket | Cerro Corá | Rio Grande do Norte | -36.3332560000 | -6.08777800000 | Marinho et al 2018 | Camera trapping - checklist | |  |
| *Procyon cancrivorus* | Crab eating Raccoon | Cerro Corá | Rio Grande do Norte | -36.3332560000 | -6.08777800000 | Marinho et al 2018 | Camera trapping - checklist | |  |
| *Alouatta caraya* | Black-and-gold Howler monkey | Chapada diamantina | Bahia | -41.3289980000 | -12.95332000000 | Pereira & Gueise 2009 | Collection, interview - checklist | |  |
| *Cabassous tatouay* | Greater Naked-tailed Armadillo | Chapada diamantina | Bahia | -41.32899800000 | -12.95332000000 | Pereira & Gueise 2009 | Collection, interview - checklist | |  |
| *Callicebus barbarabrownae* | Blond titi monkey | Chapada diamantina | Bahia | -41.32899800000 | -12.95332000000 | Gbif | Collection | |  |
| *Callithrix jacchus* | Common marmoset | Chapada diamantina | Bahia | -41.32899800000 | -12.95332000000 | Gbif | Collection | |  |
| *Callithrix penicillata* | Black pencilled marmoset | Chapada diamantina | Bahia | -41.32899800000 | -12.95332000000 | Pereira & Gueise 2009 | Collection, interview - checklist | |  |
| *Cerdocyon thous* | Crab eating fox | Chapada diamantina | Bahia | -41.32899800000 | -12.95332000000 | Pereira & Gueise 2009 | Collection, interview - checklist | |  |
| *Cuniculus paca* | Agouti | Chapada diamantina | Bahia | -41.32899800000 | -12.95332000000 | Pereira & Gueise 2009 | Collection, interview - checklist | |  |
| *Dasyprocta prymnolopha* | red-orange rump agoutis | Chapada diamantina | Bahia | -41.32899800000 | -12.95332000000 | Pereira & Gueise 2009 | Collection, interview - checklist | |  |
| *Dasypus novemcinctus* | Nine-banded Armadillo | Chapada diamantina | Bahia | -41.32899800000 | -12.95332000000 | Pereira & Gueise 2009 | Collection, interview - checklist | |  |
| *Didelphis albiventris* | White-eared Opossum | Chapada diamantina | Bahia | -41.32899800000 | -12.95332000000 | Gbif | Collection | |  |
| *Eira barbara* | Tayra | Chapada diamantina | Bahia | -41.32899800000 | -12.95332000000 | Pereira & Gueise 2009 | Collection, interview - checklist | |  |
| *Euphractus sexcinctus* | Yellow Armadillo | Chapada diamantina | Bahia | -41.32899800000 | -12.95332000000 | Pereira & Gueise 2009 | Collection, interview - checklist | |  |
| *Galea spixii* | Spix's Yellow-toothed Cavy | Chapada diamantina | Bahia | -41.32899800000 | -12.95332000000 | Pereira & Gueise 2009 | Collection, interview - checklist | |  |
| *Herpailurus yagouaroundi* | Jaguarundi | Chapada diamantina | Bahia | -41.32899800000 | -12.95332000000 | Pereira & Gueise 2009 | Collection, interview - checklist | |  |
| *Hydrochoerus hydrochaeris* | Capybara | Chapada diamantina | Bahia | -41.32899800000 | -12.95332000000 | Pereira & Gueise 2009 | Collection, interview - checklist | |  |
| *Kerodon rupestris* | Rock Cavy | Chapada diamantina | Bahia | -41.32899800000 | -12.95332000000 | Gbif | Observation | |  |
| *Lycalopex vetulus* | Hoary fox | Chapada diamantina | Bahia | -41.32899800000 | -12.95332000000 | Pereira & Gueise 2009 | Collection, interview - checklist | |  |
| *Subulo gouazoubira* | Gray Brocket | Chapada diamantina | Bahia | -41.32899800000 | -12.95332000000 | Gbif | Collection | |  |
| *Myrmecophaga tridactyla* | Giant Anteater | Chapada diamantina | Bahia | -41.32899800000 | -12.95332000000 | Pereira & Gueise 2009 | Collection, interview - checklist | |  |
| *Nasua nasua* | South American Coati | Chapada diamantina | Bahia | -41.32899800000 | -12.95332000000 | Pereira & Gueise 2009 | Collection, interview - checklist | |  |
| *Panthera onca* | Jaguar | Chapada diamantina | Bahia | -41.32899800000 | -12.95332000000 | Pereira & Gueise 2009 | Collection, interview - checklist | |  |
| *Dicotyles tajacu* | Collaredy Pecary | Chapada diamantina | Bahia | -41.32899800000 | -12.95332000000 | Pereira & Gueise 2009 | Collection, interview - checklist | |  |
| *Procyon cancrivorus* | Crab eating Raccoon | Chapada diamantina | Bahia | -41.32899800000 | -12.95332000000 | Pereira & Gueise 2009 | Collection, interview - checklist | |  |
| *Puma concolor* | Puma | Chapada diamantina | Bahia | -41.32899800000 | -12.95332000000 | Pereira & Gueise 2009 | Collection, interview - checklist | |  |
| *Sapajus xanthosternos* | Buff headed Capuchin | Chapada diamantina | Bahia | -41.32899800000 | -12.95332000000 | Pereira & Gueise 2009 | Collection, interview - checklist | |  |
| *Sylvilagus brasiliensis* | Tapeti | Chapada diamantina | Bahia | -41.32899800000 | -12.95332000000 | Gbif | Observation | |  |
| *Tamandua tetradactyla* | Southern Tamandua | Chapada diamantina | Bahia | -41.32899800000 | -12.95332000000 | Pereira & Gueise 2009 | Collection, interview - checklist | |  |
| *Callithrix jacchus* | Common marmoset | Coronel Ezequiel | Rio Grande do Norte | -36.22520800000 | -6.34020800000 | Lima et al 2018 | Interview - hunter | |  |
| *Cerdocyon thous* | Crab eating fox | Coronel Ezequiel | Rio Grande do Norte | -36.22520800000 | -6.34020800000 | Marinho et al 2018 | Camera trapping - checklist | |  |
| *Conepatus semistriatus* | Striped hog nosed Skunk | Coronel Ezequiel | Rio Grande do Norte | -36.22520800000 | -6.34020800000 | Marinho et al 2018 | Camera trapping - checklist | |  |
| *Dasypus novemcinctus* | Nine-banded Armadillo | Coronel Ezequiel | Rio Grande do Norte | -36.22520800000 | -6.34020800000 | Marinho et al 2018 | Camera trapping - checklist | |  |
| *Didelphis albiventris* | White-eared Opossum | Coronel Ezequiel | Rio Grande do Norte | -36.22520800000 | -6.34020800000 | Marinho et al 2018 | Camera trapping - checklist | |  |
| *Euphractus sexcinctus* | Yellow Armadillo | Coronel Ezequiel | Rio Grande do Norte | -36.22520800000 | -6.34020800000 | Marinho et al 2018 | Camera trapping - checklist | |  |
| *Galea spixii* | Spix's Yellow-toothed Cavy | Coronel Ezequiel | Rio Grande do Norte | -36.22520800000 | -6.34020800000 | Lima et al 2018 | Interview - hunter | |  |
| *Galictis cuja* | Lesser Grison | Coronel Ezequiel | Rio Grande do Norte | -36.22520800000 | -6.34020800000 | Lima et al 2018 | Interview - hunter | |  |
| *Herpailurus yagouaroundi* | Jaguarundi | Coronel Ezequiel | Rio Grande do Norte | -36.22520800000 | -6.34020800000 | Marinho et al 2018 | Camera trapping - checklist | |  |
| *Kerodon rupestris* | Rock Cavy | Coronel Ezequiel | Rio Grande do Norte | -36.22520800000 | -6.34020800000 | Lima et al 2018 | Interview - hunter | |  |
| *Leopardus emiliae* | Tiger cat | Coronel Ezequiel | Rio Grande do Norte | -36.22520800000 | -6.34020800000 | Marinho et al 2018 | Camera trapping - checklist | |  |
| *Subulo gouazoubira* | Gray Brocket | Coronel Ezequiel | Rio Grande do Norte | -36.22520800000 | -6.34020800000 | Marinho et al 2018 | Camera trapping - checklist | |  |
| *Myrmecophaga tridactyla* | Giant Anteater | Coronel Ezequiel | Rio Grande do Norte | -36.22520800000 | -6.34020800000 | Lima et al 2018 | Interview - hunter | |  |
| *Procyon cancrivorus* | Crab eating Raccoon | Coronel Ezequiel | Rio Grande do Norte | -36.22520800000 | -6.34020800000 | Marinho et al 2018 | Camera trapping - checklist | |  |
| *Tamandua tetradactyla* | Southern Tamandua | Coronel Ezequiel | Rio Grande do Norte | -36.22520800000 | -6.34020800000 | Lima et al 2018 | Interview - hunter | |  |
| *Tolypeutes tricinctus* | Brazilian Three banded Armadillo | Coronel Ezequiel | Rio Grande do Norte | -36.22520800000 | -6.34020800000 | Lima et al 2018 | Interview - hunter | |  |
| *Callithrix jacchus* | Common marmoset | Craibas | Alagoas | -36.73960000000 | -9.67010000000 | Gbif | Observation | |  |
| *Cerdocyon thous* | Crab eating fox | Craibas | Alagoas | -36.73960000000 | -9.67010000000 | Gbif | Observation | |  |
| *Didelphis albiventris* | White-eared Opossum | Craibas | Alagoas | -36.73960000000 | -9.67010000000 | Gbif | Observation | |  |
| *Leopardus emiliae* | Tiger cat | Craibas | Alagoas | -36.73960000000 | -9.67010000000 | Gbif | Observation | |  |
| *Procyon cancrivorus* | Crab eating Raccoon | Craibas | Alagoas | -36.73960000000 | -9.67010000000 | Gbif | Observation | |  |
| *Sylvilagus brasiliensis* | Tapeti | Craibas | Alagoas | -36.73960000000 | -9.67010000000 | Gbif | Observation | |  |
| *Dasyprocta prymnolopha* | red-orange rump agoutis | Delmiro Gouveia | Alagoas | -37.99215800000 | -9.40563100000 | Oliveira et al 2003 | Literature - checklist | |  |
| *Dasypus novemcinctus* | Nine-banded Armadillo | Delmiro Gouveia | Alagoas | -37.99215800000 | -9.40563100000 | Oliveira et al 2003 | Literature - checklist | |  |
| *Euphractus sexcinctus* | Yellow Armadillo | Delmiro Gouveia | Alagoas | -37.99215800000 | -9.40563100000 | Oliveira et al 2003 | Literature - checklist | |  |
| *Kerodon rupestris* | Rock Cavy | Delmiro Gouveia | Alagoas | -37.99215800000 | -9.40563100000 | Oliveira et al 2003 | Literature - checklist | |  |
| *Dicotyles tajacu* | Collaredy Pecary | Delmiro Gouveia | Alagoas | -37.99215800000 | -9.40563100000 | Oliveira et al 2003 | Literature - checklist | |  |
| *Tamandua tetradactyla* | Southern Tamandua | Delmiro Gouveia | Alagoas | -37.99215800000 | -9.40563100000 | Oliveira et al 2003 | Literature - checklist | |  |
| *Cerdocyon thous* | Crab eating fox | Dunas do Rosado | Rio Grande do Norte | -36.91305800000 | -5.13916100000 | Marinho et al 2018 | Camera trapping - checklist | |  |
| *Conepatus semistriatus* | Striped hog nosed Skunk | Dunas do Rosado | Rio Grande do Norte | -36.91305800000 | -5.13916100000 | Marinho et al 2018 | Camera trapping - checklist | |  |
| *Didelphis albiventris* | White-eared Opossum | Dunas do Rosado | Rio Grande do Norte | -36.91305800000 | -5.13916100000 | Marinho et al 2018 | Camera trapping - checklist | |  |
| *Euphractus sexcinctus* | Yellow Armadillo | Dunas do Rosado | Rio Grande do Norte | -36.91305800000 | -5.13916100000 | Marinho et al 2018 | Camera trapping - checklist | |  |
| *Leopardus emiliae* | Tiger cat | Dunas do Rosado | Rio Grande do Norte | -36.91305800000 | -5.13916100000 | Marinho et al 2018 | Camera trapping - checklist | |  |
| *Subulo gouazoubira* | Gray Brocket | Dunas do Rosado | Rio Grande do Norte | -36.91305800000 | -5.13916100000 | Marinho et al 2018 | Camera trapping - checklist | |  |
| *Tamandua tetradactyla* | Southern Tamandua | Dunas do Rosado | Rio Grande do Norte | -36.91305800000 | -5.13916100000 | Marinho et al 2018 | Camera trapping - checklist | |  |
| *Callithrix jacchus* | Common marmoset | Estacao Ecologica de Aiuaba | Ceara | -40.21314200000 | -6.67883600000 | Feijo et al 2013 | Interview | |  |
| *Cerdocyon thous* | Crab eating fox | Estacao Ecologica de Aiuaba | Ceara | -40.21314200000 | -6.67883600000 | Feijo et al 2013 | Interview | |  |
| *Conepatus semistriatus* | Striped hog nosed Skunk | Estacao Ecologica de Aiuaba | Ceara | -40.21314200000 | -6.67883600000 | Feijo et al 2013 | Museum collection, Interview | |  |
| *Cuniculus paca* | Agouti | Estacao Ecologica de Aiuaba | Ceara | -40.21314200000 | -6.67883600000 | Feijo et al 2013 | Interview | |  |
| *Dasypus novemcinctus* | Nine-banded Armadillo | Estacao Ecologica de Aiuaba | Ceara | -40.21314200000 | -6.67883600000 | Feijo et al 2013 | Interview | |  |
| *Euphractus sexcinctus* | Yellow Armadillo | Estacao Ecologica de Aiuaba | Ceara | -40.21314200000 | -6.67883600000 | Feijo et al 2013 | Museum collection, Interview | |  |
| *Galictis cuja* | Lesser Grison | Estacao Ecologica de Aiuaba | Ceara | -40.21314200000 | -6.67883600000 | Feijo et al 2013 | Interview | |  |
| *Subulo gouazoubira* | Gray Brocket | Estacao Ecologica de Aiuaba | Ceara | -40.21314200000 | -6.67883600000 | Feijo et al 2013 | Interview | |  |
| *Cavia aperea* | Brazilian guinea pig | Estacao Ecologica Raso da Catarina | Bahia | -38.51272200000 | -9.65083300000 | Santos et al 2018 | Interview - hunter | |  |
| *Dasypus novemcinctus* | Nine-banded Armadillo | Estacao Ecologica Raso da Catarina | Bahia | -38.51272200000 | -9.65083300000 | Santos et al 2018 | Interview - hunter | |  |
| *Dasypus septemcinctus* | Brazilian Lesser long-nosed Armadillo | Estacao Ecologica Raso da Catarina | Bahia | -38.51272200000 | -9.65083300000 | Santos et al 2018 | Interview - hunter | |  |
| *Euphractus sexcinctus* | Yellow Armadillo | Estacao Ecologica Raso da Catarina | Bahia | -38.51272200000 | -9.65083300000 | Santos et al 2018 | Interview - hunter | |  |
| *Subulo gouazoubira* | Gray Brocket | Estacao Ecologica Raso da Catarina | Bahia | -38.51272200000 | -9.65083300000 | Santos et al 2018 | Interview - hunter | |  |
| *Tolypeutes tricinctus* | Brazilian Three banded Armadillo | Estacao Ecologica Raso da Catarina | Bahia | -38.51272200000 | -9.65083300000 | Santos et al 2018 | Interview - hunter | |  |
| *Callithrix jacchus* | Common marmoset | Exu | Pernambuco | -39.68251700000 | -7.48255600000 | Oliveira et al 2003 | Literature - checklist | |  |
| *Cerdocyon thous* | Crab eating fox | Exu | Pernambuco | -39.68251700000 | -7.48255600000 | Feijo et al 2013, Oliveira et al 2003 | Collection | |  |
| *Dasyprocta prymnolopha* | red-orange rump agoutis | Exu | Pernambuco | -39.68251700000 | -7.48255600000 | Feijo et al 2013, Oliveira et al 2003 | Collection | |  |
| *Dasypus novemcinctus* | Nine-banded Armadillo | Exu | Pernambuco | -39.68251700000 | -7.48255600000 | Feijo et al 2013, Oliveira et al 2003 | Collection | |  |
| *Didelphis albiventris* | White-eared Opossum | Exu | Pernambuco | -39.68251700000 | -7.48255600000 | Oliveira et al 2003 | Literature - checklist | |  |
| *Euphractus sexcinctus* | Yellow Armadillo | Exu | Pernambuco | -39.68251700000 | -7.48255600000 | Oliveira et al 2003 | Literature - checklist | |  |
| *Galea spixii* | Spix's Yellow-toothed Cavy | Exu | Pernambuco | -39.68251700000 | -7.48255600000 | Gbif | Collection | |  |
| *Galictis cuja* | Lesser Grison | Exu | Pernambuco | -39.68251700000 | -7.48255600000 | Feijo et al 2013, Oliveira et al 2003 | Collection | |  |
| *Herpailurus yagouaroundi* | Jaguarundi | Exu | Pernambuco | -39.68251700000 | -7.48255600000 | Oliveira et al 2003 | Literature - checklist | |  |
| *Kerodon rupestris* | Rock Cavy | Exu | Pernambuco | -39.68251700000 | -7.48255600000 | Gbif | Collection | |  |
| *Leopardus emiliae* | Tiger cat | Exu | Pernambuco | -39.68251700000 | -7.48255600000 | Feijo et al 2013 | Collection | |  |
| *Subulo gouazoubira* | Gray Brocket | Exu | Pernambuco | -39.68251700000 | -7.48255600000 | Feijo et al 2013 | Collection | |  |
| *Panthera onca* | Jaguar | Exu | Pernambuco | -39.68251700000 | -7.48255600000 | Oliveira et al 2003 | Literature - checklist | |  |
| *Sapajus apella* | Black capped Capuchin | Exu | Pernambuco | -39.68251700000 | -7.48255600000 | Oliveira et al 2003 | Literature - checklist | |  |
| *Sapajus libidinosus* | Bearded Capuchin | Exu | Pernambuco | -39.68251700000 | -7.48255600000 | Gbif | Collection | |  |
| *Tamandua tetradactyla* | Southern Tamandua | Exu | Pernambuco | -39.68251700000 | -7.48255600000 | Feijo et al 2013, Oliveira et al 2003 | Collection | |  |
| *Callithrix jacchus* | Common marmoset | Fazenda São Paulo | Paraiba | -37.080833 | -7.649167 | Alves tese 2020 | Camera trapping - checklist | |  |
| *Cerdocyon thous* | Crab eating fox | Fazenda São Paulo | Paraiba | -37.080833 | -7.649167 | Alves tese 2020 | Camera trapping - checklist | |  |
| *Conepatus semistriatus* | Striped hog nosed Skunk | Fazenda São Paulo | Paraiba | -37.080833 | -7.649167 | Alves tese 2020 | Camera trapping - checklist | |  |
| *Didelphis albiventris* | White-eared Opossum | Fazenda São Paulo | Paraiba | -37.080833 | -7.649167 | Alves tese 2020 | Camera trapping - checklist | |  |
| *Euphractus sexcinctus* | Yellow Armadillo | Fazenda São Paulo | Paraiba | -37.080833 | -7.649167 | Alves tese 2020 | Camera trapping - checklist | |  |
| *Galea spixii* | Spix's Yellow-toothed Cavy | Fazenda São Paulo | Paraiba | -37.080833 | -7.649167 | Alves tese 2020 | Camera trapping - checklist | |  |
| *Herpailurus yagouaroundi* | Jaguarundi | Fazenda São Paulo | Paraiba | -37.080833 | -7.649167 | Alves tese 2020 | Camera trapping - checklist | |  |
| *Tamandua tetradactyla* | Southern Tamandua | Fazenda São Paulo | Paraiba | -37.080833 | -7.649167 | Alves tese 2020 | Camera trapping - checklist | |  |
| *Callicebus barbarabrownae* | Blond titi monkey | Fazenda Sao Pedro | Sergipe | -37.40861100000 | -10.03722200000 | Freitas et al 2011 | Indirect evidence, direct evidence - checklist | |  |
| *Callithrix jacchus* | Common marmoset | Fazenda Sao Pedro | Sergipe | -37.40861100000 | -10.03722200000 | Freitas et al 2011 | Indirect evidence, direct evidence - checklist | |  |
| *Cerdocyon thous* | Crab eating fox | Fazenda Sao Pedro | Sergipe | -37.40861100000 | -10.03722200000 | Freitas et al 2011 | Indirect evidence, direct evidence - checklist | |  |
| *Conepatus semistriatus* | Striped hog nosed Skunk | Fazenda Sao Pedro | Sergipe | -37.40861100000 | -10.03722200000 | Freitas et al 2011 | Indirect evidence, direct evidence - checklist | |  |
| *Kerodon rupestris* | Rock Cavy | Fazenda Sao Pedro | Sergipe | -37.40861100000 | -10.03722200000 | Freitas et al 2011 | Indirect evidence, direct evidence - checklist | |  |
| *Procyon cancrivorus* | Crab eating Raccoon | Fazenda Sao Pedro | Sergipe | -37.40861100000 | -10.03722200000 | Freitas et al 2011 | Indirect evidence, direct evidence - checklist | |  |
| *Cerdocyon thous* | Crab eating fox | Felipe Guerra | Rio Grande do Norte | -37.68865800000 | -5.59068100000 | Marinho et al 2018 | Camera trapping - checklist | |  |
| *Conepatus semistriatus* | Striped hog nosed Skunk | Felipe Guerra | Rio Grande do Norte | -37.68865800000 | -5.59068100000 | Marinho et al 2018 | Camera trapping - checklist | |  |
| *Didelphis albiventris* | White-eared Opossum | Felipe Guerra | Rio Grande do Norte | -37.68865800000 | -5.59068100000 | Marinho et al 2018 | Camera trapping - checklist | |  |
| *Euphractus sexcinctus* | Yellow Armadillo | Felipe Guerra | Rio Grande do Norte | -37.68865800000 | -5.59068100000 | Marinho et al 2018 | Camera trapping - checklist | |  |
| *Herpailurus yagouaroundi* | Jaguarundi | Felipe Guerra | Rio Grande do Norte | -37.68865800000 | -5.59068100000 | Marinho et al 2018 | Camera trapping - checklist | |  |
| *Leopardus emiliae* | Tiger cat | Felipe Guerra | Rio Grande do Norte | -37.68865800000 | -5.59068100000 | Marinho et al 2018 | Camera trapping - checklist | |  |
| *Procyon cancrivorus* | Crab eating Raccoon | Felipe Guerra | Rio Grande do Norte | -37.68865800000 | -5.59068100000 | Marinho et al 2018 | Camera trapping - checklist | |  |
| *Sapajus libidinosus* | Bearded Capuchin | Felipe Guerra | Rio Grande do Norte | -37.68865800000 | -5.59068100000 | Marinho et al 2018 | Camera trapping - checklist | |  |
| *Tamandua tetradactyla* | Southern Tamandua | Felipe Guerra | Rio Grande do Norte | -37.68865800000 | -5.59068100000 | Marinho et al 2018 | Camera trapping - checklist | |  |
| *Callithrix jacchus* | Common marmoset | Flona de Assú | Rio Grande do Norte | -36.94700300000 | -5.58344900000 | Cherem et al 2019, Feijo et al 2013 | Indirect evidence, direct evidence, camera trapping - checklist | |  |
| *Cerdocyon thous* | Crab eating fox | Flona de Assú | Rio Grande do Norte | -36.94700300000 | -5.58344900000 | Cherem et al 2019 | Indirect evidence, direct evidence, camera trapping - checklist | |  |
| *Conepatus semistriatus* | Striped hog nosed Skunk | Flona de Assú | Rio Grande do Norte | -36.94700300000 | -5.58344900000 | Cherem et al 2019 | Camera trapping - checklist | |  |
| *Didelphis albiventris* | White-eared Opossum | Flona de Assú | Rio Grande do Norte | -36.94700300000 | -5.58344900000 | Cherem et al 2019 | Indirect evidence, direct evidence, camera trapping - checklist | |  |
| *Euphractus sexcinctus* | Yellow Armadillo | Flona de Assú | Rio Grande do Norte | -36.94700300000 | -5.58344900000 | Cherem et al 2019 | Indirect evidence, direct evidence, camera trapping - checklist | |  |
| *Galea spixii* | Spix's Yellow-toothed Cavy | Flona de Assú | Rio Grande do Norte | -36.94700300000 | -5.58344900000 | Cherem et al 2019 | Indirect evidence, direct evidence, camera trapping - checklist | |  |
| *Galictis cuja* | Lesser Grison | Flona de Assú | Rio Grande do Norte | -36.94700300000 | -5.58344900000 | Cherem et al 2019 | Indirect evidence, camera trapping - checklists | |  |
| *Herpailurus yagouaroundi* | Jaguarundi | Flona de Assú | Rio Grande do Norte | -36.94700300000 | -5.58344900000 | Cherem et al 2019 | Camera trapping - checklist | |  |
| *Leopardus emiliae* | Tiger cat | Flona de Assú | Rio Grande do Norte | -36.94700300000 | -5.58344900000 | Cherem et al 2019 | Direct evidence, camera trapping - checklist | |  |
| *Procyon cancrivorus* | Crab eating Raccoon | Flona de Assú | Rio Grande do Norte | -36.94700300000 | -5.58344900000 | Cherem et al 2019 | Indirect evidence, camera trapping - checklists | |  |
| *Tamandua tetradactyla* | Southern Tamandua | Flona de Assú | Rio Grande do Norte | -36.94700300000 | -5.58344900000 | Cherem et al 2019 | Indirect evidence - checklist | |  |
| *Cabassous tatouay* | Greater Naked-tailed Armadillo | Floresta Nacional do Araripe | Ceara | -39.39434400000 | -7.41822200000 | Melo et al 2014, Silva- Neto et al 2017, Bonif├ício et al 2016, Feijo et al 2013 | Interview - hunter, Museum collection | |  |
| *Callithrix jacchus* | Common marmoset | Floresta Nacional do Araripe | Ceara | -39.39434400000 | -7.41822200000 | Gbif | Observation | |  |
| *Cerdocyon thous* | Crab eating fox | Floresta Nacional do Araripe | Ceara | -39.39434400000 | -7.41822200000 | Melo et al 2014, Silva- Neto et al 2017, Bonif├ício et al 2016, Feijo et al 2013, Gbif | Interview - hunter, collection,observation | |  |
| *Conepatus semistriatus* | Striped hog nosed Skunk | Floresta Nacional do Araripe | Ceara | -39.39434400000 | -7.41822200000 | Melo et al 2014, Silva- Neto et al 2017, Bonif├ício et al 2016 | Interview - hunter | |  |
| *Dasyprocta prymnolopha* | red-orange rump agoutis | Floresta Nacional do Araripe | Ceara | -39.39434400000 | -7.41822200000 | Melo et al 2014, Silva- Neto et al 2017, Bonif├ício et al 2016, Feijo et al 2013 | Interview - hunter, collection | |  |
| *Dasypus novemcinctus* | Nine-banded Armadillo | Floresta Nacional do Araripe | Ceara | -39.39434400000 | -7.41822200000 | Melo et al 2014, Silva- Neto et al 2017, Bonif├ício et al 2016 | Interview - hunter | |  |
| *Didelphis albiventris* | White-eared Opossum | Floresta Nacional do Araripe | Ceara | -39.39434400000 | -7.41822200000 | Gbif, Species link | Observation, collection | |  |
| *Eira barbara* | Tayra | Floresta Nacional do Araripe | Ceara | -39.39434400000 | -7.41822200000 | Silva- Neto et al 2017 | Interview - hunter | |  |
| *Euphractus sexcinctus* | Yellow Armadillo | Floresta Nacional do Araripe | Ceara | -39.39434400000 | -7.41822200000 | Melo et al 2014, Silva- Neto et al 2017, Bonif├ício et al 2016, Oliveira et al 2003 | Interview - hunter,checklist | |  |
| *Galea spixii* | Spix's Yellow-toothed Cavy | Floresta Nacional do Araripe | Ceara | -39.39434400000 | -7.41822200000 | Melo et al 2014, Silva- Neto et al 2017, Bonif├ício et al 2016, Spcies link | Interview - hunter, collection | |  |
| *Galictis cuja* | Lesser Grison | Floresta Nacional do Araripe | Ceara | -39.39434400000 | -7.41822200000 | Feijo et al 2013 | Museum collection | |  |
| *Galictis vittata* | Greater Grison | Floresta Nacional do Araripe | Ceara | -39.39434400000 | -7.41822200000 | Silva- Neto et al 2017, Bonif├ício et al 2016 | Interview - hunter | |  |
| *Herpailurus yagouaroundi* | Jaguarundi | Floresta Nacional do Araripe | Ceara | -39.39434400000 | -7.41822200000 | Silva- Neto et al 2017, Feijo et al 2013 | Interview - hunter, collection | |  |
| *Kerodon rupestris* | Rock Cavy | Floresta Nacional do Araripe | Ceara | -39.39434400000 | -7.41822200000 | Melo et al 2014, Silva- Neto et al 2017 | Interview - hunter | |  |
| *Leopardus emiliae* | Tiger cat | Floresta Nacional do Araripe | Ceara | -39.39434400000 | -7.41822200000 | Melo et al 2014, Silva- Neto et al 2017, Bonif├ício et al 2016, Feijo et al 2013 | Interview - hunter, collection | |  |
| *Leopardus pardalis* | Ocelot | Floresta Nacional do Araripe | Ceara | -39.39434400000 | -7.41822200000 | Silva- Neto et al 2017 | Interview - hunter | |  |
| *Leopardus wiedii* | Margay | Floresta Nacional do Araripe | Ceara | -39.39434400000 | -7.41822200000 | Silva- Neto et al 2017, Bonif├ício et al 2016 | Interview - hunter | |  |
| *Subulo gouazoubira* | Gray Brocket | Floresta Nacional do Araripe | Ceara | -39.39434400000 | -7.41822200000 | Melo et al 2014, Silva- Neto et al 2017, Bonif├ício et al 2016,Oliveira et al 2003, Feijo et al 2013 | Interview - hunter,checklist, collection | |  |
| *Nasua nasua* | South American Coati | Floresta Nacional do Araripe | Ceara | -39.39434400000 | -7.41822200000 | Bonif├ício et al 2016 | Interview - hunter | |  |
| *Ozotoceros bezoarticus* | Pampas deer | Floresta Nacional do Araripe | Ceara | -39.39434400000 | -7.41822200000 | Silva- Neto et al 2017 | Interview - hunter | |  |
| *Panthera onca* | Jaguar | Floresta Nacional do Araripe | Ceara | -39.39434400000 | -7.41822200000 | Melo et al 2014, Silva- Neto et al 2017, Bonif├ício et al 2016 | Interview - hunter | |  |
| *Dicotyles tajacu* | Collaredy Pecary | Floresta Nacional do Araripe | Ceara | -39.39434400000 | -7.41822200000 | Feijo et al 2013 | Museum collection | |  |
| *Procyon cancrivorus* | Crab eating Raccoon | Floresta Nacional do Araripe | Ceara | -39.39434400000 | -7.41822200000 | Silva- Neto et al 2017, Bonif├ício et al 2016, Feijo et al 2013 | Interview - hunter, collection | |  |
| *Puma concolor* | Puma | Floresta Nacional do Araripe | Ceara | -39.39434400000 | -7.41822200000 | Melo et al 2014, Silva- Neto et al 2017, Bonif├ício et al 2016, Feijo et al 2013 | Interview - hunter, collection | |  |
| *Sylvilagus brasiliensis* | Tapeti | Floresta Nacional do Araripe | Ceara | -39.39434400000 | -7.41822200000 | Melo et al 2014 | Interview - hunter | |  |
| *Tamandua tetradactyla* | Southern Tamandua | Floresta Nacional do Araripe | Ceara | -39.39434400000 | -7.41822200000 | Melo et al 2014, Silva- Neto et al 2017, Bonif├ício et al 2016, Oliveira et al 2003 | Interview - hunter,checklist | |  |
| *Tayassu* *pecari* | White-lipped Peccary | Floresta Nacional do Araripe | Ceara | -39.39434400000 | -7.41822200000 | Melo et al 2014, Silva- Neto et al 2017 | Interview - hunter | |  |
| *Tolypeutes tricinctus* | Brazilian Three banded Armadillo | Floresta Nacional do Araripe | Ceara | -39.39434400000 | -7.41822200000 | Melo et al 2014, Silva- Neto et al 2017 | Interview - hunter | |  |
| *Callithrix jacchus* | Common marmoset | Garanhuns | Pernambuco | -36.52828600000 | -8.88109200000 | Oliveira et al 2003 | Literature - checklist | |  |
| *Cerdocyon thous* | Crab eating fox | Garanhuns | Pernambuco | -36.52828600000 | -8.88109200000 | Oliveira et al 2003 | Literature - checklist | |  |
| *Conepatus semistriatus* | Striped hog nosed Skunk | Garanhuns | Pernambuco | -36.52828600000 | -8.88109200000 | Oliveira et al 2003 | Literature - checklist | |  |
| *Didelphis albiventris* | White-eared Opossum | Garanhuns | Pernambuco | -36.52828600000 | -8.88109200000 | Oliveira et al 2003 | Literature - checklist | |  |
| *Eira barbara* | Tayra | Garanhuns | Pernambuco | -36.52828600000 | -8.88109200000 | Oliveira et al 2003 | Literature - checklist | |  |
| *Galea spixii* | Spix's Yellow-toothed Cavy | Garanhuns | Pernambuco | -36.52828600000 | -8.88109200000 | Oliveira et al 2003 | Literature - checklist | |  |
| *Kerodon rupestris* | Rock Cavy | Garanhuns | Pernambuco | -36.52828600000 | -8.88109200000 | Oliveira et al 2003 | Literature - checklist | |  |
| *Procyon cancrivorus* | Crab eating Raccoon | Garanhuns | Pernambuco | -36.52828600000 | -8.88109200000 | Oliveira et al 2003 | Literature - checklist | |  |
| *Sylvilagus brasiliensis* | Tapeti | Garanhuns | Pernambuco | -36.52828600000 | -8.88109200000 | Oliveira et al 2003 | Literature - checklist | |  |
| *Callithrix jacchus* | Common marmoset | Iguatu | Ceara | -39.31666700000 | -5.50000000000 | Gbif | Observation | |  |
| *Dasypus novemcinctus* | Nine-banded Armadillo | Iguatu | Ceara | -39.31666700000 | -5.50000000000 | Oliveira et al 2003 | Literature - checklist | |  |
| *Didelphis albiventris* | White-eared Opossum | Iguatu | Ceara | -39.31666700000 | -5.50000000000 | Gbif | Observation | |  |
| *Euphractus sexcinctus* | Yellow Armadillo | Iguatu | Ceara | -39.31666700000 | -5.50000000000 | Oliveira et al 2003 | Literature - checklist | |  |
| *Galea spixii* | Spix's Yellow-toothed Cavy | Iguatu | Ceara | -39.31666700000 | -5.50000000000 | Gbif | Observation | |  |
| *Leopardus emiliae* | Tiger cat | Iguatu | Ceara | -39.31666700000 | -5.50000000000 | Feijo et al 2013 | Museum collection | |  |
| *Subulo gouazoubira* | Gray Brocket | Iguatu | Ceara | -39.31666700000 | -5.50000000000 | Feijo et al 2013 | Museum collection | |  |
| *Sapajus libidinosus* | Bearded Capuchin | Iguatu | Ceara | -39.31666700000 | -5.50000000000 | Gbif | Observation | |  |
| *Callithrix jacchus* | Common marmoset | Ipu | Ceara | -40.72381100000 | -4.31322800000 | Gbif | Collection | |  |
| *Coendou prehensilis* | Brazilian Porcupine | Ipu | Ceara | -40.72381100000 | -4.31322800000 | Oliveira et al 2003 | Literature - checklist | |  |
| *Dasypus novemcinctus* | Nine-banded Armadillo | Ipu | Ceara | -40.72381100000 | -4.31322800000 | Oliveira et al 2003 | Literature - checklist | |  |
| *Didelphis albiventris* | White-eared Opossum | Ipu | Ceara | -40.72381100000 | -4.31322800000 | Oliveira et al 2003 | Literature - checklist | |  |
| *Euphractus sexcinctus* | Yellow Armadillo | Ipu | Ceara | -40.72381100000 | -4.31322800000 | Feijo et al 2013, Oliveira et al 2003 | Museum collection | |  |
| *Galea spixii* | Spix's Yellow-toothed Cavy | Ipu | Ceara | -40.72381100000 | -4.31322800000 | Oliveira et al 2003 | Literature - checklist | |  |
| *Galictis cuja* | Lesser Grison | Ipu | Ceara | -40.72381100000 | -4.31322800000 | Oliveira et al 2003 | Literature - checklist | |  |
| *Kerodon rupestris* | Rock Cavy | Ipu | Ceara | -40.72381100000 | -4.31322800000 | Oliveira et al 2003 | Literature - checklist | |  |
| *Alouatta caraya* | Black-and-gold Howler monkey | Jaiba | Minas Gerais | -43.58858400000 | -15.32040100000 | Oliveira et al 2003 | Literature - checklist | |  |
| *Callithrix jacchus* | Common marmoset | Jaiba | Minas Gerais | -43.58858400000 | -15.32040100000 | Oliveira et al 2003 | Literature - checklist | |  |
| *Cerdocyon thous* | Crab eating fox | Jaiba | Minas Gerais | -43.58858400000 | -15.32040100000 | Oliveira et al 2003 | Literature - checklist | |  |
| *Coendou prehensilis* | Brazilian Porcupine | Jaiba | Minas Gerais | -43.58858400000 | -15.32040100000 | Oliveira et al 2003 | Literature - checklist | |  |
| *Dasypus novemcinctus* | Nine-banded Armadillo | Jaiba | Minas Gerais | -43.58858400000 | -15.32040100000 | Oliveira et al 2003 | Literature - checklist | |  |
| *Didelphis albiventris* | White-eared Opossum | Jaiba | Minas Gerais | -43.58858400000 | -15.32040100000 | Oliveira et al 2003 | Literature - checklist | |  |
| *Euphractus sexcinctus* | Yellow Armadillo | Jaiba | Minas Gerais | -43.58858400000 | -15.32040100000 | Oliveira et al 2003 | Literature - checklist | |  |
| *Galea spixii* | Spix's Yellow-toothed Cavy | Jaiba | Minas Gerais | -43.58858400000 | -15.32040100000 | Oliveira et al 2003 | Literature - checklist | |  |
| *Herpailurus yagouaroundi* | Jaguarundi | Jaiba | Minas Gerais | -43.58858400000 | -15.32040100000 | Oliveira et al 2003 | Literature - checklist | |  |
| *Leopardus emiliae* | Tiger cat | Jaiba | Minas Gerais | -43.58858400000 | -15.32040100000 | Oliveira et al 2003 | Literature - checklist | |  |
| *Mazama americana* | Red Brocket | Jaiba | Minas Gerais | -43.58858400000 | -15.32040100000 | Oliveira et al 2003 | Literature - checklist | |  |
| *Procyon cancrivorus* | Crab eating Raccoon | Jaiba | Minas Gerais | -43.58858400000 | -15.32040100000 | Oliveira et al 2003 | Literature - checklist | |  |
| *Sapajus apella* | Black capped Capuchin | Jaiba | Minas Gerais | -43.58858400000 | -15.32040100000 | Oliveira et al 2003 | Literature - checklist | |  |
| *Sylvilagus brasiliensis* | Tapeti | Jaiba | Minas Gerais | -43.58858400000 | -15.32040100000 | Oliveira et al 2003 | Literature - checklist | |  |
| *Tapirus terrestris* | Lowlander Tapir | Jaiba | Minas Gerais | -43.58858400000 | -15.32040100000 | Oliveira et al 2003 | Literature - checklist | |  |
| *Cavia aperea* | Brazilian guinea pig | Jardim de Piranhas | Rio Grande do Norte | -37.34226600000 | -6.37875300000 | Barbosa et al 2016 | Interview - hunter | |  |
| *Cerdocyon thous* | Crab eating fox | Jardim de Piranhas | Rio Grande do Norte | -37.34226600000 | -6.37875300000 | Barbosa et al 2016 | Interview - hunter | |  |
| *Conepatus semistriatus* | Striped hog nosed Skunk | Jardim de Piranhas | Rio Grande do Norte | -37.34226600000 | -6.37875300000 | Barbosa et al 2016 | Interview - hunter | |  |
| *Dasyprocta prymnolopha* | red-orange rump agoutis | Jardim de Piranhas | Rio Grande do Norte | -37.34226600000 | -6.37875300000 | Barbosa et al 2016 | Interview - hunter | |  |
| *Dasypus novemcinctus* | Nine-banded Armadillo | Jardim de Piranhas | Rio Grande do Norte | -37.34226600000 | -6.37875300000 | Barbosa et al 2016 | Interview - hunter | |  |
| *Didelphis marsupialis* | Common Opossum | Jardim de Piranhas | Rio Grande do Norte | -37.34226600000 | -6.37875300000 | Barbosa et al 2016 | Interview - hunter | |  |
| *Euphractus sexcinctus* | Yellow Armadillo | Jardim de Piranhas | Rio Grande do Norte | -37.34226600000 | -6.37875300000 | Barbosa et al 2016 | Interview - hunter | |  |
| *Galea spixii* | Spix's Yellow-toothed Cavy | Jardim de Piranhas | Rio Grande do Norte | -37.34226600000 | -6.37875300000 | Barbosa et al 2016 | Interview - hunter | |  |
| *Galictis vittata* | Greater Grison | Jardim de Piranhas | Rio Grande do Norte | -37.34226600000 | -6.37875300000 | Barbosa et al 2016 | Interview - hunter | |  |
| *Herpailurus yagouaroundi* | Jaguarundi | Jardim de Piranhas | Rio Grande do Norte | -37.34226600000 | -6.37875300000 | Barbosa et al 2016 | Interview - hunter | |  |
| *Kerodon rupestris* | Rock Cavy | Jardim de Piranhas | Rio Grande do Norte | -37.34226600000 | -6.37875300000 | Barbosa et al 2016 | Interview - hunter | |  |
| *Leopardus emiliae* | Tiger cat | Jardim de Piranhas | Rio Grande do Norte | -37.34226600000 | -6.37875300000 | Barbosa et al 2016 | Interview - hunter | |  |
| *Leopardus pardalis* | Ocelot | Jardim de Piranhas | Rio Grande do Norte | -37.34226600000 | -6.37875300000 | Barbosa et al 2016 | Interview - hunter | |  |
| *Leopardus wiedii* | Margay | Jardim de Piranhas | Rio Grande do Norte | -37.34226600000 | -6.37875300000 | Barbosa et al 2016 | Interview - hunter | |  |
| *Mazama americana* | Red Brocket | Jardim de Piranhas | Rio Grande do Norte | -37.34226600000 | -6.37875300000 | Barbosa et al 2016 | Interview - hunter | |  |
| *Subulo gouazoubira* | Gray Brocket | Jardim de Piranhas | Rio Grande do Norte | -37.34226600000 | -6.37875300000 | Barbosa et al 2016 | Interview - hunter | |  |
| *Procyon cancrivorus* | Crab eating Raccoon | Jardim de Piranhas | Rio Grande do Norte | -37.34226600000 | -6.37875300000 | Barbosa et al 2016 | Interview - hunter | |  |
| *Tamandua tetradactyla* | Southern Tamandua | Jardim de Piranhas | Rio Grande do Norte | -37.34226600000 | -6.37875300000 | Barbosa et al 2016 | Interview - hunter | |  |
| *Callithrix jacchus* | Common marmoset | Jua | Ceara | -39.29999900000 | -6.36666700000 | Gbif | Collection | |  |
| *Cerdocyon thous* | Crab eating fox | Jua | Ceara | -39.29999900000 | -6.36666700000 | Gbif | Collection | |  |
| *Didelphis albiventris* | White-eared Opossum | Jua | Ceara | -39.29999900000 | -6.36666700000 | Gbif | Collection | |  |
| *Galea spixii* | Spix's Yellow-toothed Cavy | Jua | Ceara | -39.29999900000 | -6.36666700000 | Gbif | Collection | |  |
| *Kerodon rupestris* | Rock Cavy | Jua | Ceara | -39.29999900000 | -6.36666700000 | Gbif | Collection | |  |
| *Cerdocyon thous* | Crab eating fox | Lagoa | Paraiba | -37.91050600000 | -6.61369800000 | Vasconcelos Neto et al 2012 | Interview - hunter | |  |
| *Conepatus semistriatus* | Striped hog nosed Skunk | Lagoa | Paraiba | -37.91050600000 | -6.61369800000 | Vasconcelos Neto et al 2012 | Interview - hunter | |  |
| *Dasypus novemcinctus* | Nine-banded Armadillo | Lagoa | Paraiba | -37.91050600000 | -6.61369800000 | Vasconcelos Neto et al 2012 | Interview - hunter | |  |
| *Euphractus sexcinctus* | Yellow Armadillo | Lagoa | Paraiba | -37.91050600000 | -6.61369800000 | Vasconcelos Neto et al 2012 | Interview - hunter | |  |
| *Herpailurus yagouaroundi* | Jaguarundi | Lagoa | Paraiba | -37.91050600000 | -6.61369800000 | Vasconcelos Neto et al 2012 | Interview - hunter | |  |
| *Leopardus emiliae* | Tiger cat | Lagoa | Paraiba | -37.91050600000 | -6.61369800000 | Vasconcelos Neto et al 2012 | Interview - hunter | |  |
| *Leopardus pardalis* | Ocelot | Lagoa | Paraiba | -37.91050600000 | -6.61369800000 | Vasconcelos Neto et al 2012 | Interview - hunter | |  |
| *Leopardus wiedii* | Margay | Lagoa | Paraiba | -37.91050600000 | -6.61369800000 | Vasconcelos Neto et al 2012 | Interview - hunter | |  |
| *Nasua nasua* | South American Coati | Lagoa | Paraiba | -37.91050600000 | -6.61369800000 | Vasconcelos Neto et al 2012 | Interview - hunter | |  |
| *Dicotyles tajacu* | Collaredy Pecary | Lagoa | Paraiba | -37.91050600000 | -6.61369800000 | Vasconcelos Neto et al 2012 | Interview - hunter | |  |
| *Procyon cancrivorus* | Crab eating Raccoon | Lagoa | Paraiba | -37.91050600000 | -6.61369800000 | Vasconcelos Neto et al 2012 | Interview - hunter | |  |
| *Sapajus libidinosus* | Bearded Capuchin | Lagoa | Paraiba | -37.91050600000 | -6.61369800000 | Vasconcelos Neto et al 2012 | Interview - hunter | |  |
| *Tamandua tetradactyla* | Southern Tamandua | Lagoa | Paraiba | -37.91050600000 | -6.61369800000 | Vasconcelos Neto et al 2012 | Interview - hunter | |  |
| *Cabassous tatouay* | Greater Naked-tailed Armadillo | Lagoa Grande | Pernambuco | -40.17447500000 | -8.79881000000 | Valle 2007 dissertacao | Interview - checklist | |  |
| *Callithrix jacchus* | Common marmoset | Lagoa Grande | Pernambuco | -40.17447500000 | -8.79881000000 | Valle 2007 dissertacao | Capture, interview - checklist | |  |
| *Cerdocyon thous* | Crab eating fox | Lagoa Grande | Pernambuco | -40.17447500000 | -8.79881000000 | Valle 2007 dissertacao | Capture, interview - checklist | |  |
| *Conepatus semistriatus* | Striped hog nosed Skunk | Lagoa Grande | Pernambuco | -40.17447500000 | -8.79881000000 | Valle 2007 dissertacao | Interview - checklist | |  |
| *Dasyprocta prymnolopha* | red-orange rump agoutis | Lagoa Grande | Pernambuco | -40.17447500000 | -8.79881000000 | Valle 2007 dissertacao | Interview - checklist | |  |
| *Dasypus novemcinctus* | Nine-banded Armadillo | Lagoa Grande | Pernambuco | -40.17447500000 | -8.79881000000 | Valle 2007 dissertacao, Feijo et al 2013 | Capture, interview - checklist | |  |
| *Dasypus septemcinctus* | Brazilian Lesser long-nosed Armadillo | Lagoa Grande | Pernambuco | -40.17447500000 | -8.79881000000 | Valle 2007 dissertacao, Feijo et al 2013 | Capture, interview - checklist | |  |
| *Eira barbara* | Tayra | Lagoa Grande | Pernambuco | -40.17447500000 | -8.79881000000 | Valle 2007 dissertacao | Interview - checklist | |  |
| *Euphractus sexcinctus* | Yellow Armadillo | Lagoa Grande | Pernambuco | -40.17447500000 | -8.79881000000 | Valle 2007 dissertacao | Capture, interview - checklist | |  |
| *Galea spixii* | Spix's Yellow-toothed Cavy | Lagoa Grande | Pernambuco | -40.17447500000 | -8.79881000000 | Valle 2007 dissertacao | Interview - checklist | |  |
| *Galictis vittata* | Greater Grison | Lagoa Grande | Pernambuco | -40.17447500000 | -8.79881000000 | Valle 2007 dissertacao | Interview - checklist | |  |
| *Herpailurus yagouaroundi* | Jaguarundi | Lagoa Grande | Pernambuco | -40.17447500000 | -8.79881000000 | Valle 2007 dissertacao | Interview - checklist | |  |
| *Hydrochoerus hydrochaeris* | Capybara | Lagoa Grande | Pernambuco | -40.17447500000 | -8.79881000000 | Valle 2007 dissertacao | Interview - checklist | |  |
| *Kerodon rupestris* | Rock Cavy | Lagoa Grande | Pernambuco | -40.17447500000 | -8.79881000000 | Valle 2007 dissertacao | Interview - checklist | |  |
| *Leopardus emiliae* | Tiger cat | Lagoa Grande | Pernambuco | -40.17447500000 | -8.79881000000 | Valle 2007 dissertacao | Interview - checklist | |  |
| *Leopardus pardalis* | Ocelot | Lagoa Grande | Pernambuco | -40.17447500000 | -8.79881000000 | Valle 2007 dissertacao | Interview - checklist | |  |
| *Leopardus wiedii* | Margay | Lagoa Grande | Pernambuco | -40.17447500000 | -8.79881000000 | Valle 2007 dissertacao | Interview - checklist | |  |
| *Subulo gouazoubira* | Gray Brocket | Lagoa Grande | Pernambuco | -40.17447500000 | -8.79881000000 | Valle 2007 dissertacao | Capture, interview - checklist | |  |
| *Panthera onca* | Jaguar | Lagoa Grande | Pernambuco | -40.17447500000 | -8.79881000000 | Valle 2007 dissertacao | Interview - checklist | |  |
| *Dicotyles tajacu* | Collaredy Pecary | Lagoa Grande | Pernambuco | -40.17447500000 | -8.79881000000 | Valle 2007 dissertacao | Capture, interview - checklist | |  |
| *Procyon cancrivorus* | Crab eating Raccoon | Lagoa Grande | Pernambuco | -40.17447500000 | -8.79881000000 | Valle 2007 dissertacao | Interview - checklist | |  |
| *Puma concolor* | Puma | Lagoa Grande | Pernambuco | -40.17447500000 | -8.79881000000 | Valle 2007 dissertacao | Interview - checklist | |  |
| *Tamandua tetradactyla* | Southern Tamandua | Lagoa Grande | Pernambuco | -40.17447500000 | -8.79881000000 | Valle 2007 dissertacao | Interview - checklist | |  |
| *Tayassu* *pecari* | White-lipped Peccary | Lagoa Grande | Pernambuco | -40.17447500000 | -8.79881000000 | Valle 2007 dissertacao | Interview - checklist | |  |
| *Tolypeutes tricinctus* | Brazilian Three banded Armadillo | Lagoa Grande | Pernambuco | -40.17447500000 | -8.79881000000 | Valle 2007 dissertacao, Feijo et al 2013 | Interview - checklist | |  |
| *Cerdocyon thous* | Crab eating fox | Lagoa Nova | Rio Grande do Norte | -36.57763300000 | -6.03575800000 | Marinho et al 2018 | Camera trapping - checklist | |  |
| *Conepatus semistriatus* | Striped hog nosed Skunk | Lagoa Nova | Rio Grande do Norte | -36.57763300000 | -6.03575800000 | Marinho et al 2018 | Camera trapping - checklist | |  |
| *Didelphis albiventris* | White-eared Opossum | Lagoa Nova | Rio Grande do Norte | -36.57763300000 | -6.03575800000 | Marinho et al 2018 | Camera trapping - checklist | |  |
| *Euphractus sexcinctus* | Yellow Armadillo | Lagoa Nova | Rio Grande do Norte | -36.57763300000 | -6.03575800000 | Marinho et al 2018 | Camera trapping - checklist | |  |
| *Leopardus emiliae* | Tiger cat | Lagoa Nova | Rio Grande do Norte | -36.57763300000 | -6.03575800000 | Marinho et al 2018 | Camera trapping - checklist | |  |
| *Procyon cancrivorus* | Crab eating Raccoon | Lagoa Nova | Rio Grande do Norte | -36.57763300000 | -6.03575800000 | Marinho et al 2018 | Camera trapping - checklist | |  |
| *Sapajus libidinosus* | Bearded Capuchin | Lagoa Nova | Rio Grande do Norte | -36.57763300000 | -6.03575800000 | Marinho et al 2018 | Camera trapping - checklist | |  |
| *Tamandua tetradactyla* | Southern Tamandua | Lagoa Nova | Rio Grande do Norte | -36.57763300000 | -6.03575800000 | Marinho et al 2018 | Camera trapping - checklist | |  |
| *Cerdocyon thous* | Crab eating fox | Luis Gomes | Rio Grande do Norte | -38.38627800000 | -6.39641700000 | Marinho et al 2018 | Camera trapping - checklist | |  |
| *Dasypus novemcinctus* | Nine-banded Armadillo | Luis Gomes | Rio Grande do Norte | -38.38627800000 | -6.39641700000 | Marinho et al 2018 | Camera trapping - checklist | |  |
| *Didelphis albiventris* | White-eared Opossum | Luis Gomes | Rio Grande do Norte | -38.38627800000 | -6.39641700000 | Marinho et al 2018 | Camera trapping - checklist | |  |
| *Euphractus sexcinctus* | Yellow Armadillo | Luis Gomes | Rio Grande do Norte | -38.38627800000 | -6.39641700000 | Marinho et al 2018 | Camera trapping - checklist | |  |
| *Leopardus emiliae* | Tiger cat | Luis Gomes | Rio Grande do Norte | -38.38627800000 | -6.39641700000 | Marinho et al 2018 | Camera trapping - checklist | |  |
| *Subulo gouazoubira* | Gray Brocket | Luis Gomes | Rio Grande do Norte | -38.38627800000 | -6.39641700000 | Marinho et al 2018 | Camera trapping - checklist | |  |
| *Procyon cancrivorus* | Crab eating Raccoon | Luis Gomes | Rio Grande do Norte | -38.38627800000 | -6.39641700000 | Marinho et al 2018 | Camera trapping - checklist | |  |
| *Puma concolor* | Puma | Luis Gomes | Rio Grande do Norte | -38.38627800000 | -6.39641700000 | Marinho et al 2018 | Camera trapping - checklist | |  |
| *Sapajus libidinosus* | Bearded Capuchin | Luis Gomes | Rio Grande do Norte | -38.38627800000 | -6.39641700000 | Marinho et al 2018, Emidio & Ferreira 2012 | Camera trapping - checklist | |  |
| *Cerdocyon thous* | Crab eating fox | Martins | Rio Grande do Norte | -37.88678600000 | -6.02824400000 | Marinho et al 2018 | Camera trapping - checklist | |  |
| *Dasypus novemcinctus* | Nine-banded Armadillo | Martins | Rio Grande do Norte | -37.88678600000 | -6.02824400000 | Marinho et al 2018 | Camera trapping - checklist | |  |
| *Didelphis albiventris* | White-eared Opossum | Martins | Rio Grande do Norte | -37.88678600000 | -6.02824400000 | Marinho et al 2018 | Camera trapping - checklist | |  |
| *Euphractus sexcinctus* | Yellow Armadillo | Martins | Rio Grande do Norte | -37.88678600000 | -6.02824400000 | Marinho et al 2018 | Camera trapping - checklist | |  |
| *Leopardus emiliae* | Tiger cat | Martins | Rio Grande do Norte | -37.88678600000 | -6.02824400000 | Marinho et al 2018 | Camera trapping - checklist | |  |
| *Subulo gouazoubira* | Gray Brocket | Martins | Rio Grande do Norte | -37.88678600000 | -6.02824400000 | Marinho et al 2018 | Camera trapping - checklist | |  |
| *Procyon cancrivorus* | Crab eating Raccoon | Martins | Rio Grande do Norte | -37.88678600000 | -6.02824400000 | Marinho et al 2018 | Camera trapping - checklist | |  |
| *Sapajus libidinosus* | Bearded Capuchin | Martins | Rio Grande do Norte | -37.88678600000 | -6.02824400000 | Marinho et al 2018 | Camera trapping - checklist | |  |
| *Callithrix jacchus* | Common marmoset | Monumento Natural Grota do Angico | Sergipe | -37.68079200000 | -9.65420800000 | Freitas et al 2016 | Indirect evidence, camera trapping - checklist | |  |
| *Cerdocyon thous* | Crab eating fox | Monumento Natural Grota do Angico | Sergipe | -37.68079200000 | -9.65420800000 | Dias & Bocchiglieri 2016, Freitas et al 2016 | Indirect evidence, camera trapping - checklist | |  |
| *Conepatus semistriatus* | Striped hog nosed Skunk | Monumento Natural Grota do Angico | Sergipe | -37.68079200000 | -9.65420800000 | Dias & Bocchiglieri 2016, Freitas et al 2016 | Indirect evidence, camera trapping - checklist | |  |
| *Didelphis albiventris* | White-eared Opossum | Monumento Natural Grota do Angico | Sergipe | -37.68079200000 | -9.65420800000 | Freitas et al 2016 | Direct evidence - checklist | |  |
| *Euphractus sexcinctus* | Yellow Armadillo | Monumento Natural Grota do Angico | Sergipe | -37.68079200000 | -9.65420800000 | Dias & Bocchiglieri 2016, Freitas et al 2016 | Indirect evidence, camera trapping - checklist | |  |
| *Herpailurus yagouaroundi* | Jaguarundi | Monumento Natural Grota do Angico | Sergipe | -37.68079200000 | -9.65420800000 | Dias & Bocchiglieri 2016, Freitas et al 2016 | Indirect evidence, camera trapping - checklist | |  |
| *Hydrochoerus hydrochaeris* | Capybara | Monumento Natural Grota do Angico | Sergipe | -37.68079200000 | -9.65420800000 | Dias & Bocchiglieri 2016, Freitas et al 2016 | Indirect evidence, camera trapping - checklist | |  |
| *Kerodon rupestris* | Rock Cavy | Monumento Natural Grota do Angico | Sergipe | -37.68079200000 | -9.65420800000 | Dias & Bocchiglieri 2016, Freitas et al 2016, Oliveira et al 2003 | Indirect evidence, camera trapping - checklist | |  |
| *Leopardus emiliae* | Tiger cat | Monumento Natural Grota do Angico | Sergipe | -37.68079200000 | -9.65420800000 | Dias & Bocchiglieri 2016, Freitas et al 2016 | Indirect evidence, camera trapping - checklist | |  |
| *Leopardus pardalis* | Ocelot | Monumento Natural Grota do Angico | Sergipe | -37.68079200000 | -9.65420800000 | Dias & Bocchiglieri 2016, Freitas et al 2016 | Indirect evidence, camera trapping - checklist | |  |
| *Lontra longicaudis* | Neothropical Otter | Monumento Natural Grota do Angico | Sergipe | -37.68079200000 | -9.65420800000 | Dias & Bocchiglieri 2016, Freitas et al 2016 | Indirect evidence, camera trapping - checklist | |  |
| *Subulo gouazoubira* | Gray Brocket | Monumento Natural Grota do Angico | Sergipe | -37.68079200000 | -9.65420800000 | Dias & Bocchiglieri 2016, Freitas et al 2016 | Indirect evidence, camera trapping - checklist | |  |
| *Dicotyles tajacu* | Collaredy Pecary | Monumento Natural Grota do Angico | Sergipe | -37.68079200000 | -9.65420800000 | Oliveira et al 2003 | Literature - checklist | |  |
| *Procyon cancrivorus* | Crab eating Raccoon | Monumento Natural Grota do Angico | Sergipe | -37.68079200000 | -9.65420800000 | Dias & Bocchiglieri 2016, Freitas et al 2016 | Indirect evidence, camera trapping - checklist | |  |
| *Tamandua tetradactyla* | Southern Tamandua | Monumento Natural Grota do Angico | Sergipe | -37.68079200000 | -9.65420800000 | Dias & Bocchiglieri 2016, Freitas et al 2016, Oliveira et al 2003 | Indirect evidence, camera trapping - checklist | |  |
| *Cuniculus paca* | Agouti | Morada Nova | Ceara | -38.50931800000 | -5.15682800000 | Species link | Collection | |  |
| *Dasyprocta prymnolopha* | red-orange rump agoutis | Morada Nova | Ceara | -38.50931800000 | -5.15682800000 | Species link | Collection | |  |
| *Dasypus novemcinctus* | Nine-banded Armadillo | Morada Nova | Ceara | -38.50931800000 | -5.15682800000 | Species link | Collection | |  |
| *Euphractus sexcinctus* | Yellow Armadillo | Morada Nova | Ceara | -38.50931800000 | -5.15682800000 | Gbif | Observation | |  |
| *Mazama americana* | Red Brocket | Morada Nova | Ceara | -38.50931800000 | -5.15682800000 | Species link | Collection | |  |
| *Dicotyles tajacu* | Collaredy Pecary | Morada Nova | Ceara | -38.50931800000 | -5.15682800000 | Species link | Collection | |  |
| *Bradypus variegatus* | Brown-throated Sloth | Morro do Chapeu | Bahia | -41.17440700000 | -11.32336000000 | Gbif | Collection | |  |
| *Callithrix jacchus* | Common marmoset | Morro do Chapeu | Bahia | -41.17440700000 | -11.32336000000 | Gbif | Collection | |  |
| *Callithrix penicillata* | Black pencilled marmoset | Morro do Chapeu | Bahia | -41.17440700000 | -11.32336000000 | Gbif | Observation | |  |
| *Cavia aperea* | Brazilian guinea pig | Morro do Chapeu | Bahia | -41.17440700000 | -11.32336000000 | Gbif | Collection | |  |
| *Cerdocyon thous* | Crab eating fox | Morro do Chapeu | Bahia | -41.17440700000 | -11.32336000000 | Gbif | Collection | |  |
| *Conepatus semistriatus* | Striped hog nosed Skunk | Morro do Chapeu | Bahia | -41.17440700000 | -11.32336000000 | Gbif | Collection | |  |
| *Cuniculus paca* | Agouti | Morro do Chapeu | Bahia | -41.17440700000 | -11.32336000000 | Gbif | Collection | |  |
| *Dasyprocta leporina* | Red humped agouti | Morro do Chapeu | Bahia | -41.17440700000 | -11.32336000000 | Gbif | Collection | |  |
| *Dasyprocta prymnolopha* | red-orange rump agoutis | Morro do Chapeu | Bahia | -41.17440700000 | -11.32336000000 | Gbif | Collection | |  |
| *Dasypus novemcinctus* | Nine-banded Armadillo | Morro do Chapeu | Bahia | -41.17440700000 | -11.32336000000 | Gbif | Collection | |  |
| *Didelphis albiventris* | White-eared Opossum | Morro do Chapeu | Bahia | -41.17440700000 | -11.32336000000 | Gbif | Collection | |  |
| *Eira barbara* | Tayra | Morro do Chapeu | Bahia | -41.17440700000 | -11.32336000000 | Gbif | Collection | |  |
| *Euphractus sexcinctus* | Yellow Armadillo | Morro do Chapeu | Bahia | -41.17440700000 | -11.32336000000 | Gbif | Collection | |  |
| *Galea spixii* | Spix's Yellow-toothed Cavy | Morro do Chapeu | Bahia | -41.17440700000 | -11.32336000000 | Gbif | Collection | |  |
| *Galictis vittata* | Greater Grison | Morro do Chapeu | Bahia | -41.17440700000 | -11.32336000000 | Gbif | Collection | |  |
| *Hydrochoerus hydrochaeris* | Capybara | Morro do Chapeu | Bahia | -41.17440700000 | -11.32336000000 | Gbif | Collection | |  |
| *Kerodon rupestris* | Rock Cavy | Morro do Chapeu | Bahia | -41.17440700000 | -11.32336000000 | Gbif | Collection | |  |
| *Leopardus emiliae* | Tiger cat | Morro do Chapeu | Bahia | -41.17440700000 | -11.32336000000 | Gbif | Collection | |  |
| *Leopardus pardalis* | Ocelot | Morro do Chapeu | Bahia | -41.17440700000 | -11.32336000000 | Gbif | Collection | |  |
| *Leopardus wiedii* | Margay | Morro do Chapeu | Bahia | -41.17440700000 | -11.32336000000 | Gbif | Collection | |  |
| *Mazama americana* | Red Brocket | Morro do Chapeu | Bahia | -41.17440700000 | -11.32336000000 | Gbif | Collection | |  |
| *Subulo gouazoubira* | Gray Brocket | Morro do Chapeu | Bahia | -41.17440700000 | -11.32336000000 | Gbif | Collection | |  |
| *Ozotoceros bezoarticus* | Pampas deer | Morro do Chapeu | Bahia | -41.17440700000 | -11.32336000000 | Gbif | Collection | |  |
| *Dicotyles tajacu* | Collaredy Pecary | Morro do Chapeu | Bahia | -41.17440700000 | -11.32336000000 | Gbif | Collection | |  |
| *Procyon cancrivorus* | Crab eating Raccoon | Morro do Chapeu | Bahia | -41.17440700000 | -11.32336000000 | Gbif | Collection | |  |
| *Puma concolor* | Puma | Morro do Chapeu | Bahia | -41.17440700000 | -11.32336000000 | Gbif | Collection | |  |
| *Sapajus apella* | Black capped Capuchin | Morro do Chapeu | Bahia | -41.17440700000 | -11.32336000000 | Gbif | Collection | |  |
| *Speothos venaticus* | Bush Dog | Morro do Chapeu | Bahia | -41.17440700000 | -11.32336000000 | Gbif | Collection | |  |
| *Tamandua tetradactyla* | Southern Tamandua | Morro do Chapeu | Bahia | -41.17440700000 | -11.32336000000 | Gbif | Collection | |  |
| *Tapirus terrestris* | Lowlander Tapir | Morro do Chapeu | Bahia | -41.17440700000 | -11.32336000000 | Gbif | Collection | |  |
| *Tolypeutes tricinctus* | Brazilian Three banded Armadillo | Morro do Chapeu | Bahia | -41.17440700000 | -11.32336000000 | Gbif | Collection | |  |
| *Callithrix jacchus* | Common marmoset | Nova Palmeira | Paraiba | -36.40828200000 | -6.68550900000 | Gbif | Observation | |  |
| *Cerdocyon thous* | Crab eating fox | Nova Palmeira | Paraiba | -36.40828200000 | -6.68550900000 | Gbif | Observation | |  |
| *Didelphis albiventris* | White-eared Opossum | Nova Palmeira | Paraiba | -36.40828200000 | -6.68550900000 | Gbif | Observation | |  |
| *Euphractus sexcinctus* | Yellow Armadillo | Nova Palmeira | Paraiba | -36.40828200000 | -6.68550900000 | Gbif | Observation | |  |
| *Galea spixii* | Spix's Yellow-toothed Cavy | Nova Palmeira | Paraiba | -36.40828200000 | -6.68550900000 | Gbif | Observation | |  |
| *Kerodon rupestris* | Rock Cavy | Nova Palmeira | Paraiba | -36.40828200000 | -6.68550900000 | Gbif | Observation | |  |
| *Callithrix jacchus* | Common marmoset | Ouricuri | Pernambuco | -40.09888900000 | -7.81366700000 | Dellcielos et al 2016 | Camera trappings, indirect evidence - checklist | |  |
| *Cerdocyon thous* | Crab eating fox | Ouricuri | Pernambuco | -40.09888900000 | -7.81366700000 | Dellcielos et al 2016 | Camera trappings, indirect evidence - checklist | |  |
| *Conepatus semistriatus* | Striped hog nosed Skunk | Ouricuri | Pernambuco | -40.09888900000 | -7.81366700000 | Dellcielos et al 2016 | Camera trappings, indirect evidence - checklist | |  |
| *Euphractus sexcinctus* | Yellow Armadillo | Ouricuri | Pernambuco | -40.09888900000 | -7.81366700000 | Dellcielos et al 2016 | Camera trappings, indirect evidence - checklist | |  |
| *Galea spixii* | Spix's Yellow-toothed Cavy | Ouricuri | Pernambuco | -40.09888900000 | -7.81366700000 | Dellcielos et al 2016 | Camera trappings, indirect evidence - checklist | |  |
| *Herpailurus yagouaroundi* | Jaguarundi | Ouricuri | Pernambuco | -40.09888900000 | -7.81366700000 | Feijo et al 2013 | Interview | |  |
| *Kerodon rupestris* | Rock Cavy | Ouricuri | Pernambuco | -40.09888900000 | -7.81366700000 | Dellcielos et al 2016 | Camera trappings, indirect evidence - checklist | |  |
| *Leopardus emiliae* | Tiger cat | Ouricuri | Pernambuco | -40.09888900000 | -7.81366700000 | Dellcielos et al 2016 | Camera trappings, indirect evidence - checklist | |  |
| *Leopardus pardalis* | Ocelot | Ouricuri | Pernambuco | -40.09888900000 | -7.81366700000 | Dellcielos et al 2016 | Camera trappings, indirect evidence - checklist | |  |
| *Subulo gouazoubira* | Gray Brocket | Ouricuri | Pernambuco | -40.09888900000 | -7.81366700000 | Dellcielos et al 2016 | Camera trappings, indirect evidence - checklist | |  |
| *Procyon cancrivorus* | Crab eating Raccoon | Ouricuri | Pernambuco | -40.09888900000 | -7.81366700000 | Dellcielos et al 2016 | Camera trappings, indirect evidence - checklist | |  |
| *Callithrix jacchus* | Common marmoset | Parque Estadual Mata do Pau Ferro | Paraiba | -35.74957100000 | -6.96938400000 | Feijo et al 2013 | Interview,Museum collection | |  |
| *Cerdocyon thous* | Crab eating fox | Parque Estadual Mata do Pau Ferro | Paraiba | -35.74957100000 | -6.96938400000 | Feijo et al 2013 | Interview | |  |
| *Conepatus semistriatus* | Striped hog nosed Skunk | Parque Estadual Mata do Pau Ferro | Paraiba | -35.74957100000 | -6.96938400000 | Feijo et al 2013 | Interview | |  |
| *Sylvilagus brasiliensis* | Tapeti | Parque Estadual Mata do Pau Ferro | Paraiba | -35.74957100000 | -6.96938400000 | Feijo et al 2013 | Interview,Museum collection | |  |
| *Tamandua tetradactyla* | Southern Tamandua | Parque Estadual Mata do Pau Ferro | Paraiba | -35.74957100000 | -6.96938400000 | Feijo et al 2013 | Interview,Museum collection | |  |
| *Callithrix jacchus* | Common marmoset | Parque Estadual Serra da Pimenteira | Pernambuco | -38.29980200000 | -7.93315900000 | Santos et al 2013 - Livro | Interview - checklist | |  |
| *Cerdocyon thous* | Crab eating fox | Parque Estadual Serra da Pimenteira | Pernambuco | -38.29980200000 | -7.93315900000 | Santos et al 2013 - Livro | Interview - checklist | |  |
| *Conepatus semistriatus* | Striped hog nosed Skunk | Parque Estadual Serra da Pimenteira | Pernambuco | -38.29980200000 | -7.93315900000 | Santos et al 2013 - Livro | Interview - checklist | |  |
| *Dasypus novemcinctus* | Nine-banded Armadillo | Parque Estadual Serra da Pimenteira | Pernambuco | -38.29980200000 | -7.93315900000 | Santos et al 2013 - Livro | Interview - checklist | |  |
| *Euphractus sexcinctus* | Yellow Armadillo | Parque Estadual Serra da Pimenteira | Pernambuco | -38.29980200000 | -7.93315900000 | Santos et al 2013 - Livro | Interview - checklist | |  |
| *Galea spixii* | Spix's Yellow-toothed Cavy | Parque Estadual Serra da Pimenteira | Pernambuco | -38.29980200000 | -7.93315900000 | Santos et al 2013 - Livro | Interview - checklist | |  |
| *Herpailurus yagouaroundi* | Jaguarundi | Parque Estadual Serra da Pimenteira | Pernambuco | -38.29980200000 | -7.93315900000 | Santos et al 2013 - Livro | Interview - checklist | |  |
| *Kerodon rupestris* | Rock Cavy | Parque Estadual Serra da Pimenteira | Pernambuco | -38.29980200000 | -7.93315900000 | Santos et al 2013 - Livro | Interview - checklist | |  |
| *Leopardus emiliae* | Tiger cat | Parque Estadual Serra da Pimenteira | Pernambuco | -38.29980200000 | -7.93315900000 | Santos et al 2013 - Livro | Interview - checklist | |  |
| *Subulo gouazoubira* | Gray Brocket | Parque Estadual Serra da Pimenteira | Pernambuco | -38.29980200000 | -7.93315900000 | Santos et al 2013 - Livro | Interview - checklist | |  |
| *Procyon cancrivorus* | Crab eating Raccoon | Parque Estadual Serra da Pimenteira | Pernambuco | -38.29980200000 | -7.93315900000 | Santos et al 2013 - Livro | Interview - checklist | |  |
| *Puma concolor* | Puma | Parque Estadual Serra da Pimenteira | Pernambuco | -38.29980200000 | -7.93315900000 | Santos et al 2013 - Livro | Interview - checklist | |  |
| *Sapajus libidinosus* | Bearded Capuchin | Parque Estadual Serra da Pimenteira | Pernambuco | -38.29980200000 | -7.93315900000 | Santos et al 2013 - Livro | Interview - checklist | |  |
| *Sylvilagus brasiliensis* | Tapeti | Parque Estadual Serra da Pimenteira | Pernambuco | -38.29980200000 | -7.93315900000 | Santos et al 2013 - Livro | Interview - checklist | |  |
| *Tamandua tetradactyla* | Southern Tamandua | Parque Estadual Serra da Pimenteira | Pernambuco | -38.29980200000 | -7.93315900000 | Santos et al 2013 - Livro | Interview - checklist | |  |
| *Alouatta caraya* | Black-and-gold Howler monkey | Parque Nacional da Serra da Capivara | Piaui | -42.64065100000 | -8.83979400000 | Gbif | Observation | |  |
| *Callithrix jacchus* | Common marmoset | Parque Nacional da Serra da Capivara | Piaui | -42.64065100000 | -8.83979400000 | Gbif | Observation | |  |
| *Cerdocyon thous* | Crab eating fox | Parque Nacional da Serra da Capivara | Piaui | -42.64065100000 | -8.83979400000 | Gbif | Collection | |  |
| *Conepatus semistriatus* | Striped hog nosed Skunk | Parque Nacional da Serra da Capivara | Piaui | -42.64065100000 | -8.83979400000 | Oliveira et al 2003 | Literature - checklist | |  |
| *Cuniculus paca* | Agouti | Parque Nacional da Serra da Capivara | Piaui | -42.64065100000 | -8.83979400000 | Oliveira et al 2003 | Literature - checklist | |  |
| *Didelphis albiventris* | White-eared Opossum | Parque Nacional da Serra da Capivara | Piaui | -42.64065100000 | -8.83979400000 | Oliveira et al 2003, Gbif | Literature - checklist, observation | |  |
| *Eira barbara* | Tayra | Parque Nacional da Serra da Capivara | Piaui | -42.64065100000 | -8.83979400000 | Oliveira et al 2003 | Literature - checklist | |  |
| *Herpailurus yagouaroundi* | Jaguarundi | Parque Nacional da Serra da Capivara | Piaui | -42.64065100000 | -8.83979400000 | Species link | Collection | |  |
| *Kerodon rupestris* | Rock Cavy | Parque Nacional da Serra da Capivara | Piaui | -42.64065100000 | -8.83979400000 | Gbif | Observation | |  |
| *Leopardus emiliae* | Tiger cat | Parque Nacional da Serra da Capivara | Piaui | -42.64065100000 | -8.83979400000 | Penido et al 2017 | Camera trapping - checklist | |  |
| *Leopardus pardalis* | Ocelot | Parque Nacional da Serra da Capivara | Piaui | -42.64065100000 | -8.83979400000 | Penido et al 2017 | Camera trapping | |  |
| *Leopardus wiedii* | Margay | Parque Nacional da Serra da Capivara | Piaui | -42.64065100000 | -8.83979400000 | Oliveira et al 2003 | Literature - checklist | |  |
| *Mazama americana* | Red Brocket | Parque Nacional da Serra da Capivara | Piaui | -42.64065100000 | -8.83979400000 | Oliveira et al 2003 | Literature - checklist | |  |
| *Subulo gouazoubira* | Gray Brocket | Parque Nacional da Serra da Capivara | Piaui | -42.64065100000 | -8.83979400000 | Gbif | Observation | |  |
| *Myrmecophaga tridactyla* | Giant Anteater | Parque Nacional da Serra da Capivara | Piaui | -42.64065100000 | -8.83979400000 | Oliveira et al 2003 | Literature - checklist | |  |
| *Panthera onca* | Jaguar | Parque Nacional da Serra da Capivara | Piaui | -42.64065100000 | -8.83979400000 | Penido et al 2017,Feijo et al 2013, Gbif | Camera trapping - checklist, collection | |  |
| *Dicotyles tajacu* | Collaredy Pecary | Parque Nacional da Serra da Capivara | Piaui | -42.64065100000 | -8.83979400000 | Oliveira et al 2003 | Literature - checklist | |  |
| *Procyon cancrivorus* | Crab eating Raccoon | Parque Nacional da Serra da Capivara | Piaui | -42.64065100000 | -8.83979400000 | Oliveira et al 2003 | Literature - checklist | |  |
| *Puma concolor* | Puma | Parque Nacional da Serra da Capivara | Piaui | -42.64065100000 | -8.83979400000 | Penido et al 2017 | Camera trapping - checklist | |  |
| *Sapajus apella* | Black capped Capuchin | Parque Nacional da Serra da Capivara | Piaui | -42.64065100000 | -8.83979400000 | Oliveira et al 2003 | Literature - checklist | |  |
| *Sapajus libidinosus* | Bearded Capuchin | Parque Nacional da Serra da Capivara | Piaui | -42.64065100000 | -8.83979400000 | Gbif | Observation | |  |
| *Tamandua tetradactyla* | Southern Tamandua | Parque Nacional da Serra da Capivara | Piaui | -42.64065100000 | -8.83979400000 | Gbif | Observation | |  |
| *Tayassu* *pecari* | White-lipped Peccary | Parque Nacional da Serra da Capivara | Piaui | -42.64065100000 | -8.83979400000 | Oliveira et al 2003, Gbif | Literature - checklist, observation | |  |
| *Tolypeutes tricinctus* | Brazilian Three banded Armadillo | Parque Nacional da Serra da Capivara | Piaui | -42.64065100000 | -8.83979400000 | Gbif | Collection | |  |
| *Alouatta belzebul* | Red-handed-Howler monkey | Parque Nacional de Ubajara | Ceara | -40.89476700000 | -3.83465300000 | Guedes et al 2000, Oliverita dissertacao 2010 | Donation - checklist | |  |
| *Callithrix jacchus* | Common marmoset | Parque Nacional de Ubajara | Ceara | -40.89476700000 | -3.83465300000 | Guedes et al 2000, Oliverita dissertacao 2010 | Observation - checklist | |  |
| *Cerdocyon thous* | Crab eating fox | Parque Nacional de Ubajara | Ceara | -40.89476700000 | -3.83465300000 | Guedes et al 2000, Oliverita dissertacao 2010 | Interview - checklist | |  |
| *Coendou prehensilis* | Brazilian Porcupine | Parque Nacional de Ubajara | Ceara | -40.89476700000 | -3.83465300000 | Guedes et al 2000, Oliverita dissertacao 2010 | Interview - checklist | |  |
| *Conepatus semistriatus* | Striped hog nosed Skunk | Parque Nacional de Ubajara | Ceara | -40.89476700000 | -3.83465300000 | Guedes et al 2000, Oliverita dissertacao 2010 | Interview - checklist | |  |
| *Cuniculus paca* | Agouti | Parque Nacional de Ubajara | Ceara | -40.89476700000 | -3.83465300000 | Guedes et al 2000, Oliverita dissertacao 2010 | Interview - checklist | |  |
| *Dasyprocta prymnolopha* | red-orange rump agoutis | Parque Nacional de Ubajara | Ceara | -40.89476700000 | -3.83465300000 | Guedes et al 2000, Oliverita dissertacao 2010 | Observation - checklist | |  |
| *Dasypus novemcinctus* | Nine-banded Armadillo | Parque Nacional de Ubajara | Ceara | -40.89476700000 | -3.83465300000 | Guedes et al 2000, Oliverita dissertacao 2010 | Interview - checklist | |  |
| *Didelphis albiventris* | White-eared Opossum | Parque Nacional de Ubajara | Ceara | -40.89476700000 | -3.83465300000 | Guedes et al 2000, Oliverita dissertacao 2010 | Observation, donation - checklist | |  |
| *Eira barbara* | Tayra | Parque Nacional de Ubajara | Ceara | -40.89476700000 | -3.83465300000 | Guedes et al 2000, Oliverita dissertacao 2010 | Interview - checklist | |  |
| *Euphractus sexcinctus* | Yellow Armadillo | Parque Nacional de Ubajara | Ceara | -40.89476700000 | -3.83465300000 | Guedes et al 2000, Oliverita dissertacao 2010 | Donation - checklist | |  |
| *Galictis vittata* | Greater Grison | Parque Nacional de Ubajara | Ceara | -40.89476700000 | -3.83465300000 | Guedes et al 2000, Oliverita dissertacao 2010 | Observation - checklist | |  |
| *Herpailurus yagouaroundi* | Jaguarundi | Parque Nacional de Ubajara | Ceara | -40.89476700000 | -3.83465300000 | Guedes et al 2000, Oliverita dissertacao 2010 | Interview - checklist | |  |
| *Kerodon rupestris* | Rock Cavy | Parque Nacional de Ubajara | Ceara | -40.89476700000 | -3.83465300000 | Guedes et al 2000, Oliverita dissertacao 2010 | Observation - checklist | |  |
| *Leopardus emiliae* | Tiger cat | Parque Nacional de Ubajara | Ceara | -40.89476700000 | -3.83465300000 | Guedes et al 2000, Oliverita dissertacao 2010 | Donation - checklist | |  |
| *Mazama americana* | Red Brocket | Parque Nacional de Ubajara | Ceara | -40.89476700000 | -3.83465300000 | Guedes et al 2000, Oliverita dissertacao 2010 | Donation - checklist | |  |
| *Nasua nasua* | South American Coati | Parque Nacional de Ubajara | Ceara | -40.89476700000 | -3.83465300000 | Guedes et al 2000, Oliverita dissertacao 2010 | Interview - checklist | |  |
| *Procyon cancrivorus* | Crab eating Raccoon | Parque Nacional de Ubajara | Ceara | -40.89476700000 | -3.83465300000 | Guedes et al 2000, Oliverita dissertacao 2010 | Donation - checklist | |  |
| *Puma concolor* | Puma | Parque Nacional de Ubajara | Ceara | -40.89476700000 | -3.83465300000 | Guedes et al 2000, Oliverita dissertacao 2010 | Interview - checklist | |  |
| *Sapajus apella* | Black capped Capuchin | Parque Nacional de Ubajara | Ceara | -40.89476700000 | -3.83465300000 | Guedes et al 2000, Oliverita dissertacao 2010 | Observation - checklist | |  |
| *Sapajus libidinosus* | Bearded Capuchin | Parque Nacional de Ubajara | Ceara | -40.89476700000 | -3.83465300000 | Gbif | Observation | |  |
| *Tamandua tetradactyla* | Southern Tamandua | Parque Nacional de Ubajara | Ceara | -40.89476700000 | -3.83465300000 | Guedes et al 2000, Oliverita dissertacao 2010 | Donation - checklist | |  |
| *Callithrix jacchus* | Common marmoset | Parque Nacional do Catimbau | Pernambuco | -37.24754000000 | -8.57619700000 | Feijo et al 2013 | Interview | |  |
| *Cavia aperea* | Brazilian guinea pig | Parque Nacional do Catimbau | Pernambuco | -37.24754000000 | -8.57619700000 | Chaves et al 2020 | Interview - hunter | |  |
| *Cerdocyon thous* | Crab eating fox | Parque Nacional do Catimbau | Pernambuco | -37.24754000000 | -8.57619700000 | Alves et al 2020, Feijo et al 2013 | Camera trapping, footprints | |  |
| *Conepatus semistriatus* | Striped hog nosed Skunk | Parque Nacional do Catimbau | Pernambuco | -37.24754000000 | -8.57619700000 | Chaves et al 2020,Feijo et al 2013 | Interview - hunter | |  |
| *Dasyprocta prymnolopha* | red-orange rump agoutis | Parque Nacional do Catimbau | Pernambuco | -37.24754000000 | -8.57619700000 | Chaves et al 2020, Alves et al 2020 | Interview - hunter, camera trapping, direct evidenci - checklist | |  |
| *Dasypus novemcinctus* | Nine-banded Armadillo | Parque Nacional do Catimbau | Pernambuco | -37.24754000000 | -8.57619700000 | Chaves et al 2020, Alves et al 2020 | Interview - hunter, camera trapping, direct evidenci - checklist | |  |
| *Didelphis albiventris* | White-eared Opossum | Parque Nacional do Catimbau | Pernambuco | -37.24754000000 | -8.57619700000 | Chaves et al 2020 | Interview - hunter | |  |
| *Didelphis marsupialis* | Common Opossum | Parque Nacional do Catimbau | Pernambuco | -37.24754000000 | -8.57619700000 | Alves et al 2020 | Camera trapping, footprints | |  |
| *Euphractus sexcinctus* | Yellow Armadillo | Parque Nacional do Catimbau | Pernambuco | -37.24754000000 | -8.57619700000 | Chaves et al 2020, Alves et al 2020 | Interview - hunter, camera trapping, direct evidenci - checklist | |  |
| *Galea spixii* | Spix's Yellow-toothed Cavy | Parque Nacional do Catimbau | Pernambuco | -37.24754000000 | -8.57619700000 | Alves et al 2020 | Camera trapping, footprints | |  |
| *Kerodon rupestris* | Rock Cavy | Parque Nacional do Catimbau | Pernambuco | -37.24754000000 | -8.57619700000 | Chaves et al 2020 | Interview - hunter | |  |
| *Leopardus emiliae* | Tiger cat | Parque Nacional do Catimbau | Pernambuco | -37.24754000000 | -8.57619700000 | Chaves et al 2020, Alves et al 2020 | Interview - hunter, camera trapping, direct evidenci - checklist | |  |
| *Subulo gouazoubira* | Gray Brocket | Parque Nacional do Catimbau | Pernambuco | -37.24754000000 | -8.57619700000 | Chaves et al 2020, Alves et al 2020 | Interview - hunter, camera trapping, direct evidenci - checklist | |  |
| *Sylvilagus brasiliensis* | Tapeti | Parque Nacional do Catimbau | Pernambuco | -37.24754000000 | -8.57619700000 | Chaves et al 2020, Alves et al 2020 | Interview - hunter, camera trapping, direct evidenci - checklist | |  |
| *Tamandua tetradactyla* | Southern Tamandua | Parque Nacional do Catimbau | Pernambuco | -37.24754000000 | -8.57619700000 | Chaves et al 2020 | Interview - hunter | |  |
| *Callithrix jacchus* | Common marmoset | Parque Nacional Serra das Confusoes | Piaui | -43.40690300000 | -8.92418500000 | Henrique et al 2007 | Interview, camera trapping, indirect evidences - checklist | |  |
| *Cerdocyon thous* | Crab eating fox | Parque Nacional Serra das Confusoes | Piaui | -43.40690300000 | -8.92418500000 | Henrique et al 2007 | Interview, camera trapping, indirect evidences - checklist | |  |
| *Coendou prehensilis* | Brazilian Porcupine | Parque Nacional Serra das Confusoes | Piaui | -43.40690300000 | -8.92418500000 | Henrique et al 2007 | Interview, camera trapping, indirect evidences - checklist | |  |
| *Cuniculus paca* | Agouti | Parque Nacional Serra das Confusoes | Piaui | -43.40690300000 | -8.92418500000 | Henrique et al 2007 | Interview, camera trapping, indirect evidences - checklist | |  |
| *Eira barbara* | Tayra | Parque Nacional Serra das Confusoes | Piaui | -43.40690300000 | -8.92418500000 | Henrique et al 2007 | Interview, camera trapping, indirect evidences - checklist | |  |
| *Herpailurus yagouaroundi* | Jaguarundi | Parque Nacional Serra das Confusoes | Piaui | -43.40690300000 | -8.92418500000 | Henrique et al 2007 | Interview, camera trapping, indirect evidences - checklist | |  |
| *Kerodon rupestris* | Rock Cavy | Parque Nacional Serra das Confusoes | Piaui | -43.40690300000 | -8.92418500000 | Henrique et al 2007 | Interview, camera trapping, indirect evidences - checklist | |  |
| *Leopardus emiliae* | Tiger cat | Parque Nacional Serra das Confusoes | Piaui | -43.40690300000 | -8.92418500000 | Henrique et al 2007 | Interview, camera trapping, indirect evidences - checklist | |  |
| *Leopardus pardalis* | Ocelot | Parque Nacional Serra das Confusoes | Piaui | -43.40690300000 | -8.92418500000 | Henrique et al 2007 | Interview, camera trapping, indirect evidences - checklist | |  |
| *Leopardus wiedii* | Margay | Parque Nacional Serra das Confusoes | Piaui | -43.40690300000 | -8.92418500000 | Henrique et al 2007 | Interview, camera trapping, indirect evidences - checklist | |  |
| *Myrmecophaga tridactyla* | Giant Anteater | Parque Nacional Serra das Confusoes | Piaui | -43.40690300000 | -8.92418500000 | Henrique et al 2007 | Interview, camera trapping, indirect evidences - checklist | |  |
| *Nasua Nasua* | South American Coati | Parque Nacional Serra das Confusoes | Piaui | -43.40690300000 | -8.92418500000 | Henrique et al 2007 | Interview, camera trapping, indirect evidences - checklist | |  |
| *Panthera onca* | Jaguar | Parque Nacional Serra das Confusoes | Piaui | -43.40690300000 | -8.92418500000 | Henrique et al 2007 | Interview, camera trapping, indirect evidences - checklist | |  |
| *Dicotyles tajacu* | Collaredy Pecary | Parque Nacional Serra das Confusoes | Piaui | -43.40690300000 | -8.92418500000 | Henrique et al 2007 | Interview, camera trapping, indirect evidences - checklist | |  |
| *Priodontes maximus* | Giant Armadillo | Parque Nacional Serra das Confusoes | Piaui | -43.40690300000 | -8.92418500000 | Henrique et al 2007 | Interview, camera trapping, indirect evidences - checklist | |  |
| *Procyon cancrivorus* | Crab eating Raccoon | Parque Nacional Serra das Confusoes | Piaui | -43.40690300000 | -8.92418500000 | Henrique et al 2007 | Interview, camera trapping, indirect evidences - checklist | |  |
| *Puma concolor* | Puma | Parque Nacional Serra das Confusoes | Piaui | -43.40690300000 | -8.92418500000 | Henrique et al 2007 | Interview, camera trapping, indirect evidences - checklist | |  |
| *Sapajus apella* | Black capped Capuchin | Parque Nacional Serra das Confusoes | Piaui | -43.40690300000 | -8.92418500000 | Henrique et al 2007 | Interview, camera trapping, indirect evidences - checklist | |  |
| *Tamandua tetradactyla* | Southern Tamandua | Parque Nacional Serra das Confusoes | Piaui | -43.40690300000 | -8.92418500000 | Henrique et al 2007 | Interview, camera trapping, indirect evidences - checklist | |  |
| *Tolypeutes tricinctus* | Brazilian Three banded Armadillo | Parque Nacional Serra das Confusoes | Piaui | -43.40690300000 | -8.92418500000 | Henrique et al 2007 | Interview, camera trapping, indirect evidences - checklist | |  |
| *Cerdocyon thous* | Crab eating fox | Patos | Paraiba | -37.20164300000 | -7.02936800000 | Feijo et al 2013 | Collection | |  |
| *Conepatus semistriatus* | Striped hog nosed Skunk | Patos | Paraiba | -37.20164300000 | -7.02936800000 | Feijo et al 2013 | Interview | |  |
| *Euphractus sexcinctus* | Yellow Armadillo | Patos | Paraiba | -37.20164300000 | -7.02936800000 | Feijo et al 2013 | Interview | |  |
| *Herpailurus yagouaroundi* | Jaguarundi | Patos | Paraiba | -37.20164300000 | -7.02936800000 | Feijo et al 2013 | Collection | |  |
| *Subulo gouazoubira* | Gray Brocket | Patos | Paraiba | -37.20164300000 | -7.02936800000 | Feijo et al 2013 | Interview | |  |
| *Procyon cancrivorus* | Crab eating Raccoon | Patos | Paraiba | -37.20164300000 | -7.02936800000 | Feijo et al 2013 | Interview | |  |
| *Sapajus libidinosus* | Bearded Capuchin | Patos | Paraiba | -37.20164300000 | -7.02936800000 | Feijo et al 2013 | Interview | |  |
| *Sylvilagus brasiliensis* | Tapeti | Patos | Paraiba | -37.20164300000 | -7.02936800000 | Feijo et al 2013 | Interview | |  |
| *Tamandua tetradactyla* | Southern Tamandua | Patos | Paraiba | -37.20164300000 | -7.02936800000 | Feijo et al 2013 | Interview | |  |
| *Callithrix jacchus* | Common marmoset | Paulista | Paraiba | -37.62416700000 | -6.59944400000 | Mourao et al 2006 | Interview - hunter | |  |
| *Cerdocyon thous* | Crab eating fox | Paulista | Paraiba | -37.62416700000 | -6.59944400000 | Mourao et al 2006 | Interview - hunter | |  |
| *Dasypus novemcinctus* | Nine-banded Armadillo | Paulista | Paraiba | -37.62416700000 | -6.59944400000 | Mourao et al 2006 | Interview - hunter | |  |
| *Euphractus sexcinctus* | Yellow Armadillo | Paulista | Paraiba | -37.62416700000 | -6.59944400000 | Mourao et al 2006 | Interview - hunter | |  |
| *Galea spixii* | Spix's Yellow-toothed Cavy | Paulista | Paraiba | -37.62416700000 | -6.59944400000 | Mourao et al 2006 | Interview - hunter | |  |
| *Galictis cuja* | Lesser Grison | Paulista | Paraiba | -37.62416700000 | -6.59944400000 | Mourao et al 2006 | Interview - hunter | |  |
| *Galictis vittata* | Greater Grison | Paulista | Paraiba | -37.62416700000 | -6.59944400000 | Mourao et al 2006 | Interview - hunter | |  |
| *Herpailurus yagouaroundi* | Jaguarundi | Paulista | Paraiba | -37.62416700000 | -6.59944400000 | Mourao et al 2006 | Interview - hunter | |  |
| *Kerodon rupestris* | Rock Cavy | Paulista | Paraiba | -37.62416700000 | -6.59944400000 | Mourao et al 2006 | Interview - hunter | |  |
| *Leopardus wiedii* | Margay | Paulista | Paraiba | -37.62416700000 | -6.59944400000 | Mourao et al 2006 | Interview - hunter | |  |
| *Myrmecophaga tridactyla* | Giant Anteater | Paulista | Paraiba | -37.62416700000 | -6.59944400000 | Mourao et al 2006 | Interview - hunter | |  |
| *Dicotyles tajacu* | Collaredy Pecary | Paulista | Paraiba | -37.62416700000 | -6.59944400000 | Mourao et al 2006 | Interview - hunter | |  |
| *Procyon cancrivorus* | Crab eating Raccoon | Paulista | Paraiba | -37.62416700000 | -6.59944400000 | Mourao et al 2006 | Interview - hunter | |  |
| *Sapajus apella* | Black capped Capuchin | Paulista | Paraiba | -37.62416700000 | -6.59944400000 | Mourao et al 2006 | Interview - hunter | |  |
| *Tamandua tetradactyla* | Southern Tamandua | Paulista | Paraiba | -37.62416700000 | -6.59944400000 | Mourao et al 2006 | Interview - hunter | |  |
| *Callithrix jacchus* | Common marmoset | Paulo Afonso | Bahia | -38.20829200000 | -9.38656900000 | Bezerra et al 2014 | Direct evidence, trails - checklist | |  |
| *Cerdocyon thous* | Crab eating fox | Paulo Afonso | Bahia | -38.20829200000 | -9.38656900000 | Bezerra et al 2014 | Direct evidence, trails - checklist | |  |
| *Euphractus sexcinctus* | Yellow Armadillo | Paulo Afonso | Bahia | -38.20829200000 | -9.38656900000 | Gbif | Observation | |  |
| *Galea spixii* | Spix's Yellow-toothed Cavy | Paulo Afonso | Bahia | -38.20829200000 | -9.38656900000 | Bezerra et al 2014 | Direct evidence, trails - checklist | |  |
| *Procyon cancrivorus* | Crab eating Raccoon | Paulo Afonso | Bahia | -38.20829200000 | -9.38656900000 | Bezerra et al 2014 | Direct evidence, trails - checklist | |  |
| *Cavia aperea* | Brazilian guinea pig | Pocinhos | Paraiba | -36.03402100000 | -7.09275200000 | Alves et al 2009 | Interview - hunter | |  |
| *Cerdocyon thous* | Crab eating fox | Pocinhos | Paraiba | -36.03402100000 | -7.09275200000 | Alves et al 2009, Confessor et al 2009 | Interview - hunter | |  |
| *Conepatus semistriatus* | Striped hog nosed Skunk | Pocinhos | Paraiba | -36.03402100000 | -7.09275200000 | Alves et al 2009, Mendonca et al 2016, Feijo et al 2013 | Interview - hunter, collection | |  |
| *Dasypus novemcinctus* | Nine-banded Armadillo | Pocinhos | Paraiba | -36.03402100000 | -7.09275200000 | Alves et al 2009, Mendonca et al 2016 | Interview - hunter | |  |
| *Euphractus sexcinctus* | Yellow Armadillo | Pocinhos | Paraiba | -36.03402100000 | -7.09275200000 | Alves et al 2009, Confessor et al 2009, Mendonca et al 2014, Feijo et al 2013 | Interview - hunter, collection | |  |
| *Galea spixii* | Spix's Yellow-toothed Cavy | Pocinhos | Paraiba | -36.03402100000 | -7.09275200000 | Alves et al 2009, Mendonca et al 2016 | Interview - hunter | |  |
| *Galictis vittata* | Greater Grison | Pocinhos | Paraiba | -36.03402100000 | -7.09275200000 | Alves et al 2009 | Interview - hunter | |  |
| *Herpailurus yagouaroundi* | Jaguarundi | Pocinhos | Paraiba | -36.03402100000 | -7.09275200000 | Alves et al 2009 | Interview - hunter | |  |
| *Kerodon rupestris* | Rock Cavy | Pocinhos | Paraiba | -36.03402100000 | -7.09275200000 | Alves et al 2009, Mendonca et al 2016 | Interview - hunter | |  |
| *Leopardus emiliae* | Tiger cat | Pocinhos | Paraiba | -36.03402100000 | -7.09275200000 | Alves et al 2009, Feijo et al 2013 | Interview - hunter, collection | |  |
| *Tamandua tetradactyla* | Southern Tamandua | Pocinhos | Paraiba | -36.03402100000 | -7.09275200000 | Alves et al 2009, Mendonca et al 2016 | Interview - hunter | |  |
| *Alouatta belzebul* | Red-handed-Howler monkey | Quixada | Ceara | -39.11391100000 | -4.90269700000 | Feijo et al 2013 | Interview | |  |
| *Conepatus semistriatus* | Striped hog nosed Skunk | Quixada | Ceara | -39.11391100000 | -4.90269700000 | Feijo et al 2013 | Interview | |  |
| *Galea spixii* | Spix's Yellow-toothed Cavy | Quixada | Ceara | -39.11391100000 | -4.90269700000 | Gbif | Collection | |  |
| *Galictis cuja* | Lesser Grison | Quixada | Ceara | -39.11391100000 | -4.90269700000 | Feijo et al 2013 | Interview | |  |
| *Kerodon rupestris* | Rock Cavy | Quixada | Ceara | -39.11391100000 | -4.90269700000 | Gbif | Collection | |  |
| *Cabassous tatouay* | Greater Naked-tailed Armadillo | Reserva Natural Serra das Almas | Ceara | -40.25 | -5.25 | Feijo et al 2013 | Collection | |  |
| *Callithrix jacchus* | Common marmoset | Reserva Natural Serra das Almas | Ceara | -40.25 | -5.25 | Dias et al 2017, Cruz et al 2005 | Indirect evidence, direct evidence, camera trapping - checklist | |  |
| *Cerdocyon thous* | Crab eating fox | Reserva Natural Serra das Almas | Ceara | -40.25 | -5.25 | Dias et al 2017, Cruz et al 2005 | Indirect evidence, direct evidence, camera trapping - checklist | |  |
| *Conepatus semistriatus* | Striped hog nosed Skunk | Reserva Natural Serra das Almas | Ceara | -40.25 | -5.25 | Dias et al 2017 | Indirect evidence, direct evidence, camera trapping - checklist | |  |
| *Cuniculus paca* | Agouti | Reserva Natural Serra das Almas | Ceara | -40.25 | -5.25 | Dias et al 2017 | Indirect evidence, direct evidence, camera trapping - checklist | |  |
| *Dasyprocta prymnolopha* | red-orange rump agoutis | Reserva Natural Serra das Almas | Ceara | -40.25 | -5.25 | Dias et al 2017 | Indirect evidence, direct evidence, camera trapping - checklist | |  |
| *Dasypus novemcinctus* | Nine-banded Armadillo | Reserva Natural Serra das Almas | Ceara | -40.25 | -5.25 | Dias et al 2017, Cruz et al 2005 | Indirect evidence, direct evidence, camera trapping - checklist | |  |
| *Dasypus septemcinctus* | Brazilian Lesser long-nosed Armadillo | Reserva Natural Serra das Almas | Ceara | -40.25 | -5.25 | Dias et al 2017 | Indirect evidence, direct evidence, camera trapping - checklist | |  |
| *Didelphis albiventris* | White-eared Opossum | Reserva Natural Serra das Almas | Ceara | -40.25 | -5.25 | Dias et al 2017 | Indirect evidence, direct evidence, camera trapping - checklist | |  |
| *Eira barbara* | Tayra | Reserva Natural Serra das Almas | Ceara | -40.25 | -5.25 | Dias et al 2017 | Indirect evidence, direct evidence, camera trapping - checklist | |  |
| *Euphractus sexcinctus* | Yellow Armadillo | Reserva Natural Serra das Almas | Ceara | -40.25 | -5.25 | Dias et al 2017, Cruz et al 2005 | Indirect evidence, direct evidence, camera trapping - checklist | |  |
| *Galea spixii* | Spix's Yellow-toothed Cavy | Reserva Natural Serra das Almas | Ceara | -40.25 | -5.25 | Dias et al 2017, Cruz et al 2005 | Indirect evidence, direct evidence, camera trapping - checklist | |  |
| *Galictis cuja* | Lesser Grison | Reserva Natural Serra das Almas | Ceara | -40.25 | -5.25 | Dias et al 2017 | Indirect evidence, direct evidence, camera trapping - checklist | |  |
| *Herpailurus yagouaroundi* | Jaguarundi | Reserva Natural Serra das Almas | Ceara | -40.25 | -5.25 | Dias et al 2017 | Indirect evidence, direct evidence, camera trapping - checklist | |  |
| *Kerodon rupestris* | Rock Cavy | Reserva Natural Serra das Almas | Ceara | -40.25 | -5.25 | Dias et al 2017 | Indirect evidence, direct evidence, camera trapping - checklist | |  |
| *Leopardus emiliae* | Tiger cat | Reserva Natural Serra das Almas | Ceara | -40.25 | -5.25 | Dias et al 2017, Feijo et al 2013 | Indirect evidence, direct evidence, camera trapping - checklist | |  |
| *Leopardus pardalis* | Ocelot | Reserva Natural Serra das Almas | Ceara | -40.25 | -5.25 | Dias et al 2017 | Indirect evidence, direct evidence, camera trapping - checklist | |  |
| *Subulo gouazoubira* | Gray Brocket | Reserva Natural Serra das Almas | Ceara | -40.25 | -5.25 | Dias et al 2017, Cruz et al 2005 | Indirect evidence, direct evidence, camera trapping - checklist | |  |
| *Dicotyles tajacu* | Collaredy Pecary | Reserva Natural Serra das Almas | Ceara | -40.25 | -5.25 | Dias et al 2017 | Indirect evidence, direct evidence, camera trapping - checklist | |  |
| *Procyon cancrivorus* | Crab eating Raccoon | Reserva Natural Serra das Almas | Ceara | -40.25 | -5.25 | Dias et al 2017, Cruz et al 2005 | Indirect evidence, direct evidence, camera trapping - checklist | |  |
| *Puma concolor* | Puma | Reserva Natural Serra das Almas | Ceara | -40.25 | -5.25 | Dias et al 2017, Cruz et al 2005 | Indirect evidence, direct evidence, camera trapping - checklist | |  |
| *Sapajus apella* | Black capped Capuchin | Reserva Natural Serra das Almas | Ceara | -40.25 | -5.25 | Cruz et al 2005 | Indirect evidence, tracks, interview - checklist | |  |
| *Sapajus libidinosus* | Bearded Capuchin | Reserva Natural Serra das Almas | Ceara | -40.25 | -5.25 | Dias et al 2017 | Indirect evidence, direct evidence, camera trapping - checklist | |  |
| *Tamandua tetradactyla* | Southern Tamandua | Reserva Natural Serra das Almas | Ceara | -40.25 | -5.25 | Dias et al 2017, Feijo et al 2013 | Indirect evidence, direct evidence, camera trapping - checklist | |  |
| *Callithrix jacchus* | Common marmoset | RPPN Cantidiano Valgueiro | Pernambuco | -38.51527800000 | -8.44888900000 | Cruz et al 2005 | Indirect evidence, tracks, interview - checklist | |  |
| *Cerdocyon thous* | Crab eating fox | RPPN Cantidiano Valgueiro | Pernambuco | -38.51527800000 | -8.44888900000 | Cruz et al 2005 | Indirect evidence, tracks, interview - checklist | |  |
| *Conepatus semistriatus* | Striped hog nosed Skunk | RPPN Cantidiano Valgueiro | Pernambuco | -38.51527800000 | -8.44888900000 | Cruz et al 2005 | Indirect evidence, tracks, interview - checklist | |  |
| *Euphractus sexcinctus* | Yellow Armadillo | RPPN Cantidiano Valgueiro | Pernambuco | -38.51527800000 | -8.44888900000 | Cruz et al 2005 | Indirect evidence, tracks, interview - checklist | |  |
| *Galea spixii* | Spix's Yellow-toothed Cavy | RPPN Cantidiano Valgueiro | Pernambuco | -38.51527800000 | -8.44888900000 | Cruz et al 2005 | Indirect evidence, tracks, interview - checklist | |  |
| *Kerodon rupestris* | Rock Cavy | RPPN Cantidiano Valgueiro | Pernambuco | -38.51527800000 | -8.44888900000 | Cruz et al 2005 | Indirect evidence, tracks, interview - checklist | |  |
| *Leopardus emiliae* | Tiger cat | RPPN Cantidiano Valgueiro | Pernambuco | -38.51527800000 | -8.44888900000 | Cruz et al 2005 | Indirect evidence, tracks, interview - checklist | |  |
| *Subulo gouazoubira* | Gray Brocket | RPPN Cantidiano Valgueiro | Pernambuco | -38.51527800000 | -8.44888900000 | Cruz et al 2005 | Indirect evidence, tracks, interview - checklist | |  |
| *Tamandua tetradactyla* | Southern Tamandua | RPPN Cantidiano Valgueiro | Pernambuco | -38.51527800000 | -8.44888900000 | Cruz et al 2005 | Indirect evidence, tracks, interview - checklist | |  |
| *Cerdocyon thous* | Crab eating fox | RPPN Fazenda Almas | Paraíba | -36.88083300000 | -7.47083300000 | Feijó et al 2013, Alves tese 2020 | Collection, camera trapping - checklist | |  |
| *Conepatus semistriatus* | Striped hog nosed Skunk | RPPN Fazenda Almas | Paraíba | -36.88083300000 | -7.47083300000 | Feijó et al 2013, Alves tese 2020 | Collection, camera trapping - checklist | |  |
| *Didelphis albiventris* | White-eared Opossum | RPPN Fazenda Almas | Paraíba | -36.88083300000 | -7.47083300000 | Alves tese 2020 | Camera trapping - checklist | |  |
| *Euphractus sexcinctus* | Yellow Armadillo | RPPN Fazenda Almas | Paraíba | -36.88083300000 | -7.47083300000 | Alves tese 2020 | Camera trapping - checklist | |  |
| *Galea spixii* | Spix's Yellow-toothed Cavy | RPPN Fazenda Almas | Paraíba | -36.88083300000 | -7.47083300000 | Alves tese 2020 | Camera trapping - checklist | |  |
| *Herpailurus yagouaroundi* | Jaguarundi | RPPN Fazenda Almas | Paraíba | -36.88083300000 | -7.47083300000 | Feijó et al 2013 | Collection | |  |
| *Procyon cancrivorus* | Crab eating Raccoon | RPPN Fazenda Almas | Paraíba | -36.88083300000 | -7.47083300000 | Feijó et al 2013, Alves tese 2020 | Collection, camera trapping - checklist | |  |
| *Puma concolor* |  | RPPN Fazenda Almas | Paraíba | -36.88083300000 | -7.47083300000 | Projeto Nexus 2022 | Camera trapping | |  |
| *Tamandua tetradactyla* | Southern Tamandua | RPPN Fazenda Almas | Paraíba | -36.88083300000 | -7.47083300000 | Alves tese 2020 | Camera trapping - checklist | |  |
| *Callithrix jacchus* | Common marmoset | RPPN Mauricio Dantas | Pernambuco | -38.20611100000 | -8.28916700000 | Cruz et al 2005 | Indirect evidence, tracks, interview - checklist | |  |
| *Cerdocyon thous* | Crab eating fox | RPPN Mauricio Dantas | Pernambuco | -38.20611100000 | -8.28916700000 | Cruz et al 2005 | Indirect evidence, tracks, interview - checklist | |  |
| *Euphractus sexcinctus* | Yellow Armadillo | RPPN Mauricio Dantas | Pernambuco | -38.20611100000 | -8.28916700000 | Cruz et al 2005 | Indirect evidence, tracks, interview - checklist | |  |
| *Leopardus emiliae* | Tiger cat | RPPN Mauricio Dantas | Pernambuco | -38.20611100000 | -8.28916700000 | Cruz et al 2005 | Indirect evidence, tracks, interview - checklist | |  |
| *Subulo gouazoubira* | Gray Brocket | RPPN Mauricio Dantas | Pernambuco | -38.20611100000 | -8.28916700000 | Cruz et al 2005 | Indirect evidence, tracks, interview - checklist | |  |
| *Procyon cancrivorus* | Crab eating Raccoon | RPPN Mauricio Dantas | Pernambuco | -38.20611100000 | -8.28916700000 | Cruz et al 2005 | Indirect evidence, tracks, interview - checklist | |  |
| *Callithrix jacchus* | Common marmoset | Salambaia | Paraíba | -36.280278 | '-7.368611 | Alves tese 2020 | Camera trapping - checklist | |  |
| *Cerdocyon thous* | Crab eating fox | Salambaia | Paraíba | -36.280278 | '-7.368611 | Alves tese 2020 | Camera trapping - checklist | |  |
| *Conepatus semistriatus* | Striped hog nosed Skunk | Salambaia | Paraíba | -36.280278 | '-7.368611 | Alves tese 2020 | Camera trapping - checklist | |  |
| *Didelphis albiventris* | White-eared Opossum | Salambaia | Paraíba | -36.280278 | '-7.368611 | Alves tese 2020 | Camera trapping - checklist | |  |
| *Euphractus sexcinctus* | Yellow Armadillo | Salambaia | Paraíba | -36.280278 | '-7.368611 | Alves tese 2020 | Camera trapping - checklist | |  |
| *Galea spixii* | Spix's Yellow-toothed Cavy | Salambaia | Paraíba | -36.280278 | '-7.368611 | Alves tese 2020 | Camera trapping - checklist | |  |
| *Leopardus emiliae* | Tiger cat | Salambaia | Paraíba | -36.280278 | '-7.368611 | Alves tese 2020 | Camera trapping - checklist | |  |
| *Procyon cancrivorus* | Crab eating Raccoon | Salambaia | Paraíba | -36.280278 | '-7.368611 | Alves tese 2020 | Camera trapping - checklist | |  |
| *Tamandua tetradactyla* | Southern Tamandua | Salambaia | Paraíba | -36.280278 | '-7.368611 | Alves tese 2020 | Camera trapping - checklist | |  |
| *Callithrix jacchus* | Common marmoset | Santa Quiteria | Ceara | -40.12008500000 | -4.45748800000 | Feijo et al 2013 | Interview | |  |
| *Cerdocyon thous* | Crab eating fox | Santa Quiteria | Ceara | -40.12008500000 | -4.45748800000 | Feijo et al 2013 | Interview | |  |
| *Euphractus sexcinctus* | Yellow Armadillo | Santa Quiteria | Ceara | -40.12008500000 | -4.45748800000 | Feijo et al 2013 | Museum collection | |  |
| *Leopardus emiliae* | Tiger cat | Santa Quiteria | Ceara | -40.12008500000 | -4.45748800000 | Feijo et al 2013 | Interview | |  |
| *Dicotyles tajacu* | Collaredy Pecary | Santa Quiteria | Ceara | -40.12008500000 | -4.45748800000 | Feijo et al 2013 | Museum collection | |  |
| *Procyon cancrivorus* | Crab eating Raccoon | Santa Quiteria | Ceara | -40.12008500000 | -4.45748800000 | Feijo et al 2013 | Interview | |  |
| *Alouatta belzebul* | Red-handed-Howler monkey | Sao Benedito | Ceara | -40.82378900000 | -4.05535500000 | Feijo et al 2013 | Museum collection | |  |
| *Callithrix jacchus* | Common marmoset | Sao Benedito | Ceara | -40.82378900000 | -4.05535500000 | Feijo et al 2013 | Museum collection | |  |
| *Cerdocyon thous* | Crab eating fox | Sao Benedito | Ceara | -40.82378900000 | -4.05535500000 | Feijo et al 2013 | Museum collection | |  |
| *Conepatus semistriatus* | Striped hog nosed Skunk | Sao Benedito | Ceara | -40.82378900000 | -4.05535500000 | Feijo et al 2013 | Museum collection | |  |
| *Dasyprocta prymnolopha* | red-orange rump agoutis | Sao Benedito | Ceara | -40.82378900000 | -4.05535500000 | Feijo et al 2013 | Museum collection | |  |
| *Galictis cuja* | Lesser Grison | Sao Benedito | Ceara | -40.82378900000 | -4.05535500000 | Feijo et al 2013 | Museum collection | |  |
| *Leopardus emiliae* | Tiger cat | Sao Benedito | Ceara | -40.82378900000 | -4.05535500000 | Feijo et al 2013 | Museum collection | |  |
| *Nasua nasua* | South American Coati | Sao Benedito | Ceara | -40.82378900000 | -4.05535500000 | Feijo et al 2013 | Museum collection | |  |
| *Dicotyles tajacu* | Collaredy Pecary | Sao Benedito | Ceara | -40.82378900000 | -4.05535500000 | Feijo et al 2013 | Museum collection | |  |
| *Sapajus libidinosus* | Bearded Capuchin | Sao Benedito | Ceara | -40.82378900000 | -4.05535500000 | Feijo et al 2013 | Museum collection | |  |
| *Cavia aperea* | Brazilian guinea pig | Sao Fernando | Rio Grande do Norte | -37.19004400000 | -6.38504600000 | Barbosa et al 2016 | Interview - hunter | |  |
| *Cerdocyon thous* | Crab eating fox | Sao Fernando | Rio Grande do Norte | -37.19004400000 | -6.38504600000 | Barbosa et al 2016 | Interview - hunter | |  |
| *Conepatus semistriatus* | Striped hog nosed Skunk | Sao Fernando | Rio Grande do Norte | -37.19004400000 | -6.38504600000 | Barbosa et al 2016 | Interview - hunter | |  |
| *Dasyprocta prymnolopha* | red-orange rump agoutis | Sao Fernando | Rio Grande do Norte | -37.19004400000 | -6.38504600000 | Barbosa et al 2016 | Interview - hunter | |  |
| *Dasypus novemcinctus* | Nine-banded Armadillo | Sao Fernando | Rio Grande do Norte | -37.19004400000 | -6.38504600000 | Barbosa et al 2016 | Interview - hunter | |  |
| *Didelphis marsupialis* | Common Opossum | Sao Fernando | Rio Grande do Norte | -37.19004400000 | -6.38504600000 | Barbosa et al 2016 | Interview - hunter | |  |
| *Euphractus sexcinctus* | Yellow Armadillo | Sao Fernando | Rio Grande do Norte | -37.19004400000 | -6.38504600000 | Barbosa et al 2016 | Interview - hunter | |  |
| *Galea spixii* | Spix's Yellow-toothed Cavy | Sao Fernando | Rio Grande do Norte | -37.19004400000 | -6.38504600000 | Barbosa et al 2016 | Interview - hunter | |  |
| *Galictis vittata* | Greater Grison | Sao Fernando | Rio Grande do Norte | -37.19004400000 | -6.38504600000 | Barbosa et al 2016 | Interview - hunter | |  |
| *Herpailurus yagouaroundi* | Jaguarundi | Sao Fernando | Rio Grande do Norte | -37.19004400000 | -6.38504600000 | Barbosa et al 2016 | Interview - hunter | |  |
| *Kerodon rupestris* | Rock Cavy | Sao Fernando | Rio Grande do Norte | -37.19004400000 | -6.38504600000 | Barbosa et al 2016 | Interview - hunter | |  |
| *Leopardus emiliae* | Tiger cat | Sao Fernando | Rio Grande do Norte | -37.19004400000 | -6.38504600000 | Barbosa et al 2016 | Interview - hunter | |  |
| *Leopardus pardalis* | Ocelot | Sao Fernando | Rio Grande do Norte | -37.19004400000 | -6.38504600000 | Barbosa et al 2016 | Interview - hunter | |  |
| *Leopardus wiedii* | Margay | Sao Fernando | Rio Grande do Norte | -37.19004400000 | -6.38504600000 | Barbosa et al 2016 | Interview - hunter | |  |
| *Mazama americana* | Red Brocket | Sao Fernando | Rio Grande do Norte | -37.19004400000 | -6.38504600000 | Barbosa et al 2016 | Interview - hunter | |  |
| *Subulo gouazoubira* | Gray Brocket | Sao Fernando | Rio Grande do Norte | -37.19004400000 | -6.38504600000 | Barbosa et al 2016 | Interview - hunter | |  |
| *Procyon cancrivorus* | Crab eating Raccoon | Sao Fernando | Rio Grande do Norte | -37.19004400000 | -6.38504600000 | Barbosa et al 2016 | Interview - hunter | |  |
| *Tamandua tetradactyla* | Southern Tamandua | Sao Fernando | Rio Grande do Norte | -37.19004400000 | -6.38504600000 | Barbosa et al 2016 | Interview - hunter | |  |
| *Cerdocyon thous* | Crab eating fox | Sao Joao do Cariri,Cabaceiras | Paraiba | -36.52294600000 | -7.39875400000 | Alves et al 2012, Feijó et al 2013, Alves tese 2020 | Interview, camera trapping - checklist | |  |
| *Conepatus semistriatus* | Striped hog nosed Skunk | Sao Joao do Cariri,Cabaceiras | Paraiba | -36.52294600000 | -7.39875400000 | Alves et al 2012, Feijó et al 2013, Alves tese 2020 | Interview, camera trapping - checklist | |  |
| *Dasypus novemcinctus* | Nine-banded Armadillo | Sao Joao do Cariri,Cabaceiras | Paraiba | -36.52294600000 | -7.39875400000 | Alves et al 2012 | Interview | |  |
| *Didelphis albiventris* | White-eared Opossum | Sao Joao do Cariri,Cabaceiras | Paraíba | -36.52294600000 | -7.39875400000 | Alves tese 2020 | Camera trapping - checklist | |  |
| *Euphractus sexcinctus* | Yellow Armadillo | Sao Joao do Cariri,Cabaceiras | Paraiba | -36.52294600000 | -7.39875400000 | Alves et al 2012, Feijó et al 2013, Alves tese 2020 | Interview, camera trapping - checklist | |  |
| *Galea spixii* | Spix's Yellow-toothed Cavy | Sao Joao do Cariri,Cabaceiras | Paraiba | -36.52294600000 | -7.39875400000 | Alves et al 2012, Alves tese 2020 | Interview, camera trapping - checklist | |  |
| *Galictis cuja* | Lesser Grison | Sao Joao do Cariri,Cabaceiras | Paraiba | -36.52294600000 | -7.39875400000 | Alves et al 2012 | Interview | |  |
| *Herpailurus yagouaroundi* | Jaguarundi | Sao Joao do Cariri,Cabaceiras | Paraiba | -36.52294600000 | -7.39875400000 | Alves et al 2012 | Interview | |  |
| *Kerodon rupestris* | Rock Cavy | Sao Joao do Cariri,Cabaceiras | Paraiba | -36.52294600000 | -7.39875400000 | Alves et al 2012, Feijo et al 2013 | Interview | |  |
| *Leopardus emiliae* | Tiger cat | Sao Joao do Cariri,Cabaceiras | Paraiba | -36.52294600000 | -7.39875400000 | Alves et al 2012, Feijo et al 2013 | Interview | |  |
| *Subulo gouazoubira* | Gray Brocket | Sao Joao do Cariri,Cabaceiras | Paraiba | -36.52294600000 | -7.39875400000 | Alves et al 2012 | Interview | |  |
| *Procyon cancrivorus* | Crab eating Raccoon | Sao Joao do Cariri,Cabaceiras | Paraiba | -36.52294600000 | -7.39875400000 | Feijo et al 2013, Alves tese 2020 | Museum collection, camera trapping - checklist | |  |
| *Tamandua tetradactyla* | Southern Tamandua | Sao Joao do Cariri,Cabaceiras | Paraiba | -36.52294600000 | -7.39875400000 | Alves et al 2012, Alves tese 2020 | Interview, camera trapping - checklist | |  |
| *Callithrix jacchus* | Common marmoset | Sao Joao do Piaui | Piaui | -42.09122200000 | -8.26691700000 | Dellcielos et al 2016 | Camera trappings, indirect evidence - checklist | |  |
| *Cerdocyon thous* | Crab eating fox | Sao Joao do Piaui | Piaui | -42.09122200000 | -8.26691700000 | Dellcielos et al 2016 | Camera trappings, indirect evidence - checklist | |  |
| *Conepatus semistriatus* | Striped hog nosed Skunk | Sao Joao do Piaui | Piaui | -42.09122200000 | -8.26691700000 | Dellcielos et al 2016 | Camera trappings, indirect evidence - checklist | |  |
| *Dasyprocta prymnolopha* | red-orange rump agoutis | Sao Joao do Piaui | Piaui | -42.09122200000 | -8.26691700000 | Dellcielos et al 2016 | Camera trappings, indirect evidence - checklist | |  |
| *Dasypus novemcinctus* | Nine-banded Armadillo | Sao Joao do Piaui | Piaui | -42.09122200000 | -8.26691700000 | Dellcielos et al 2016 | Camera trappings, indirect evidence - checklist | |  |
| *Euphractus sexcinctus* | Yellow Armadillo | Sao Joao do Piaui | Piaui | -42.09122200000 | -8.26691700000 | Dellcielos et al 2016 | Camera trappings, indirect evidence - checklist | |  |
| *Galea spixii* | Spix's Yellow-toothed Cavy | Sao Joao do Piaui | Piaui | -42.09122200000 | -8.26691700000 | Dellcielos et al 2016 | Camera trappings, indirect evidence - checklist | |  |
| *Herpailurus yagouaroundi* | Jaguarundi | Sao Joao do Piaui | Piaui | -42.09122200000 | -8.26691700000 | Dellcielos et al 2016 | Camera trappings, indirect evidence - checklist | |  |
| *Kerodon rupestris* | Rock Cavy | Sao Joao do Piaui | Piaui | -42.09122200000 | -8.26691700000 | Dellcielos et al 2016 | Camera trappings, indirect evidence - checklist | |  |
| *Leopardus emiliae* | Tiger cat | Sao Joao do Piaui | Piaui | -42.09122200000 | -8.26691700000 | Dellcielos et al 2016 | Camera trappings, indirect evidence - checklist | |  |
| *Subulo gouazoubira* | Gray Brocket | Sao Joao do Piaui | Piaui | -42.09122200000 | -8.26691700000 | Dellcielos et al 2016 | Camera trappings, indirect evidence - checklist | |  |
| *Procyon cancrivorus* | Crab eating Raccoon | Sao Joao do Piaui | Piaui | -42.09122200000 | -8.26691700000 | Dellcielos et al 2016 | Camera trappings, indirect evidence - checklist | |  |
| *Sylvilagus brasiliensis* | Tapeti | Sao Joao do Piaui | Piaui | -42.09122200000 | -8.26691700000 | Dellcielos et al 2016 | Camera trappings, indirect evidence - checklist | |  |
| *Tamandua tetradactyla* | Southern Tamandua | Sao Joao do Piaui | Piaui | -42.09122200000 | -8.26691700000 | Dellcielos et al 2016 | Camera trappings, indirect evidence - checklist | |  |
| *Conepatus semistriatus* | Striped hog nosed Skunk | Sao Mamede | Paraiba | -37.15277800000 | -6.93777800000 | Feijo et al 2013 | Museum collection | |  |
| *Euphractus sexcinctus* | Yellow Armadillo | Sao Mamede | Paraiba | -37.15277800000 | -6.93777800000 | Feijo et al 2013 | Museum collection | |  |
| *Herpailurus yagouaroundi* | Jaguarundi | Sao Mamede | Paraiba | -37.15277800000 | -6.93777800000 | Feijo et al 2013 | Museum collection | |  |
| *Kerodon rupestris* | Rock Cavy | Sao Mamede | Paraiba | -37.15277800000 | -6.93777800000 | Gbif | Observation | |  |
| *Leopardus emiliae* | Tiger cat | Sao Mamede | Paraiba | -37.15277800000 | -6.93777800000 | Feijo et al 2013 | Museum collection | |  |
| *Sapajus libidinosus* | Bearded Capuchin | Sao Mamede | Paraiba | -37.15277800000 | -6.93777800000 | Gbif | Observation | |  |
| *Callithrix jacchus* | Common marmoset | Serra da Guia | Sergipe | -37.86531500000 | -9.98117200000 | Rocha et al 2015 | Trails - checklist | |  |
| *Cerdocyon thous* | Crab eating fox | Serra da Guia | Sergipe | -37.86531500000 | -9.98117200000 | Rocha et al 2015 | Trails - checklist | |  |
| *Dasypus novemcinctus* | Nine-banded Armadillo | Serra da Guia | Sergipe | -37.86531500000 | -9.98117200000 | Rocha et al 2015 | Trails - checklist | |  |
| *Didelphis albiventris* | White-eared Opossum | Serra da Guia | Sergipe | -37.86531500000 | -9.98117200000 | Rocha et al 2015 | Trails, capture - checklist | |  |
| *Euphractus sexcinctus* | Yellow Armadillo | Serra da Guia | Sergipe | -37.86531500000 | -9.98117200000 | Rocha et al 2015 | Trails - checklist | |  |
| *Galea spixii* | Spix's Yellow-toothed Cavy | Serra da Guia | Sergipe | -37.86531500000 | -9.98117200000 | Rocha et al 2015 | Capture - checklist | |  |
| *Kerodon rupestris* | Rock Cavy | Serra da Guia | Sergipe | -37.86531500000 | -9.98117200000 | Rocha et al 2015 | Capture - checklist | |  |
| *Leopardus emiliae* | Tiger cat | Serra da Guia | Sergipe | -37.86531500000 | -9.98117200000 | Rocha et al 2015 | Trails - checklist | |  |
| *Subulo gouazoubira* | Gray Brocket | Serra da Guia | Sergipe | -37.86531500000 | -9.98117200000 | Rocha et al 2015 | Trails - checklist | |  |
| *Puma concolor* | Puma | Serra da Guia | Sergipe | -37.86531500000 | -9.98117200000 | Rocha et al 2015 | Trails - checklist | |  |
| *Sylvilagus brasiliensis* | Tapeti | Serra da Guia | Sergipe | -37.86531500000 | -9.98117200000 | Rocha et al 2015 | Trails - checklist | |  |
| *Cerdocyon thous* | Crab eating fox | Serra das Queimadas | Rio Grande do Norte | -36.31486100000 | -5.71116900000 | Marinho et al 2018 | Camera trapping - checklist | |  |
| *Conepatus semistriatus* | Striped hog nosed Skunk | Serra das Queimadas | Rio Grande do Norte | -36.31486100000 | -5.71116900000 | Marinho et al 2018 | Camera trapping - checklist | |  |
| *Dasypus novemcinctus* | Nine-banded Armadillo | Serra das Queimadas | Rio Grande do Norte | -36.31486100000 | -5.71116900000 | Marinho et al 2018 | Camera trapping - checklist | |  |
| *Didelphis albiventris* | White-eared Opossum | Serra das Queimadas | Rio Grande do Norte | -36.31486100000 | -5.71116900000 | Marinho et al 2018 | Camera trapping - checklist | |  |
| *Euphractus sexcinctus* | Yellow Armadillo | Serra das Queimadas | Rio Grande do Norte | -36.31486100000 | -5.71116900000 | Marinho et al 2018 | Camera trapping - checklist | |  |
| *Galictis cuja* | Lesser Grison | Serra das Queimadas | Rio Grande do Norte | -36.31486100000 | -5.71116900000 | Marinho et al 2018 | Camera trapping - checklist | |  |
| *Leopardus emiliae* | Tiger cat | Serra das Queimadas | Rio Grande do Norte | -36.31486100000 | -5.71116900000 | Marinho et al 2018 | Camera trapping - checklist | |  |
| *Subulo gouazoubira* | Gray Brocket | Serra das Queimadas | Rio Grande do Norte | -36.31486100000 | -5.71116900000 | Marinho et al 2018 | Camera trapping - checklist | |  |
| *Procyon cancrivorus* | Crab eating Raccoon | Serra das Queimadas | Rio Grande do Norte | -36.31486100000 | -5.71116900000 | Marinho et al 2018 | Camera trapping - checklist | |  |
| *Tamandua tetradactyla* | Southern Tamandua | Serra das Queimadas | Rio Grande do Norte | -36.31486100000 | -5.71116900000 | Marinho et al 2018 | Camera trapping - checklist | |  |
| *Callithrix jacchus* | Common marmoset | Serra de Baturite | Ceara | -39.01243100000 | -4.36612100000 | Fernandes-Ferreira et al 2015 | Collection, interview, picture - hunter | |  |
| *Cerdocyon thous* | Crab eating fox | Serra de Baturite | Ceara | -39.01243100000 | -4.36612100000 | Fernandes-Ferreira et al 2015 | Collection, observation, picture | |  |
| *Coendou baturitensis* | Brazilian Porcupine | Serra de Baturite | Ceara | -39.01243100000 | -4.36612100000 | Feijo et al 2013, Fernandes-Ferreira et al 2015 | Collection, interview, picture - hunter | |  |
| *Conepatus semistriatus* | Striped hog nosed Skunk | Serra de Baturite | Ceara | -39.01243100000 | -4.36612100000 | Fernandes-Ferreira et al 2015 | Observation, interview - hunter | |  |
| *Cuniculus paca* | Agouti | Serra de Baturite | Ceara | -39.01243100000 | -4.36612100000 | Fernandes-Ferreira et al 2015 | Observation, interview - hunter | |  |
| *Dasypus novemcinctus* | Nine-banded Armadillo | Serra de Baturite | Ceara | -39.01243100000 | -4.36612100000 | Fernandes-Ferreira et al 2015 | Collection, interview - hunter | |  |
| *Didelphis albiventris* | White-eared Opossum | Serra de Baturite | Ceara | -39.01243100000 | -4.36612100000 | Fernandes-Ferreira et al 2015 | Collection, interview, picture - hunter | |  |
| *Euphractus sexcinctus* | Yellow Armadillo | Serra de Baturite | Ceara | -39.01243100000 | -4.36612100000 | Fernandes-Ferreira et al 2015 | Collection, interview, picture - hunter | |  |
| *Galea spixii* | Spix's Yellow-toothed Cavy | Serra de Baturite | Ceara | -39.01243100000 | -4.36612100000 | Fernandes-Ferreira et al 2015 | Collection, interview - hunter | |  |
| *Galictis cuja* | Lesser Grison | Serra de Baturite | Ceara | -39.01243100000 | -4.36612100000 | Fernandes-Ferreira et al 2015 | Observation | |  |
| *Herpailurus yagouaroundi* | Jaguarundi | Serra de Baturite | Ceara | -39.01243100000 | -4.36612100000 | Feijo et al 2013, Fernandes-Ferreira et al 2015 | Collection, interview - hunter | |  |
| *Kerodon rupestris* | Rock Cavy | Serra de Baturite | Ceara | -39.01243100000 | -4.36612100000 | Fernandes-Ferreira et al 2015 | Observation, interview - hunter | |  |
| *Leopardus emiliae* | Tiger cat | Serra de Baturite | Ceara | -39.01243100000 | -4.36612100000 | Feijo et al 2013, Fernandes-Ferreira et al 2015 | Collection, interview - hunter | |  |
| *Leopardus pardalis* | Ocelot | Serra de Baturite | Ceara | -39.01243100000 | -4.36612100000 | Feijo et al 2013, Fernandes-Ferreira et al 2015 | Collection, interview - hunter | |  |
| *Leopardus wiedii* | Margay | Serra de Baturite | Ceara | -39.01243100000 | -4.36612100000 | Feijo et al 2013, Fernandes-Ferreira et al 2015 | Collection, interview - hunter | |  |
| *Subulo gouazoubira* | Gray Brocket | Serra de Baturite | Ceara | -39.01243100000 | -4.36612100000 | Feijo et al 2013, Fernandes-Ferreira et al 2015 | Collection, interview - hunter | |  |
| *Panthera onca* | Jaguar | Serra de Baturite | Ceara | -39.01243100000 | -4.36612100000 | Fernandes-Ferreira et al 2015 | Observation, interview - hunter | |  |
| *Procyon cancrivorus* | Crab eating Raccoon | Serra de Baturite | Ceara | -39.01243100000 | -4.36612100000 | Feijo et al 2013, Fernandes-Ferreira et al 2015 | Collection, interview, picture - hunter | |  |
| *Puma concolor* | Puma | Serra de Baturite | Ceara | -39.01243100000 | -4.36612100000 | Fernandes-Ferreira et al 2015 | Collection, interview - hunter | |  |
| *Speothos venaticus* | Bush Dog | Serra de Baturite | Ceara | -39.01243100000 | -4.36612100000 | Feijo et al 2013, Fernandes-Ferreira et al 2015 | Collection, interview - hunter | |  |
| *Tamandua tetradactyla* | Southern Tamandua | Serra de Baturite | Ceara | -39.01243100000 | -4.36612100000 | Feijo et al 2013, Fernandes-Ferreira et al 2015 | Collection, interview - hunter | |  |
| *Callithrix jacchus* | Common marmoset | Serra de Santa Catarina | Paraiba | -38.18666700000 | -7.01277800000 | Campos et al 2018, Lucena et al 2018 - Livro | Interview, observation, camera trapping - checklist | |  |
| *Conepatus semistriatus* | Striped hog nosed Skunk | Serra de Santa Catarina | Paraiba | -38.18666700000 | -7.01277800000 | Campos et al 2018, Lucena et al 2018 - Livro | Interview, observation, camera trapping, footprint - checklist | |  |
| *Dasypus novemcinctus* | Nine-banded Armadillo | Serra de Santa Catarina | Paraiba | -38.18666700000 | -7.01277800000 | Lucena et al 2018 - Livro | Interview -checklist | |  |
| *Didelphis albiventris* | White-eared Opossum | Serra de Santa Catarina | Paraiba | -38.18666700000 | -7.01277800000 | Campos et al 2018 - Livro | Interview, observation, camera trapping - checklist | |  |
| *Euphractus sexcinctus* | Yellow Armadillo | Serra de Santa Catarina | Paraiba | -38.18666700000 | -7.01277800000 | Campos et al 2018, Lucena et al 2018 - Livro | Interview, camera trapping - checklist | |  |
| *Galea spixii* | Spix's Yellow-toothed Cavy | Serra de Santa Catarina | Paraiba | -38.18666700000 | -7.01277800000 | Campos et al 2018, Lucena et al 2018 - Livro | Interview, camera trapping, footprint - checklist | |  |
| *Herpailurus yagouaroundi* | Jaguarundi | Serra de Santa Catarina | Paraiba | -38.18666700000 | -7.01277800000 | Lucena et al 2018 - Livro | Interview - checklist | |  |
| *Kerodon rupestris* | Rock Cavy | Serra de Santa Catarina | Paraiba | -38.18666700000 | -7.01277800000 | Campos et al 2018, Lucena et al 2018 - Livro | Interview, footprint - checklist | |  |
| *Leopardus pardalis* | Ocelot | Serra de Santa Catarina | Paraiba | -38.18666700000 | -7.01277800000 | Campos et al 2018, Lucena et al 2018 - Livro | Interview, camera trapping checklist | |  |
| *Subulo gouazoubira* | Gray Brocket | Serra de Santa Catarina | Paraiba | -38.18666700000 | -7.01277800000 | Campos et al 2018, Lucena et al 2018 - Livro | Interview, observation - checklist | |  |
| *Dicotyles tajacu* | Collaredy Pecary | Serra de Santa Catarina | Paraiba | -38.18666700000 | -7.01277800000 | Lucena et al 2018 - Livro | Interview -checklist | |  |
| *Puma concolor* | Puma | Serra de Santa Catarina | Paraiba | -38.18666700000 | -7.01277800000 | Campos et al 2018, Lucena et al 2018 - Livro | Interview, footprint, camera trapping, observation checklist | |  |
| *Sapajus libidinosus* | Bearded Capuchin | Serra de Santa Catarina | Paraiba | -38.18666700000 | -7.01277800000 | Campos et al 2018, Lucena et al 2018 - Livro | Interview, footprint, camera trapping - checklist | |  |
| *Tamandua tetradactyla* | Southern Tamandua | Serra de Santa Catarina | Paraiba | -38.18666700000 | -7.01277800000 | Campos et al 2018, Lucena et al 2018 - Livro | Interview, camera trapping - checklist | |  |
| *Cerdocyon thous* | Crab eating fox | Serra de Santana | Bahia | -40.19583300000 | -10.36472200000 | Pererira & Peixoto 2017 | Footprint - checklist | |  |
| *Dasyprocta leporina* | Red humped agouti | Serra de Santana | Bahia | -40.19583300000 | -10.36472200000 | Pererira & Peixoto 2017 | Indirect evidence, footprint - checklist | |  |
| *Dasypus novemcinctus* | Nine-banded Armadillo | Serra de Santana | Bahia | -40.19583300000 | -10.36472200000 | Pererira & Peixoto 2017 | Footprint - checklist | |  |
| *Euphractus sexcinctus* | Yellow Armadillo | Serra de Santana | Bahia | -40.19583300000 | -10.36472200000 | Pererira & Peixoto 2017 | Indirect evidence - checklist | |  |
| *Galea spixii* | Spix's Yellow-toothed Cavy | Serra de Santana | Bahia | -40.19583300000 | -10.36472200000 | Pererira & Peixoto 2017 | Indirect evidence - checklist | |  |
| *Leopardus pardalis* | Ocelot | Serra de Santana | Bahia | -40.19583300000 | -10.36472200000 | Pererira & Peixoto 2017 | Footprint - checklist | |  |
| *Lycalopex vetulus* | Hoary fox | Serra de Santana | Bahia | -40.19583300000 | -10.36472200000 | Pererira & Peixoto 2017 | Indirect evidence, footprint - checklist | |  |
| *Subulo gouazoubira* | Gray Brocket | Serra de Santana | Bahia | -40.19583300000 | -10.36472200000 | Pererira & Peixoto 2017 | Indirect evidence, observation, footprint - checklist | |  |
| *Dicotyles tajacu* | Collaredy Pecary | Serra de Santana | Bahia | -40.19583300000 | -10.36472200000 | Pererira & Peixoto 2017 | Indirect evidence, footprint - checklist | |  |
| *Puma concolor* | Puma | Serra de Santana | Bahia | -40.19583300000 | -10.36472200000 | Pererira & Peixoto 2017 | Observation, footprint- checklist | |  |
| *Sylvilagus brasiliensis* | Tapeti | Serra de Santana | Bahia | -40.19583300000 | -10.36472200000 | Pererira & Peixoto 2017 | Indirect evidence - checklist | |  |
| *Tamandua tetradactyla* | Southern Tamandua | Serra de Santana | Bahia | -40.19583300000 | -10.36472200000 | Pererira & Peixoto 2017 | Indirect evidence, footprint - checklist | |  |
| *Cerdocyon thous* | Crab eating fox | Serra dos Macacos | Sergipe | -37.98647200000 | -10.88152800000 | Dias et al 2004 | Indirect evidence, interview - checklist carnivoro | |  |
| *Conepatus semistriatus* | Striped hog nosed Skunk | Serra dos Macacos | Sergipe | -37.98647200000 | -10.88152800000 | Dias et al 2004 | Indirect evidence, interview - checklist carnivoro | |  |
| *Eira barbara* | Tayra | Serra dos Macacos | Sergipe | -37.98647200000 | -10.88152800000 | Dias et al 2004 | Indirect evidence, interview - checklist carnivoro | |  |
| *Galictis cuja* | Lesser Grison | Serra dos Macacos | Sergipe | -37.98647200000 | -10.88152800000 | Dias et al 2004 | Indirect evidence, interview - checklist carnivoro | |  |
| *Herpailurus yagouaroundi* | Jaguarundi | Serra dos Macacos | Sergipe | -37.98647200000 | -10.88152800000 | Dias et al 2004 | Indirect evidence, interview - checklist carnivoro | |  |
| *Leopardus pardalis* | Ocelot | Serra dos Macacos | Sergipe | -37.98647200000 | -10.88152800000 | Dias et al 2004 | Indirect evidence, interview - checklist carnivoro | |  |
| *Procyon cancrivorus* | Crab eating Raccoon | Serra dos Macacos | Sergipe | -37.98647200000 | -10.88152800000 | Dias et al 2004 | Indirect evidence, interview - checklist carnivoro | |  |
| *Dasyprocta prymnolopha* | red-orange rump agoutis | Serra dos Montes Altos | Bahia | -42.96804100000 | -14.45726500000 | Barros & Almeida 2018 | Camera trapping - checklist | |  |
| *Eira barbara* | Tayra | Serra dos Montes Altos | Bahia | -42.96804100000 | -14.45726500000 | Barros & Almeida 2018 | Camera trapping - checklist | |  |
| *Galea spixii* | Spix's Yellow-toothed Cavy | Serra dos Montes Altos | Bahia | -42.96804100000 | -14.45726500000 | Barros & Almeida 2018 | Camera trapping - checklist | |  |
| *Kerodon rupestris* | Rock Cavy | Serra dos Montes Altos | Bahia | -42.96804100000 | -14.45726500000 | Barros & Almeida 2018 | Camera trapping - checklist | |  |
| *Subulo gouazoubira* | Gray Brocket | Serra dos Montes Altos | Bahia | -42.96804100000 | -14.45726500000 | Barros & Almeida 2018 | Camera trapping - checklist | |  |
| *Dicotyles tajacu* | Collaredy Pecary | Serra dos Montes Altos | Bahia | -42.96804100000 | -14.45726500000 | Barros & Almeida 2018 | Camera trapping - checklist | |  |
| *Sapajus xanthosternos* | Buff headed Capuchin | Serra dos Montes Altos | Bahia | -42.96804100000 | -14.45726500000 | Barros & Almeida 2018 | Camera trapping - checklist | |  |
| *Tamandua tetradactyla* | Southern Tamandua | Serra dos Montes Altos | Bahia | -42.96804100000 | -14.45726500000 | Barros & Almeida 2018 | Camera trapping - checklist | |  |
| *Tolypeutes tricinctus* | Brazilian Three banded Armadillo | Serra dos Montes Altos | Bahia | -42.96804100000 | -14.45726500000 | Barros & Almeida 2018 | Camera trapping - checklist | |  |
| *Cerdocyon thous* | Crab eating fox | Serrinha dos Pintos | Rio Grande do Norte | -37.99633100000 | -6.09030000000 | Marinho et al 2018 | Camera trapping - checklist | |  |
| *Dasypus novemcinctus* | Nine-banded Armadillo | Serrinha dos Pintos | Rio Grande do Norte | -37.99633100000 | -6.09030000000 | Marinho et al 2018 | Camera trapping - checklist | |  |
| *Didelphis albiventris* | White-eared Opossum | Serrinha dos Pintos | Rio Grande do Norte | -37.99633100000 | -6.09030000000 | Marinho et al 2018 | Camera trapping - checklist | |  |
| *Euphractus sexcinctus* | Yellow Armadillo | Serrinha dos Pintos | Rio Grande do Norte | -37.99633100000 | -6.09030000000 | Marinho et al 2018 | Camera trapping - checklist | |  |
| *Herpailurus yagouaroundi* | Jaguarundi | Serrinha dos Pintos | Rio Grande do Norte | -37.99633100000 | -6.09030000000 | Marinho et al 2018 | Camera trapping - checklist | |  |
| *Leopardus emiliae* | Tiger cat | Serrinha dos Pintos | Rio Grande do Norte | -37.99633100000 | -6.09030000000 | Marinho et al 2018 | Camera trapping - checklist | |  |
| *Subulo gouazoubira* | Gray Brocket | Serrinha dos Pintos | Rio Grande do Norte | -37.99633100000 | -6.09030000000 | Marinho et al 2018 | Camera trapping - checklist | |  |
| *Procyon cancrivorus* | Crab eating Raccoon | Serrinha dos Pintos | Rio Grande do Norte | -37.99633100000 | -6.09030000000 | Marinho et al 2018 | Camera trapping - checklist | |  |
| *Tamandua tetradactyla* | Southern Tamandua | Serrinha dos Pintos | Rio Grande do Norte | -37.99633100000 | -6.09030000000 | Marinho et al 2018 | Camera trapping - checklist | |  |
| *Callithrix jacchus* | Common marmoset | Solanea | Paraiba | -35.54000000000 | -6.75500000000 | Silva Santos et al 2019 | Interview - hunter | |  |
| *Cerdocyon thous* | Crab eating fox | Solanea | Paraiba | -35.54000000000 | -6.75500000000 | Silva Santos et al 2019 | Interview - hunter | |  |
| *Conepatus semistriatus* | Striped hog nosed Skunk | Solanea | Paraiba | -35.54000000000 | -6.75500000000 | Silva Santos et al 2019 | Interview - hunter | |  |
| *Euphractus sexcinctus* | Yellow Armadillo | Solanea | Paraiba | -35.54000000000 | -6.75500000000 | Silva Santos et al 2019 | Interview - hunter | |  |
| *Galea spixii* | Spix's Yellow-toothed Cavy | Solanea | Paraiba | -35.54000000000 | -6.75500000000 | Silva Santos et al 2019 | Interview - hunter | |  |
| *Herpailurus yagouaroundi* | Jaguarundi | Solanea | Paraiba | -35.54000000000 | -6.75500000000 | Silva Santos et al 2019 | Interview - hunter | |  |
| *Kerodon rupestris* | Rock Cavy | Solanea | Paraiba | -35.54000000000 | -6.75500000000 | Silva Santos et al 2019 | Interview - hunter | |  |
| *Sylvilagus brasiliensis* | Tapeti | Solanea | Paraiba | -35.54000000000 | -6.75500000000 | Silva Santos et al 2019 | Interview - hunter | |  |
| *Tamandua tetradactyla* | Southern Tamandua | Solanea | Paraiba | -35.54000000000 | -6.75500000000 | Silva Santos et al 2019 | Interview - hunter | |  |
| *Callithrix jacchus* | Common marmoset | Sume | Paraiba | -36.91583900000 | -7.49924700000 | Feijo et al 2013 | Interview | |  |
| *Cavia aperea* | Brazilian guinea pig | Sume | Paraiba | -36.91583900000 | -7.49924700000 | Barbosa et al 2016 | Interview - hunter | |  |
| *Cerdocyon thous* | Crab eating fox | Sume | Paraiba | -36.91583900000 | -7.49924700000 | Silva Policarpo et al 2019, Barbosa et al 2016 | Interview - medice use, hunter | |  |
| *Conepatus semistriatus* | Striped hog nosed Skunk | Sume | Paraiba | -36.91583900000 | -7.49924700000 | Silva Policarpo et al 2019, Barbosa et al 2016 | Interview - medice use, hunter | |  |
| *Dasypus novemcinctus* | Nine-banded Armadillo | Sume | Paraiba | -36.91583900000 | -7.49924700000 | Silva Policarpo et al 2019, Barbosa et al 2016 | Interview - medice use, hunter | |  |
| *Didelphis marsupialis* | Common Opossum | Sume | Paraiba | -36.91583900000 | -7.49924700000 | Barbosa et al 2016 | Interview - hunter | |  |
| *Euphractus sexcinctus* | Yellow Armadillo | Sume | Paraiba | -36.91583900000 | -7.49924700000 | Silva Policarpo et al 2019, Barbosa et al 2016, Feijo et al 2013 | Interview - medice use, hunter, collection | |  |
| *Galea spixii* | Spix's Yellow-toothed Cavy | Sume | Paraiba | -36.91583900000 | -7.49924700000 | Silva Policarpo et al 2019, Barbosa et al 2016 | Interview - medice use, hunter | |  |
| *Galictis vittata* | Greater Grison | Sume | Paraiba | -36.91583900000 | -7.49924700000 | Barbosa et al 2016 | Interview - hunter | |  |
| *Herpailurus yagouaroundi* | Jaguarundi | Sume | Paraiba | -36.91583900000 | -7.49924700000 | Silva Policarpo et al 2019, Barbosa et al 2016 | Interview - medice use, hunter | |  |
| *Kerodon rupestris* | Rock Cavy | Sume | Paraiba | -36.91583900000 | -7.49924700000 | Silva Policarpo et al 2019, Barbosa et al 2016 | Interview - medice use, hunter | |  |
| *Leopardus emiliae* | Tiger cat | Sume | Paraiba | -36.91583900000 | -7.49924700000 | Silva Policarpo et al 2019, Barbosa et al 2016 | Interview - medice use, hunter | |  |
| *Leopardus pardalis* | Ocelot | Sume | Paraiba | -36.91583900000 | -7.49924700000 | Barbosa et al 2016 | Interview - hunter | |  |
| *Leopardus wiedii* | Margay | Sume | Paraiba | -36.91583900000 | -7.49924700000 | Silva Policarpo et al 2019, Barbosa et al 2016 | Interview - medice use, hunter | |  |
| *Procyon cancrivorus* | Crab eating Raccoon | Sume | Paraiba | -36.91583900000 | -7.49924700000 | Silva Policarpo et al 2019, Barbosa et al 2016 | Interview - medice use, hunter | |  |
| *Tamandua tetradactyla* | Southern Tamandua | Sume | Paraiba | -36.91583900000 | -7.49924700000 | Silva Policarpo et al 2019, Barbosa et al 2016 | Interview - medice use, hunter | |  |
| *Callithrix jacchus* | Common marmoset | Teixeira | Paraiba | -37.30737000000 | -7.19339700000 | Feijo et al 2013 | Museum collection | |  |
| *Cerdocyon thous* | Crab eating fox | Teixeira | Paraiba | -37.30737000000 | -7.19339700000 | Feijo et al 2013 | Interview | |  |
| *Herpailurus yagouaroundi* | Jaguarundi | Teixeira | Paraiba | -37.30737000000 | -7.19339700000 | Feijo et al 2013 | Museum collection | |  |
| *Leopardus emiliae* | Tiger cat | Teixeira | Paraiba | -37.30737000000 | -7.19339700000 | Feijo et al 2013 | Museum collection | |  |
| *Puma concolor* | Puma | Teixeira | Paraiba | -37.30737000000 | -7.19339700000 | Feijo et al 2013 | Museum collection | |  |
| *Sapajus libidinosus* | Bearded Capuchin | Teixeira | Paraiba | -37.30737000000 | -7.19339700000 | Feijo et al 2013 | Museum collection | |  |
| *Callithrix jacchus* | Common marmoset | Umbuzeiro do Matuto | Sergipe | -37.52301700000 | -9.84988300000 | Bispo 1998 |  | |  |
| *Cerdocyon thous* | Crab eating fox | Umbuzeiro do Matuto | Sergipe | -37.52301700000 | -9.84988300000 | Bispo 1998 |  | |  |
| *Dasyprocta prymnolopha* | red-orange rump agoutis | Umbuzeiro do Matuto | Sergipe | -37.52301700000 | -9.84988300000 | Bispo 1998 |  | |  |
| *Dasypus novemcinctus* | Nine-banded Armadillo | Umbuzeiro do Matuto | Sergipe | -37.52301700000 | -9.84988300000 | Bispo 1998 |  | |  |
| *Dasypus septemcinctus* | Brazilian Lesser long-nosed Armadillo | Umbuzeiro do Matuto | Sergipe | -37.52301700000 | -9.84988300000 | Bispo 1998 |  | |  |
| *Galea spixii* | Spix's Yellow-toothed Cavy | Umbuzeiro do Matuto | Sergipe | -37.52301700000 | -9.84988300000 | Bispo 1998 |  | |  |
| *Kerodon rupestris* | Rock Cavy | Umbuzeiro do Matuto | Sergipe | -37.52301700000 | -9.84988300000 | Bispo 1998 |  | |  |
| *Subulo gouazoubira* | Gray Brocket | Umbuzeiro do Matuto | Sergipe | -37.52301700000 | -9.84988300000 | Bispo 1998 |  | |  |
| *Panthera onca* | Jaguar | Umbuzeiro do Matuto | Sergipe | -37.52301700000 | -9.84988300000 | Bispo 1998 |  | |  |
| *Dicotyles tajacu* | Collaredy Pecary | Umbuzeiro do Matuto | Sergipe | -37.52301700000 | -9.84988300000 | Bispo 1998 |  | |  |
| *Procyon cancrivorus* | Crab eating Raccoon | Umbuzeiro do Matuto | Sergipe | -37.52301700000 | -9.84988300000 | Bispo 1998 |  | |  |
| *Puma concolor* | Puma | Umbuzeiro do Matuto | Sergipe | -37.52301700000 | -9.84988300000 | Bispo 1998 |  | |  |
| *Sylvilagus brasiliensis* | Tapeti | Umbuzeiro do Matuto | Sergipe | -37.52301700000 | -9.84988300000 | Bispo 1998 |  | |  |
| *Tamandua tetradactyla* | Southern Tamandua | Umbuzeiro do Matuto | Sergipe | -37.52301700000 | -9.84988300000 | Bispo 1998 |  | |  |
| *Cabassous tatouay* | Greater Naked-tailed Armadillo | Varza grande | Ceara | -39.25675500000 | -6.81610500000 | Feijo et al 2013 | Interview | |  |
| *Callithrix jacchus* | Common marmoset | Varza grande | Ceara | -39.25675500000 | -6.81610500000 | Feijo et al 2013 | Interview | |  |
| *Cerdocyon thous* | Crab eating fox | Varza grande | Ceara | -39.25675500000 | -6.81610500000 | Feijo et al 2013 | Interview | |  |
| *Conepatus semistriatus* | Striped hog nosed Skunk | Varza grande | Ceara | -39.25675500000 | -6.81610500000 | Feijo et al 2013 | Interview | |  |
| *Galictis cuja* | Lesser Grison | Varza grande | Ceara | -39.25675500000 | -6.81610500000 | Feijo et al 2013 | Interview | |  |
| *Leopardus emiliae* | Tiger cat | Varza grande | Ceara | -39.25675500000 | -6.81610500000 | Feijo et al 2013 | Interview | |  |
| *Subulo gouazoubira* | Gray Brocket | Varza grande | Ceara | -39.25675500000 | -6.81610500000 | Feijo et al 2013 | Interview | |  |
| *Sapajus libidinosus* | Bearded Capuchin | Varza grande | Ceara | -39.25675500000 | -6.81610500000 | Feijo et al 2013 | Interview | |  |
