# Supplementary material for: Reconstructing the degree of mammal defaunation throughout the Caatinga - the largest dry tropical forest region of South America
Source: PLoS One. 2025 Nov 24;20(11):e0336562. doi: 10.1371/journal.pone.0336562 (PMC12643294; doi:10.1371/journal.pone.0336562)
Supplement: S2 Table — (DOCX) [file pone.0336562.s003.docx]

**S2 table**. Protocol about species distribution model (SDM) and following the Zurell, et al 2020.

([**dx.doi.org/10.17504/protocols.io.81wgbwqrogpk/v1**](https://dx.doi.org/10.17504/protocols.io.81wgbwqrogpk/v1))

| **ODMAP element** | C**ontents** |
| --- | --- |
| **OVERVIEW** |  |
| ***Authorship*** | • **Authors**: Nathália Fernandes Canassa, Carlos A. Peres, Célia Cristina Clemente Machado,  Helder Farias P. Araujo. |
|  | • **Contact** email: [nfcanassabio@gmail.com](mailto:nfcanassabio@gmail.com) |
|  | • **Title:** Reconstructing the degree of mammal defaunation throughout the Caatinga the largest Dry Tropical Forest region of South America |
|  | • **DOI:** |
| ***Model objective*** | • **Objective**: Mapping/interpolation. |
|  | • **Target outputs**: Maps of relative probability of presence, representing environmental suitability for the modeled species based on climatic and altitude variables, without considering human impacts. |
| ***Taxon*** | Species medium- to large-bodied mammals in Caatinga |
| ***Location*** | Caatinga Ecossystem, Brazil |
| ***Scale of analysis*** | • **Spatial extent (Lon/Lat):** Longitude -81.2083° W to -56.7500° W,  Latitude -32.5000° S to 16.2917° N. |
|  | • **Spatial resolution**: 2.5 arcminutes (≈ 4,630 m) |
|  | • **Temporal extent/time period**: Current (WorldClim version 2.1,  high-resolution interpolated climate data agregated across a target temporal  range of 1970-2000) |
|  | • **Temporal resolution: Multi-decadal average** – The dataset provides long-term averaged  climate variables rather than yearly or monthly data, meaning it represents mean climatic  conditions over the 1970–2000 period rather than capturing interannual variability |
|  | • **Type of extent boundary**: Rectangular |
| ***Biodiversity data overview*** | • **Observation type**: Bibliographic survey - based papers about in camera traps, interview, footprint, and Global Biodiversity Information Facility (GBIF – https://www.gbif.org) and Specieslink (https://specieslink.net) platforms to supplement spatially explicit data on species occurrences |
|  | • **Response/Data type:** Presence-only |
| ***Type of predictors*** | Climatic, topographic. |
| ***Conceptual model / Hypotheses*** | • **Hypotheses about species-environment relationships:** The potential distribution of the studied mammals reflects the optimal environmental conditions for their occurrence, excluding human interference. |
| ***Assumptions*** | **State critical model assumptions:** |
|  | 1.The model assumes that species distributions reflect environmental suitability in the absence of human impact. |
|  | 2. Species fill their niche and do not occur elsewhere. |
|  | 3. Species occurrence data is adequate and representative. |
|  | 4. Independence of species observations. |
|  | 5. Relevant ecological drivers (or proxies) of species distributions are included. |
|  | 6. Predictors are free of error: |
| ***SDM algorithm*** | • **Algorithms**: MaxEnt (Maximum Entropy Model) |
|  | • **Model complexity**: MaxEnt enables robust predictions with limited occurrence data by employing a presence-only approach. We used multiple replicates (bootstrap with 10 repetitions) to improve reliability, while selecting uncorrelated variables helped prevent collinearity. Additionally, MaxEnt parameters (e.g., random test points, random seed) were chosen to enhance model generalization. |
|  | • **Ensembles**: None |
| ***Model workflow*** | **• Conceptual description of modelling step**s: Model fitting was performed using multiple bootstrap replicates (10 repetitions), which enhances the model's reliability and reduces the impact of random variability in the data. Model performance was evaluated using the area under the curve (AUC) of the receiver operating characteristic (ROC), which ensures that the model is making accurate and reliable predictions. The correlation between environmental variables was tested, and highly correlated variables were removed to  reduce collinearity, which could compromise model accuracy. To delimit the species' occurrence areas, a MaxEnt threshold was applied, which defines a cutoff value on the habitat suitability index. This threshold is used to convert the continuous predictions into a binary representation, where areas with values above the threshold are considered suitable for the species, while those below the cutoff are considered unsuitable.The threshold selection was based on model performance analysis (e.g., using ROC curves or maximizing accuracy), ensuring that the chosen cutoff realistically reflected the species' potential occurrence areas. This helps to identify regions where the species is most likely to occur, based on the predicted environmental conditions. |
| ***Software, codes and data*** | **• Software:** Analyses were conducted in R version 3.5.3. The MaxEnt version 3.4.3 using packages "dismo" version 1.3-5  **• Data availability:** Data are available in an open, online, digital  repository (DOI: 10.17632/bdmzzytkdm.1) |
| **DATA** | |
| ***Biodiversity data*** | **• Taxon names:** All species are listed in the Supplementary Information (Table S1)  **• Ecological level:** Species level  **•Biodiversity data source:** Data were derived from literature sources, but all data are openly available and platforms to supplement spatially explicit data on species occurrences such as Gbif and Species Link  **•Sampling design:** Web of Science, and ScienceDirect databases, employing word combinations (in English and Portuguese) such as “medium- to large-bodied mammals”, “checklist”, “Caatinga”, “hunting”, and “ethnofauna” to access any available information on MLBM assemblages across the Caatinga. We also used the Global Biodiversity Information Facility (GBIF – <https://www.gbif.org>) and the Specieslink (ttps://specieslink.net) platform to supplement spatially explicit data on species occurrences.  **• Sample size per taxon:** Total 51 species and 8169 geographic coordinates  **• Country/region:** We compiled all the data for South America.  **• Details on scaling**: All occurrence data were selected from across South America and later cropped to match the regional boundaries of the Caatinga domain, using de Software QGIS, Madeira version 4.4.14.  **• Details on data cleaning/filtering steps:** We searched for the names of all 51 species known to occur in the Caatinga region of South America. We compiled all the data into a single table and verified the geographic coordinates for each species using QGIS software (Madeira version 4.4.14). We only used geographic coordinates within the South American limits, excluding any localities outside this boundary, duplicate localities, and locations within 2 km of each other for the same species. |
| ***Data partitioning*** | **• Selection of training data (for model fitting):** We conducted 10 replicate analysis for each species based on a 25% bootstrap of available occurrence data. |
| ***Predictor variables*** | Diurnal amplitude of mean temperature (BIO2), temperature seasonality (BIO4), mean temperature of the wettest (BIO8) and driest (BIO09) quarter, annual precipitation (BIO12), precipitation seasonality (BIO15), precipitation of the driest (BIO17), warmest (BIO18) and coldest (BIO19) quarter, and terrain elevation  **• Details on data sources:** Current WorldClim version 2.1 ([www.worldclim.org](http://www.worldclim.org))  **• Spatial resolution and spatial extent of raw data:** Spatial resolution of 2.5 arcminutes (≈ 4,630 m)  **• Map Spatial Reference: XY Coordenate System -** GCS WGS 1984. **Datum -** D WGS 1984 |
| **MODEL** | |
| ***Variable pre-selection*** | **• Details on pre-selection of variables:** we used 19 climate variables along with elevation data extracted from WorldClim version 2.1( www.worldclim.org), at a spatial resolution of  2.5 arcminutes (≈ 4,630 m). WorldClim provides high-resolution interpolated climate data, derived from 9,000 to 60,000 weather stations worldwide, aggregated across a target temporal range of 1970–2000 |
| ***Multicollinearity*** | · To avoid redundancy, we included in the models all variables that were correlated with each other by less than 0.8, using a Pearson correlation matrix calculated using the R package “vegan” (version 3.5.3) |
| ***Model settings*** | We used Receiver Operator Characteristic (ROC) statistics to assess  model accuracy, with 10 replicates of 10,000 maximum iterations,  10% of the average replicates were randomized as test data,  while the remainder were randomized to train the model during each replicate |
| ***Model estimates*** | • **Assessment of variable importance**: we used the jackknife option to identify variables not contributing importantly to model robustness |
| ***Threshold selection*** | **• Details on threshold selection:** We selected the thresholds for each species that defined the smallest potential habitat following a conservative approach to avoid overestimating species geographic distributions (Table S2) |
| **ASSESSMENT** | |
| ***Performance statistics*** | **• Performance statistics estimated on training data:** performance statistics were estimated using the training data during each bootstrap replicate. For each of the 10 repetitions, MaxEnt evaluated model accuracy by comparing predicted and observed occurrences. Standard metrics such as AUC (Area Under the Curve) were calculated to assess model performance, ensuring robust and reliable predictions. The use of multiple replicates enhances the stability of the performance estimates and minimizes the risk of overfitting.  **• Performance statistics estimated on validation data (from data partitioning):** although no explicit data partitioning was applied,  we used a random test point during each bootstrap replicate.  These random test points served as a form of validation by evaluating the model’s performance on data that was not used during model fitting. Performance statistics, such as AUC, were calculated based on these test points, providing an estimate of model accuracy and its ability to generalize to unseen data. |
| ***Plausibility check*** | **Res • Response plots:** We used partial dependence plots to check the ecological plausibility of fitted relationships in MaxEnt models. |
| **PREDICTION** | |
| ***Prediction output*** | **• Pr • Prediction unit:** Predictions of relative probability of presence  expressed on a continuous scale.  **• Po • Post-processing:** after thresholds selection, clipping was performed to generate binary  maps. |
| ***Uncertainty quantification*** | •**Alg• Algorithmic uncertainty, if applicable**: None  • **Un• Uncertainty in input data, if applicable**: None  • **Ef • Effect of parameter uncertainty, error propagation, if applicable**: None |
