# Supplementary material for: Reconstructing the degree of mammal defaunation throughout the Caatinga - the largest dry tropical forest region of South America
Source: PLoS One. 2025 Nov 24;20(11):e0336562. doi: 10.1371/journal.pone.0336562 (PMC12643294; doi:10.1371/journal.pone.0336562)
Supplement: S3 Table — (DOCX) [file pone.0336562.s004.docx]

**S3 table.** Description and value of thresholds and UAC used to generate the potential species distribution models of mammal species.

| **Species** | **Threshold** | **Values** | **AUC** |  |
| --- | --- | --- | --- | --- |
| *Alouatta belzebul* | Equate entropy of thresholded and original distribuitions Cloglog threshold | 0.1664 | 0.963 |  |
| *Alouatta caraya* | Fixed comulative value 10 Clolog threshold | 0.2691 | 0.929 |  |
| *Bradypus variegatus* | Equal training sensitivity and specificity Clolog threshold | 0.4175 | 0.807 |  |
| *Cabassous tatouay* | Fixed comulative value 10 Clolog threshold | 0.1769 | 0.903 |  |
| *Callicebus barbarabrownae* | Minimun training presence Clolog threshold | 0.1770 | 0.995 |  |
| *Callithrix jacchus* | Maximun test sensitivity plus specificity Cloglog threshold | 0.2183 | 0.950 |  |
| *Callithrix penicillata* | Equal training sensitivity and specificity Clolog threshold | 0.3097 | 0.958 |  |
| *Cavea aperea* | Maximun test sensitivity plus specificity Cloglog threshold | 0.3372 | 0.896 |  |
| *Cerdocyon thous* | Maximun test sensitivity plus specificity Cloglog threshold | 0.1852 | 0.801 |  |
| *Coendou*  *baturitensis* | 10 percentile training presence Cloglog threshold | 0.6237 | 0.994 |  |
| *Coendou*  *prehensilis* | Equal training sensitivity and specificity Clolog threshold | 0.2566 | 0.833 |  |
| *Conepatus semistriatus* | Fixed comulative value 10 Cloglog threshold | 0.2211 | 0.852 |  |
| *Cuniculus paca* | Fixed comulative value 5 Cloglog threshold | 0.2291 | 0.815 |  |
| *Dasyprocta leroporina* | 10 percentile training presence Cloglog threshold | 0.3009 | 0.893 |  |
| *Dasyprocta prymnolopha* | 10 percentile training presence Cloglog threshold | 0.3453 | 0.960 |  |
| *Dasypus novemcinctus* | Minimun training presence Clolog threshold | 0.0474 | 0.786 |  |
| *Dasypus septemcinctus* | 10 percentile training presence Cloglog threshold | 0.2202 | 0.928 |  |
| *Tayassu* *pecari* | | Fixed comulative value 5 Cloglog threshold | 0.2266 | 0.806 |
| *Didelphis albiventris* | Balance training omission, predicted area and threshold value | 0.0912 | 0.867 |  |
| *Didelphis marsupialis* | 10 percentile training presence Cloglog threshold | 0.3733 | 0.857 |  |
| *Eira barbara* | Equate entropy of thresholded and original distribuitions Cloglog threshold | 0.2749 | 0.788 |  |
| *Euphractus sexcinctus* | 10 percentile training presence Cloglog threshold | 0.3483 | 0.892 |  |
| *Galea spixii* | Fixed comulative value 1 Cloglog threshold | 0.1139 | 0.968 |  |
| *Galictis cuja* | Fixed comulative value 1 Cloglog threshold | 0.0304 | 0.902 |  |
| *Galictis vittata* | 10 percentile training presence Cloglog threshold | 0.2710 | 0.862 |  |
| *Herpailurus yagourondi* | 10 percentile training presence Cloglog threshold | 0.2881 | 0.855 |  |
| *Hydrochoerus hydrochoaeris* | Minimun training presence Clolog threshold | 0.1464 | 0.797 |  |
| *Kerodon rupestris* | 10 percentile training presence Cloglog threshold | 0.3063 | 0.977 |  |
| *Leopardus emiliae* | 10 percentile training presence Cloglog threshold | 0.1889 | 0.929 |  |
| *Leopardus pardalis* | Balance training omission, predicted area and threshold value | 0.1565 | 0.791 |  |
| *Leopardus wiedii* | Fixed comulative value 5 Cloglog threshold | 0.1437 | 0.871 |  |
| *Lontra longicaudis* | Fixed comulative value 5 Cloglog threshold | 0.1921 | 0.830 |  |
| *Lycalopex vetulus* | 10 percentile training presence Cloglog threshold | 0.3582 | 0.965 |  |
| *Mazama americana* | Minimun training presence Clolog threshold | 0.1896 | 0.804 |  |
| *Mymercophaga tridactyla* | Minimun training presence Clolog threshold | 0.1553 | 0.792 |  |
| *Nasua nasua* | 10 percentile training presence Cloglog threshold | 0.4201 | 0.784 |  |
| *Ozotoceros bezoarticus* | Fixed comulative value 10 Cloglog threshold | 0.2406 | 0.923 |  |
| *Panthera onca* | Fixed comulative value 5 Cloglog threshold | 0.2263 | 0.805 |  |
| *Dicotyles tajacu* | 10 percentile training presence Cloglog threshold | 0.4051 | 0.789 |  |
| *Priodontes maximus* | Fixed comulative value 5 Cloglog threshold | 0.1809 | 0.873 |  |
| *Procyon cancrivorus* | Balance training omission, predicted area and threshold value | 0.1260 | 0.844 |  |
| *Puma concolor* | menor do que o treshould | 0.0700 | 0.779 |  |
| *Sapajus apella* | Fixed comulative value 5 Cloglog threshold | 0.2363 | 0.807 |  |
| *Sapajus libidinosus* | Fixed comulative value 10 Cloglog threshold | 0.1598 | 0.953 |  |
| *Sapajus xanthosternos* | Equate entropy of thresholded and original distribuitions Cloglog threshold | 0.2477 | 0.905 |  |
| *Speothos venaticus* | Minimun training presence Clolog threshold | 0.1205 | 0.848 |  |
| *Subugulo gouazoubira* | 10 percentile training presence Cloglog threshold | 0.3072 | 0.840 |  |
| *Sylvilagus brasiliensis* | Fixed comulative value 5 Cloglog threshold | 0.1763 | 0.833 |  |
| *Tamandua tetradactyla* | Fixed comulative value 5 Cloglog threshold | 0.2697 | 0782 |  |
| *Tapirus terrestris* | Fixed comulative value 10 Cloglog threshold | 0.3576 | 0.788 |  |
| *Tolypeutes tricinctus* | Minimun training presence Clolog threshold | 0.5065 | 0.975 |  |
