# Supplementary figures and images for: Reconstructing the degree of mammal defaunation throughout the Caatinga - the largest dry tropical forest region of South America
Source: PLoS One. 2025 Nov 24;20(11):e0336562. doi: 10.1371/journal.pone.0336562 (PMC12643294; doi:10.1371/journal.pone.0336562)

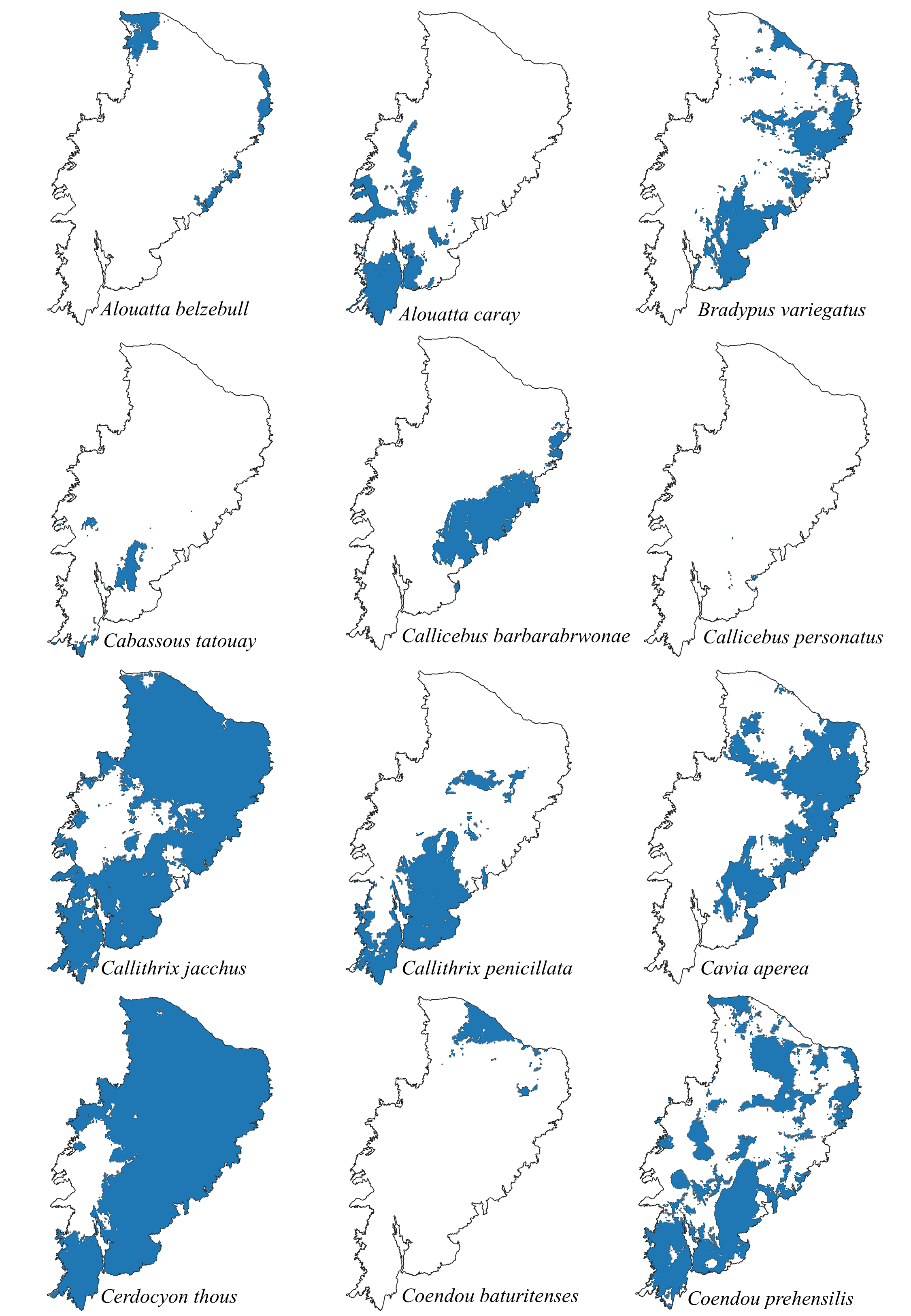

Supplement: S1 Fig — (TIF) [file pone.0336562.s005.TIF]

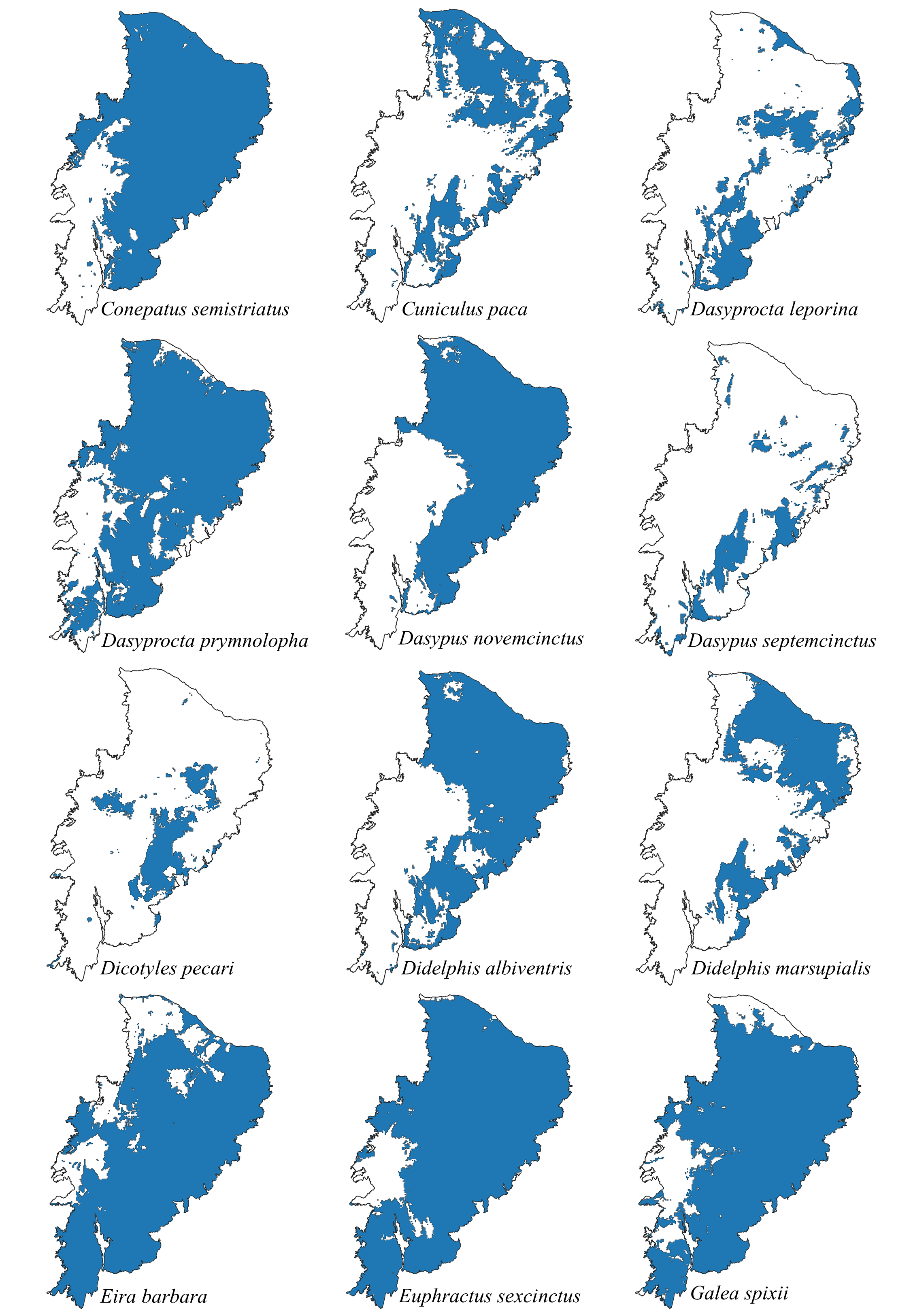

Supplement: S2 Fig — (TIF) [file pone.0336562.s006.TIF]

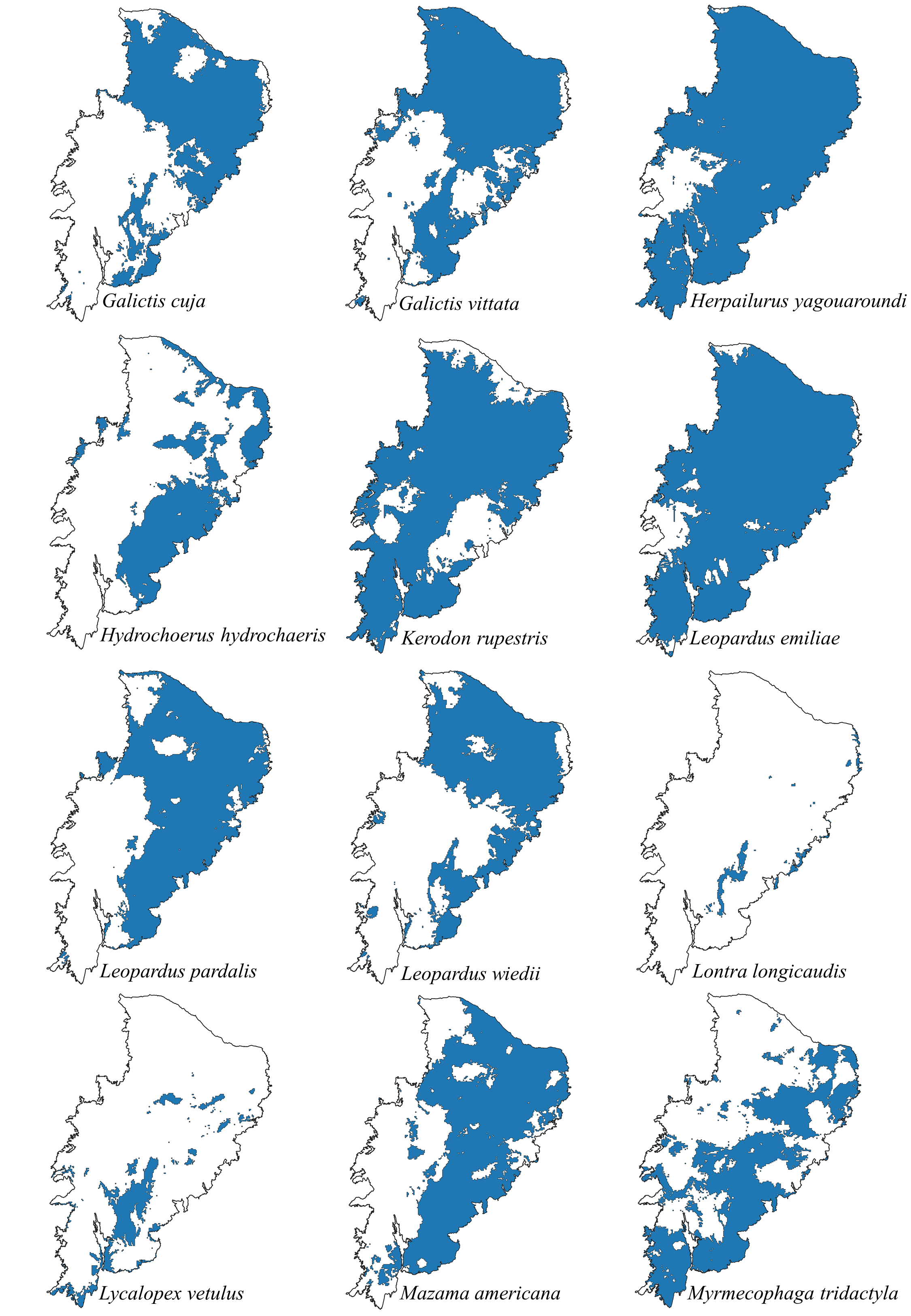

Supplement: S3 Fig — (TIF) [file pone.0336562.s007.TIF]

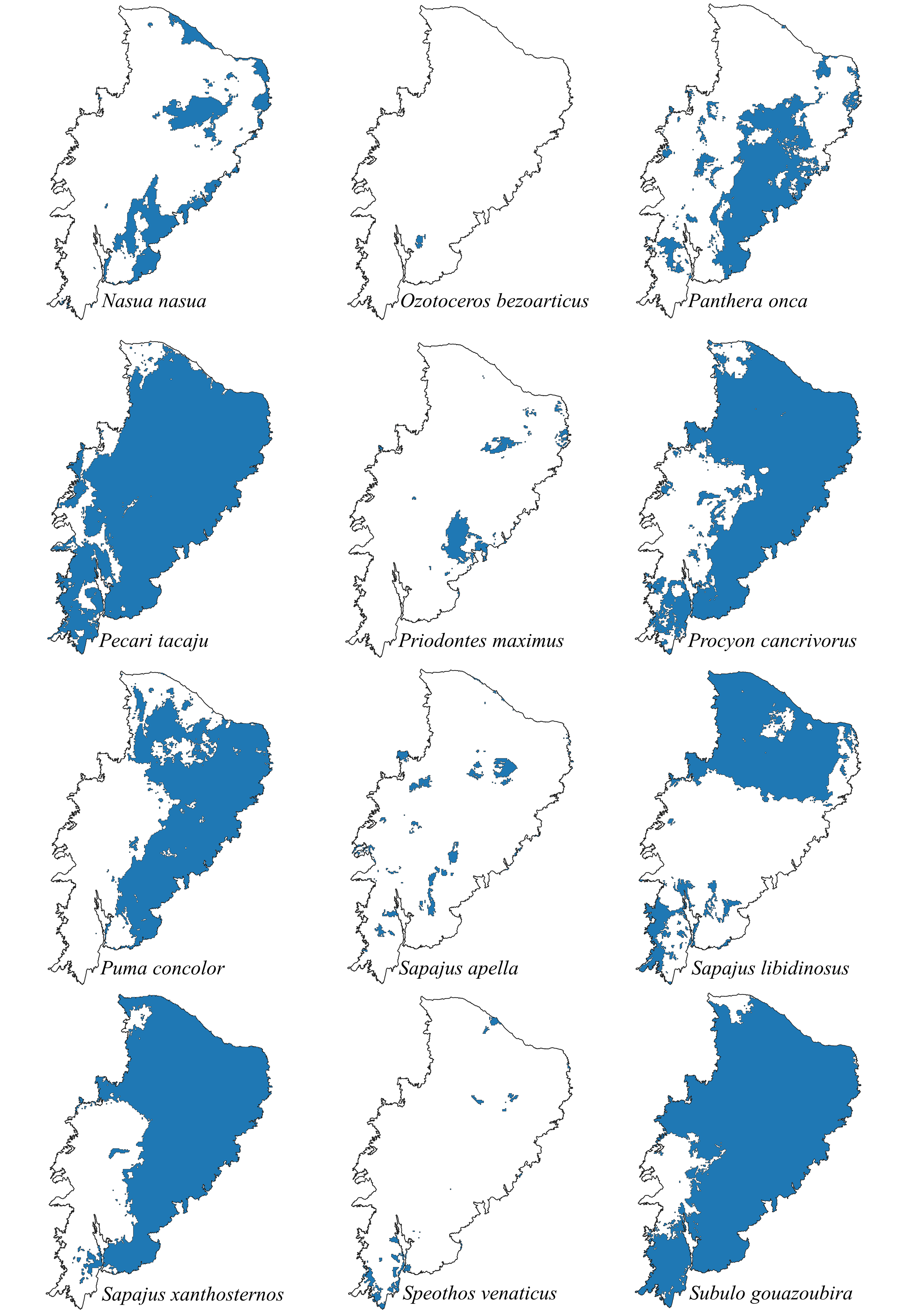

Supplement: S4 Fig — (TIF) [file pone.0336562.s008.TIF]

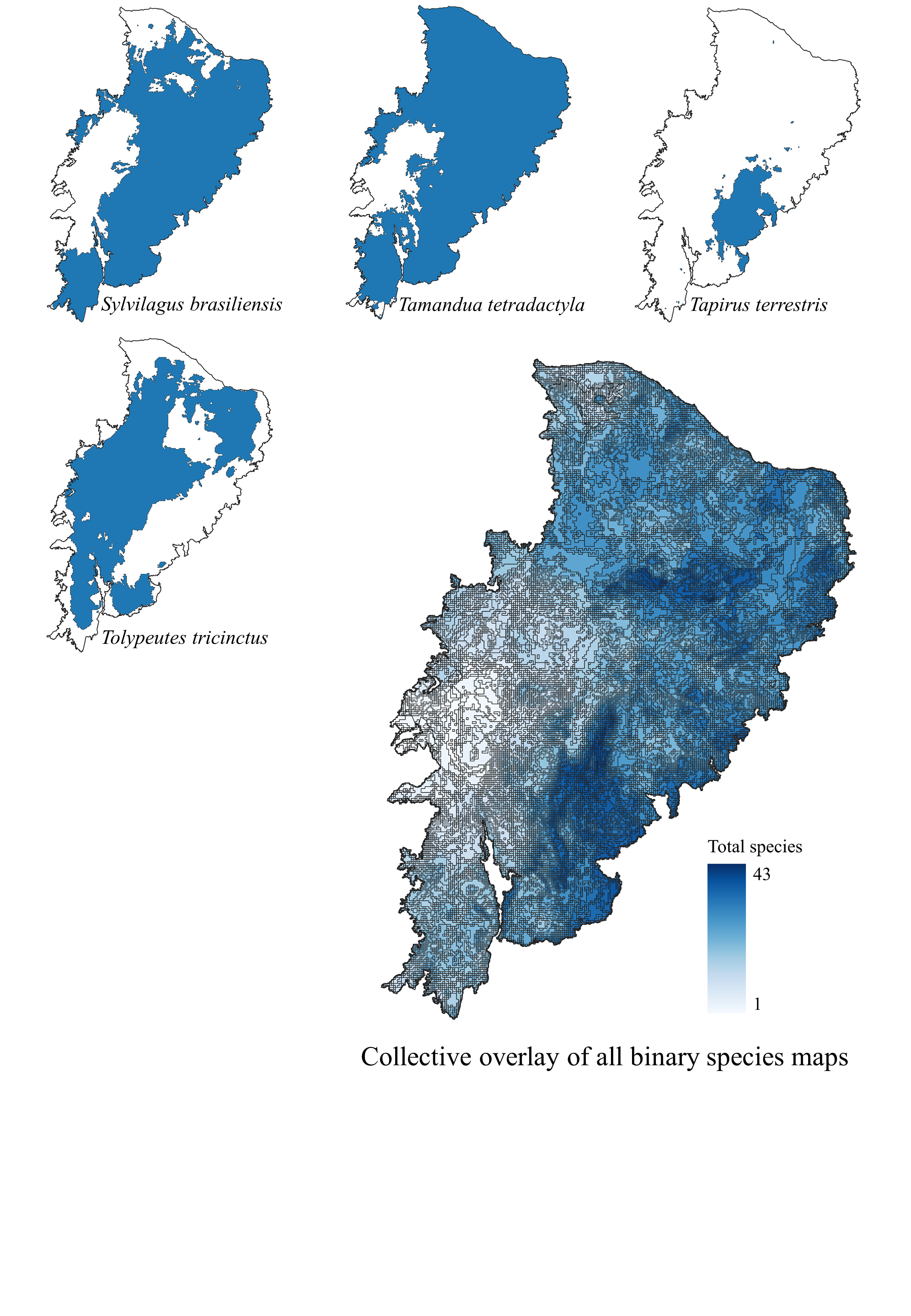

Supplement: S5 Fig — (TIF) [file pone.0336562.s009.TIF]
